# A Scoping Review of Dingo and Wild-Living Dog Ecology and Biology in Australia to Inform Parameterisation for Disease Spread Modelling

**DOI:** 10.3389/fvets.2019.00047

**Published:** 2019-03-05

**Authors:** Vanessa Gabriele-Rivet, Julie Arsenault, Barbara Wilhelm, Victoria J. Brookes, Thomas M. Newsome, Michael P. Ward

**Affiliations:** ^1^Sydney School of Veterinary Science, Faculty of Science, The University of Sydney, Camden, NSW, Australia; ^2^Département de Pathologie et Microbiologie, Faculté de Médecine Vétérinaire, Université de Montréal, Saint-Hyacinthe, QC, Canada; ^3^Big Sky Health Analytics, Vermilion, AB, Canada; ^4^School of Life and Environmental Sciences, Faculty of Science, The University of Sydney, Sydney, NSW, Australia

**Keywords:** scoping review, dingo, wild dogs, disease modelling, Australia, rabies

## Abstract

**Background:** Dingoes and wild-living dogs in Australia, which include feral domestic dogs and dingo-dog hybrids, play a role as reservoirs of disease. In the case of an exotic disease incursion—such as rabies—these reservoirs could be a threat to the health of humans, domestic animals and other wildlife in Australia. Disease spread models are needed to explore this impact and develop mitigation strategies for responding to an incursion. Our study aim was to describe relevant information from the literature, using a scoping review, on specific topics related to dingo and wild-living dog ecology and biology (topics of interest) in Australia to inform parameterisation of disease spread modelling and identify major research gaps.

**Methods:** A broad electronic search was conducted in five bibliographic databases and grey literature. Two levels of screening and two levels of data extraction were each performed independently by two reviewers. Data extracted included topics of interest investigated, type of population sampled, the presence of lethal control, type of environment, years of collection and GPS coordinates of study sites.

**Results:** From 1666 records captured, the screening process yielded 229 individual studies published between 1862 and 2016. The most frequently reported topics of interest in studies were index of abundance (*n* = 93) and diet (*n* = 68). Among the three key parameters in disease spread modelling (i.e., density, contacts and home range), data on density and contacts were identified as major research gaps in the literature due to the small number of recent studies on these topics and the scarcity of quantitative estimates. The research reviewed was mostly located around central Australia and the east coast, including a few studies on density, contacts and home range. Data from these regions could potentially be used to inform parameterisation for disease spread modelling of dingoes and wild-living dogs. However, the number of studies is limited in equatorial and tropical climate zones of northern Australia, which is a high-risk area for a rabies incursion.

**Conclusions:** Research in northern regions of Australia, especially to generate data regarding density, contacts and home ranges, should be prioritised for future research on dingoes and wild-living dogs.

## Introduction

Disease spread modelling is a powerful tool used to predict the spread of infectious diseases and evaluate interventions to mitigate disease impact (1). The study of infectious disease dynamics using models has substantial value in preparedness planning in the case of exotic disease incursions and to inform policy decisions. To develop a model structure which reflects reality, it is essential to understand the natural system which governs disease dynamics and to identify parameters to describe disease spread patterns within a population. In addition, accurate data for parameterisation is critical to generate valid model outputs. This task is challenging when data are scarce or limited; such research gaps can compromise the reliability of model predictions and therefore assessment of control strategies.

Ecological and biological parameters describing how animals within the population interact in space and time are embedded within the core of any disease spread model involving free-roaming animals. Animal density, home range and contacts are three key ecological features for wildlife disease spread modelling (2, 3). Contact between animals is a fundamental driver of disease transmissions both spatially and temporally. Likewise, the number of animals living within a specific geographic area and their home range size can both have a considerable impact on the frequency of encounters with neighbouring individuals via the amount of spatial overlap (4). Disease dynamics also vary depending on other parameters related to animal movement, such as dispersal behaviour (5). Information in relation to environmental requirements or habitat preference can generate carrying capacity indices for mapping animal distribution when developing spatial disease spread models (6, 7). Accurate information about reproduction, mortality rate and life span—which all have an impact on population density and population structure over time—can improve disease predictions over relatively long time periods (8). Finally, these parameters might also vary depending on season or different types of groups within the host population, such as age and sex (8–11).

Rabies is one such disease for which disease spread modeling is useful. Caused by lyssaviruses of the Rhabdoviridae family, rabies is characterised by an acute encephalitis in mammals with a case fatality rate of nearly 100% following the onset of clinical symptoms (12). Dogs remain the main source of rabies transmission globally to humans, with more than 99% of human cases caused by dog bites (13). Although rabies transmission mainly involves urban dogs, wild canids are assumed to be primary hosts of the virus in a number of countries throughout the world and have occasionally been implicated in the transmission of rabies to other species, including other wildlife, domestic animals and humans (14–17).

Although terrestrial rabies is exotic to Australia—only Australian bat lyssavirus is present (18)—the continent is currently threatened by the spread of canine rabies eastward along the Indonesian archipelago, with outbreaks occurring in previously rabies-free Indonesian islands such as Flores, Ambon, and Bali (19–21). Therefore, northern Australia is a region at relatively high risk of a canine rabies incursion. The most likely incursion scenario would include the illegal movement of a sub-clinically infected dog entering Australia by boat as a result of cultural ties between community groups in South-East Asia and Australia, via a fishing vessel or a pleasure craft (21). Of particular concern, with respect to canine rabies transmission is Australia's large wild-living dog population, which is widespread throughout most of mainland Australia. This population consists of a mixture of dingoes (*Canis dingo)*, feral domestic dogs (*Canis familiaris*) and an expanding population of dingo-dog hybrids (referred to collectively as dingoes and wild-living dogs hereafter) due to extensive interbreeding between the two species (22). These animals have the ability to roam over large distances (more than 200 kilometers) and co-occur with various other animal species, such as feral cats (*Felis catus*), red foxes (*Vulpes vulpes*), and quolls (*Dasyurus* spp.), which could facilitate the spread of rabies (23–26). Dingoes and wild-living dogs are also found in proximity to humans: some populations live in and around cities and towns as well as within tourist areas (27, 28). In the case of a rabies incursion, infection within these populations would represent a serious threat to humans, domestic animals and other wildlife in Australia.

Parameterisation of disease spread models can be informed using knowledge synthesis methodologies, which employ a systematic, thorough and transparent approach to identify and summarise all relevant literature on a research question (29, 30). A scoping review is a relatively new and increasingly popular research synthesis approach, used in a vast range of fields and disciplines (31). Scoping reviews fundamentally differ from systematic reviews, which are the most widely used knowledge synthesis method, by their distinct set of purposes. As opposed to systematic reviews (which aim to collate empirical evidence by addressing a specific research question), the main goal of a scoping review is to map the body of literature pertaining to a broad or complex topic. They typically do not assess the quality of the studies included in the review. Scoping reviews are particularly useful in areas which have never been reviewed previously to give a complete overview of research activity (32, 33).

Assessing the knowledge base pertaining to dingo and wild-living dog ecology and biology in Australia is critical as a first step to evaluate whether studies exist to potentially permit parameterisation of disease spread models. Therefore, due to the vast extent of the area of interest, a scoping review was conducted to map the existent information, as well as to identify major research gaps in the ecology and biology of dingoes and wild-living dogs in Australia.

## Methods

### Research Question

The process steps employed in this review were based on the methodological framework for conducting scoping reviews proposed by Arksey and O'Malley (32), and we incorporated recommendations described by Levac et al. (33) and Daudt et al. (34). Although the PICO (Population, Intervention, Comparison and Outcomes) tool is widely used in systematic reviews and critically appraised for defining a narrow, clear and focused research question, this format was not suitable for the present review because our area of interest is considerably broad (35). The following research question was formulated based on the intended purpose of this study:

“For the purposes of disease spread modelling, what is the current state of knowledge of the ecology and biology of dingoes and wild-living dogs in Australia?”

The population of interest in this review includes dingoes, feral domestic dogs, and dingo-dog hybrids in Australia. Owned domestic dogs were specifically excluded. Twenty-eight topics of interest, which were considered potentially useful to parameterise disease spread modelling, were selected. These included: density or population size (referred to as “density” herein), index of abundance or index of density or index of activity (referred to as “index of abundance” herein), quantitative observations of live individuals, habitat or landscape preference, natural landscape barriers, diet, water requirements, birth rate, litter size, litter sex ratio, reproductive age, gestation time, birth period, mating period, mortality rate, life span, contacts or interactions between dingoes, and/or wild-living dogs (referred to as “contacts” herein), home range, dispersal or migration, regular movement patterns, long distance movement outside the home range, age of independence, daily patterns of movement or activity, seasonal patterns of density, movement or activity, group size, sex distribution, age distribution, and percentage of hybridisation within a population. Inclusion criteria were any primary research (whether peer-reviewed or not) published in English in scientific journal articles, research reports from Australian government agencies, conference papers, theses or book chapters that reported data on at least one topic of interest from one of the populations of interests within Australia. Annual and progress reports from government or research agencies were excluded to avoid duplicating data.

### Search Strategy

For the purpose of this review, the term “record” is used to describe any bibliographic citation captured through the initial search. A “study” is defined as an individual scientific article, report, thesis chapter, book chapter, or conference paper in a conference proceeding. For the most part, each record is considered a study. In the case in which the record contains multiple chapters or papers (for example entire theses, books and conference proceedings), each chapter or article was treated as one individual study. The term “data of interest” is used hereafter to describe all data collectively reported in a study related to a specific topic of interest.

The initial search was implemented in November 2016 using five electronic databases: Web of Science Core Collection, BIOSIS Previews, CAB Abstracts, Zoological Record, and SCOPUS. All years of publication were included. The initial search was conducted using the following combination of terms:

- Population: “Wild dog^*^” OR Dingo^*^ OR “Feral dog^*^” OR “*Canis lupus familiaris*” *OR* “*Canis lupus dingo*” OR “Free roaming dog^*^” OR “Free-roaming dog^*^” OR “Free ranging dog^*^” OR “Free-ranging dog^*^” OR “Stray dog^*^” OR “*Canis familiaris* dingo” OR “*Canis familiaris familiaris*” OR “Wild-living dog” OR “Commensal dog” OR “*Canis dingo*” OR “*Canis antarticus*” OR “*Canis familiaris australasiae*” OR “*Canis australiae*” OR “*Canis dingoides*” OR “*Canis macdonnellensis*” OR “*Canis familiaris*” OR “Wild canid”
- Location: Australia^*^
- Topic: Ecolog^*^ OR Biolog^*^ OR Densit^*^ OR Distribution^*^ OR Population^*^ OR Demograph^*^ OR Habitat^*^ OR Landscape^*^ OR Diet^*^ OR Water^*^ OR Birth^*^ OR Gestation OR Litter^*^ OR Mating^*^ OR Reproduct^*^ OR Mortalit^*^ OR “Life span” OR “Lifespan” OR “Life-span” OR “Contact rate” OR “Home range” OR “Home-range” OR Dispersal OR Movement^*^ OR Age OR Vegetation OR Breeding^*^ OR Dynamic^*^ OR Interaction^*^ OR Behavior^*^ OR Sex OR Predat^*^ OR Competition OR Trophic^*^ OR Mesopredator^*^ OR Activit^*^ OR Genetic^*^ OR Hybridisation OR Den^*^ OR Breeding

As a complementary search, the first 100 results from the search engine “Google” were screened to identify any relevant records that were not previously captured. Conference papers index via Proquest and Trove were used to search for conference proceedings and theses and dissertations, respectively, using the same algorithms as described above. Australian government and research organisation websites [Commonwealth Scientific and Industrial Research Organisation (CSIRO), Invasive Animals Cooperative Research Centre (Invasive Animals CRC), Department of Agriculture for each Australian state] were searched manually for additional relevant reports. Additionally, emails were sent to Australian government agencies, CSIRO and Invasive Animals CRC to request any reports or unpublished material potentially relevant to the present scoping review. A verification of our search strategy was undertaken by screening the bibliography list of 15 key documents, which were selected from the reference list obtained following the first level of screening by targeting recent papers, book chapters or theses, based on their relevance to the range of topics of interest. Any reference from the bibliography lists which were potentially relevant to our study were included in the review process and screened in the same manner as records captured from the database search. Additionally, an expert was asked to screen our list for any missing relevant references. Records captured by the search were exported into Endnote, a citation management software. Duplicated records were removed. The final list was uploaded to the web-based electronic review platform DistillerSR (Evidence Partners Incorporated, Ottawa, ON) for screening and data extraction.

### Levels of Screening and Data Extraction

A four-level screening and data extraction process was implemented. The first level of screening was undertaken to eliminate irrelevant articles based on the abstract (or the title when the abstract was not available). All records that met the eligibility criteria from the first screening level were advanced to the second level in which detailed screening was performed on the full-text documents. All records that met the eligibility criteria from the second screening level were advanced to the third level for further data extraction. A fourth level was developed specifically to extract quantitative data from studies investigating one of three key topics for disease spread modelling, i.e., density (including population size), home range and contacts (including interactions between packs or individuals and indices of probability of contact). In the case in which the data from research was published in multiple documents (for example data published in a thesis chapter and a journal article), the publication providing the most exhaustive description of the data was selected for data extraction.

Data recorded included (1) characteristics of the study (type of document, year of publication and type of topic of interest investigated) and (2) characteristics of the data of interest. The latter included relevance of the data reported in relation to the objectives of the study (related to the objectives of the study or peripheral/supplementary data), type of population sampled (dingo, feral dog, hybrid, unknown sample, mixed sample), type of environment in which the data was collected (rural or natural, urban or peri-urban, mine sites, animals in captivity, one or multiple entire states or territories or not reported), presence of lethal control targeted at dingoes and wild-living dogs and applied within the environment (with control, without control, with and without control, not applicable, not reported), geographic location of study sites and year of data collection. For each study, the type of population sampled was categorised based entirely on the terms or descriptions provided by authors. Samples categorised as “Unknown sample” contained an unknown mixture of animals in which it was not established if dingoes, feral domestic dogs, hybrids or a combination of these comprised the sample. Samples categorised as “Mixed sample” comprised a known mixture of dingoes, feral domestic dogs, or hybrids but the data of interest was reported in an aggregated form. All other categories (i.e., dingo, feral dog, and hybrid) were selected when the data of interest were reported separately for each type of dog. The information recorded in the fourth level included the density, home range and contact estimates provided in each study as well as a brief description of the methods used.

For the first three levels, a form was developed and tested *a priori* on a subset of records (50 in first level, 30 in second level, and 12 in third level) by all five reviewers participating in the screening process to evaluate reviewers' agreement. If needed, modifications were made to improve the structure of the form and the clarity of the questions. A level of agreement of 0.80 (Cohen's kappa statistic) was obtained in the pre-tests between reviewers before starting each level of screening or data extraction. Forms at the fourth level were not pre-tested because the data extraction was straightforward and the number of studies included in the level was small. Records at all levels of the review were independently assessed by two reviewers. Conflicts were resolved by discussion between the relevant reviewers, until consensus was reached. The protocol, forms and guidelines used to assist in completing each level are in Appendices A–C (Supplementary Material).

### Data Analyses

All data captured from each level were exported to Excel and subsequently imported into SAS version 9.3 (SAS Institute Inc., Cary, NC) to perform data manipulation and analyses.

Descriptive statistics were used to summarise the number of studies in terms of type of document. The number of studies reporting data on each specific topic of interest was calculated and plotted according to data relevance, type of population sampled, type of environment, presence of lethal control, and year of data collection category (≤ 1950, 1951–1960, 1961–1970, 1971–1980, 1981–1990, 1991–2000, 2001–2010, and 2011–2016). The total number of studies by year of data collection and year of publication were also calculated. Studies that spanned more than one time period (year of data collection category or year of data collection) were counted once in each corresponding time period.

### Mapping

For the purpose of mapping, a site was defined as a geographical area in which data of interest was collected, as described by the authors. A study could contain one or more sites. When a study provided the location of each single sample collected (for example a scat, DNA sample, or carcass) or the location of each individual trap, camera-trap, sandplot or transect, these locations were considered as a group and represented as one site. Sites which were divided into two sub-sites (for example, sites that had sub-sites on either side of the 5,500 km long dingo fence or sites that comprised a sub-site with lethal control and a sub-site without lethal control) were considered as one single site. This approach was considered suitable for our review, because our intent was to identify and map studies and topics of interest, rather than to compare and contrast the results within studies. The location of each site was chosen as the best representation of the center of the site. Whenever the author provided the GPS coordinates associated with the center of a site, this information was used to locate that site. Otherwise, the center location of each site was identified as accurately as possible using all information supplied by the authors. These GPS points collectively are referred to as “site points” hereafter. Studies conducted across one or more states or territories or across Australia were not included in the site points dataset.

The distribution of site points was mapped in relation to the six major Köppen climate classification zones using the “Köppen climate classification” dataset from the Bureau of Meteorology of Australia (36), at a resolution of 0.025° (~2.5 km). These climate zones, which are based predominantly on native vegetation type, were extracted for each site point. The number of studies conducted in each climate zone was subsequently calculated, with each study counted only once per climate zone.

The geographical distribution of number of studies, number of topics of interest reported and number of studies on the three key topics for disease spread modelling, i.e., density, home range, contacts, was depicted using unclassified choropleth maps. A regular hexagonal grid was first created in QGIS version 2.18 (37) using a distance of 200 km as the short diagonal of the hexagon (area = 34 641 km^2^) and overlaid on Australia. The hexagon grid was chosen to enhance visual patterns which may be more difficult to discern with rectangular grids (38). Using the dataset of site points, the number of different studies or different topics of interest contained within each hexagon was summed, with each study or topic of interest counted only once per cell. Studies covering entire states/territories or areas larger than ~34,000 km^2^ were attributed to one or more cells as applicable. All mapping, with the exception of the creation of the hexagonal grid, were performed using ArcGIS version 10.3.1 (ESRI, Redlands, CA). The Geocentric Datum of Australia 1994/Geoscience Australia Lambert projection (GDA_1994_Geoscience_Australia_Lambert) was used for all layers.

## Results

**Figure 1:**
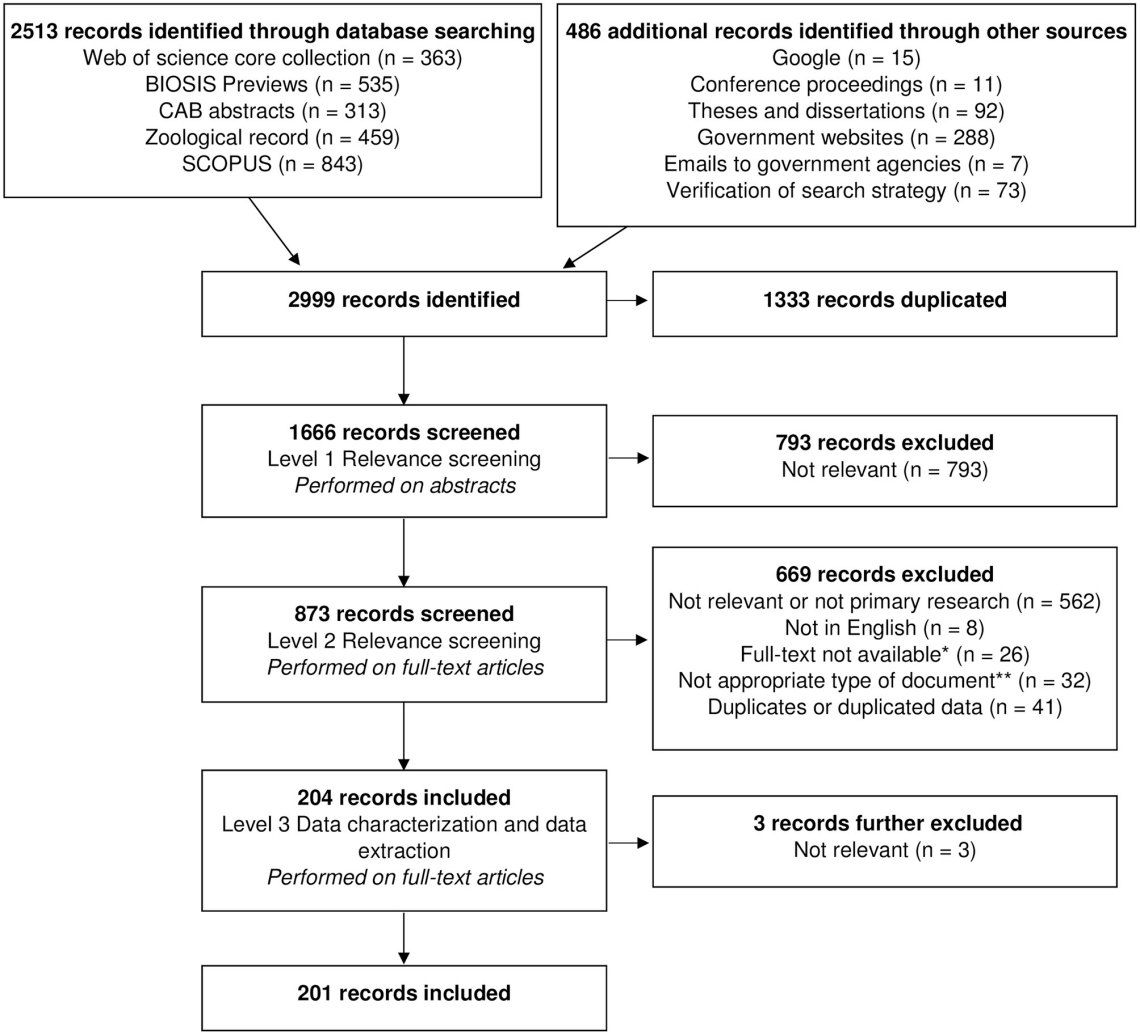
Flow chart of a scoping review focused on the ecology and biology of dingoes, feral domestic dogs, and dingo-dog hybrids in Australia, targeting 28 specific topics of interest selected to inform disease spread models. *Twelve of these records were captured from the “Verification of search strategy.” ^**^Includes 15 progress reports or annual reports from government or research agencies.

**Figure 2:**
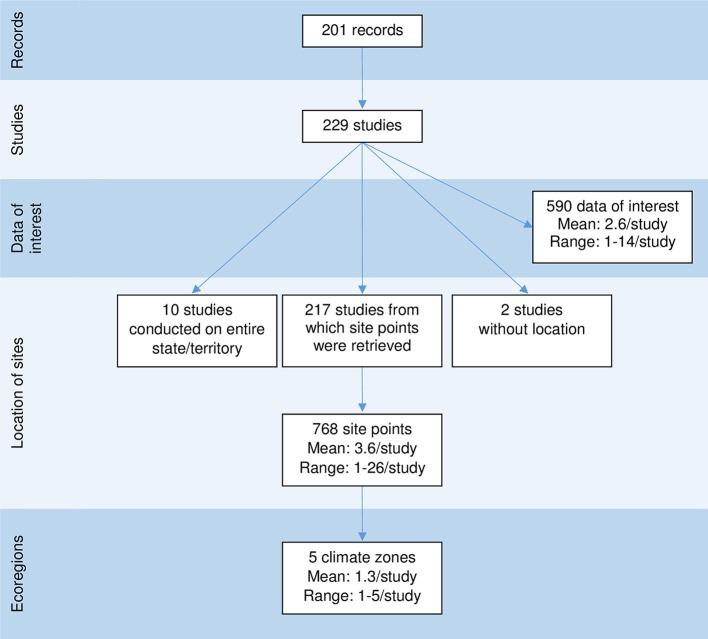
Diagram illustrating the links between the number of records, studies, data of interest, site points, and ecoregions, captured in a scoping review on the ecology and biology of dingoes, feral domestic dogs, and dingo-dog hybrids in Australia, targeting 28 specific topics of interest selected to inform disease spread models.

Figure 1 shows a flow chart of the number of records identified at each stage of the scoping review. Following removal of all duplicates, the initial search captured a total of 1,666 records, including 73 records retrieved from our search verification by screening the bibliographic list of the 15 selected documents (no additional records were identified by the expert). There were 793 (47.6% of 1,666) records excluded in level 1 because either the population, location or the topics studied did not meet our inclusion criteria. Most of the records excluded in level 2 were not relevant to our research question or not categorised as primary research (*n* = 562; 64.3% of 873 records from level 2). Other reasons for exclusion at this level included duplicated data (*n* = 41; 4.7% of 873 records from level 2), inappropriate type of document (*n* = 32; 3.7% of 873 records from level 2), lack of availability of the full-text document (*n* = 26; 3.0% of 873 records from level 2), and foreign language of publication (*n* = 8; 0.9% of 873 records from level 2). Following all three levels of screening and data extraction, this review yielded a total of 201 records (23.0% of 873 records from level 2), including six records captured from the search verification. From these records, we retrieved 229 individual studies published from 1862 to 2016 (Figure 2). A list of all studies included in this review can be found in Appendix D (Supplementary Material) with (if applicable) the corresponding complementary studies reporting the same data of interest. Studies consisted mostly of scientific journal articles (*n* = 142; 62.0% of the 229 studies captured within our dataset), followed by thesis chapters (*n* = 46; 20.1%), government or agency reports (*n* = 25; 10.9%), conference papers (*n* = 10; 4.4%), and book chapters (*n* = 6; 2.6%).

**Figure 3:**
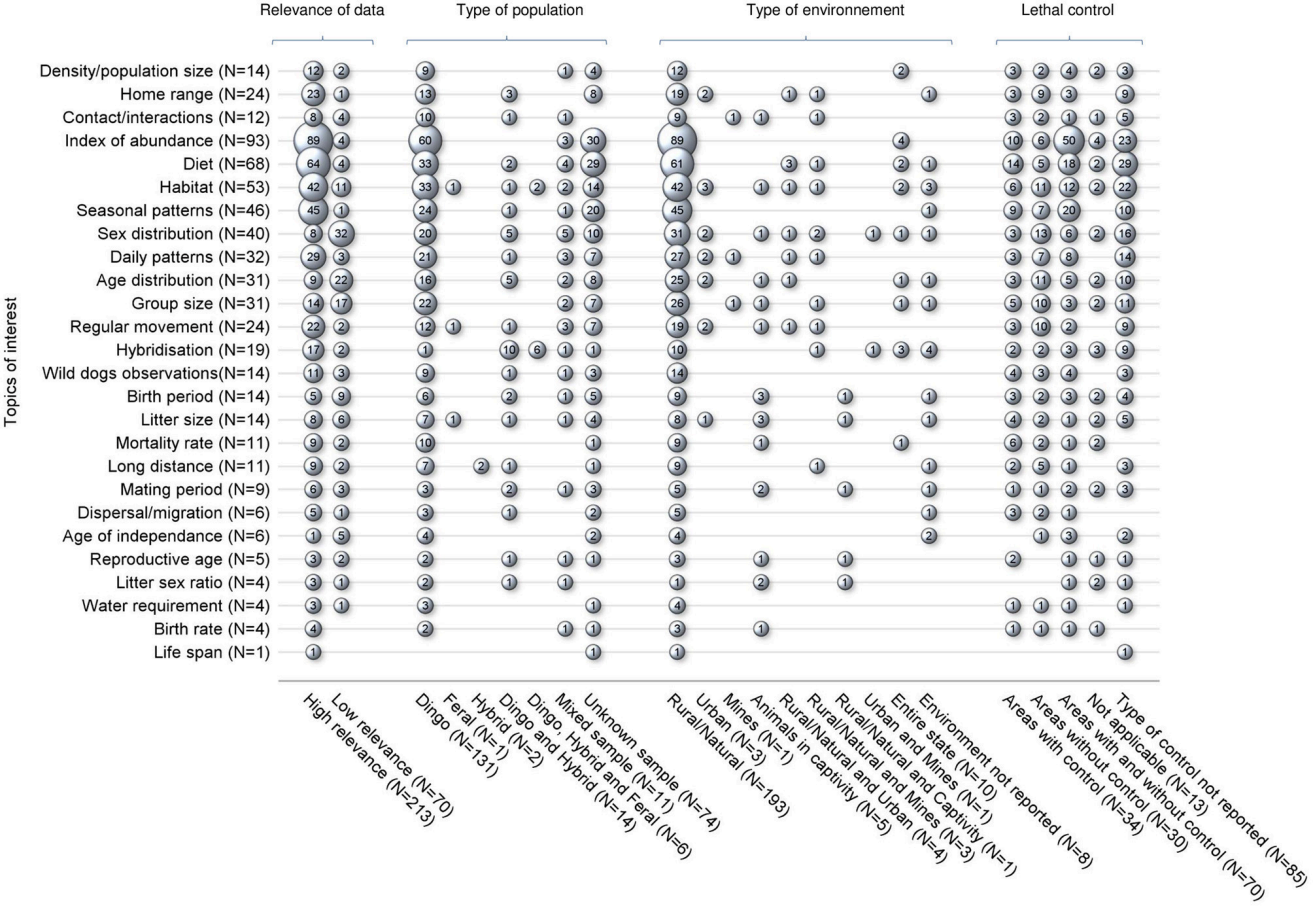
Bubble plot of 229 studies captured from a scoping review on the ecology and biology of dingoes, feral domestic dogs, and dingo-dog hybrids in Australia, targeting 28 specific topics of interest selected to inform disease spread models, in relation to four groupings of data of interest characteristics. Bubble sizes are directly proportional to the number of studies. For each characteristic group, the sum of bubble sizes on each line corresponds to the number of studies reporting data on that particular topic of interest (N). The sum of bubble sizes in each column corresponds to the number of data of interest reported in the literature within that category; N stands for the number of studies reporting at least one data of interest from the corresponding category.

These 229 studies generated 590 data covering all topics of interest except natural landscape barrier and gestation period, for which no pertinent studies were retrieved. The number of data of interest reported per study ranged between 1 and 14 (mean 2.6) (Figure 2). Seventy-six percent of the data of interest were defined as high relevance, meaning that these data directly addressed the objectives of the study; 213 studies (93.0%) reported at least one data of interest of high relevance, vs. 70 studies (30.5%) that reported at least one data of interest of low relevance (Figure 3). Studies reporting data of interest on dingoes (57.2%, *n* = 131), studies collecting data of interest from a rural or natural setting (84.3%, *n* = 193), and studies which did not document the presence of lethal control in the environment (37.1%, *n* = 85) were the most frequent categories. The two most frequently reported topics of interest were the index of abundance (40.6%, *n* = 93) and diet (27.5%, *n* = 63). Density, home range and contacts were investigated in 14, 24, and 12 studies, respectively, for which 86, 66, and 96% of the studies reported “High” relevance data of interest. Most studies regarding these key topics collected data of interest from dingoes, in a rural or natural environment. Three studies also investigated home range in an urban setting. The majority of studies investigating density were conducted in locations with and without lethal control, whereas most home range and contact studies did not report the presence of lethal control. Nearly all studies on home range (23/24) provided quantitative estimates, which ranged between 0.37 km^2^ using 95% adaptive kernel in a subtropical climate to 1912.27 km^2^ using 95% fixed kernel in a desert climate zone. In contrast, only five studies on contacts provided quantitative estimates, which included frequency of interactions observed, contact rates, and home range overlap indices. From the 14 studies on density, eight provided quantitative density data of which three reported only a minimum density estimate (Tables E1–E3 of Appendix E in Supplementary Material).

**Figure 4:**
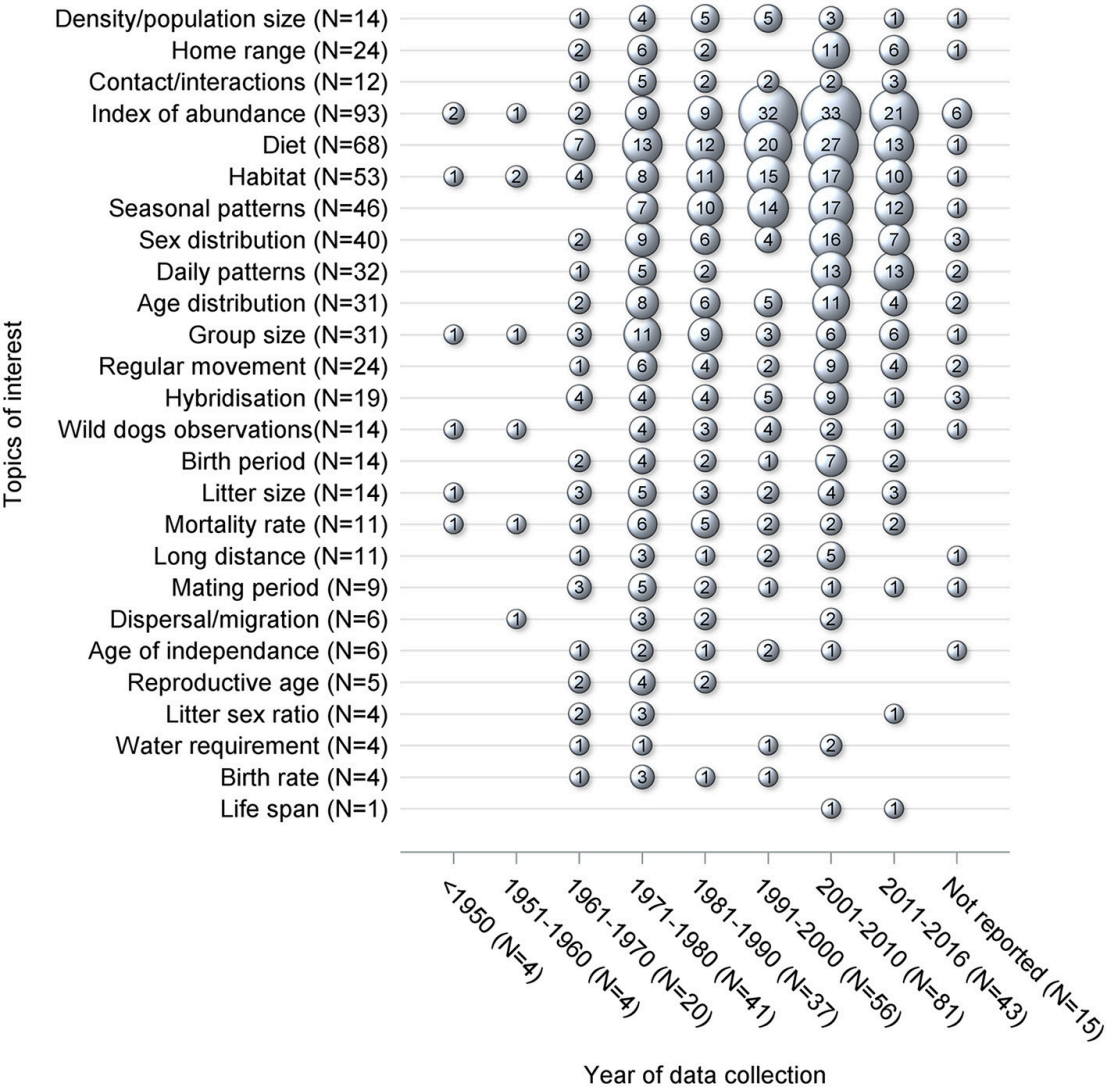
Bubble plot of 229 studies, by year of collection, which were captured from a scoping review on the ecology and biology of dingoes, feral domestic dogs, and dingo-dog hybrids in Australia, targeting 28 specific topics of interest selected to inform disease spread models. Bubble sizes are directly proportional to the number of studies. For each topic of interest, the *N* stands for the number of studies reporting data of interest on the corresponding topic of interest. The sum of the bubble sizes for each column corresponds to the number of data of interest reported in the literature which was collected during that particular time period whereas the *N* stands for the number of studies reporting data of interest which was collected during the corresponding time period.

**Figure 5:**
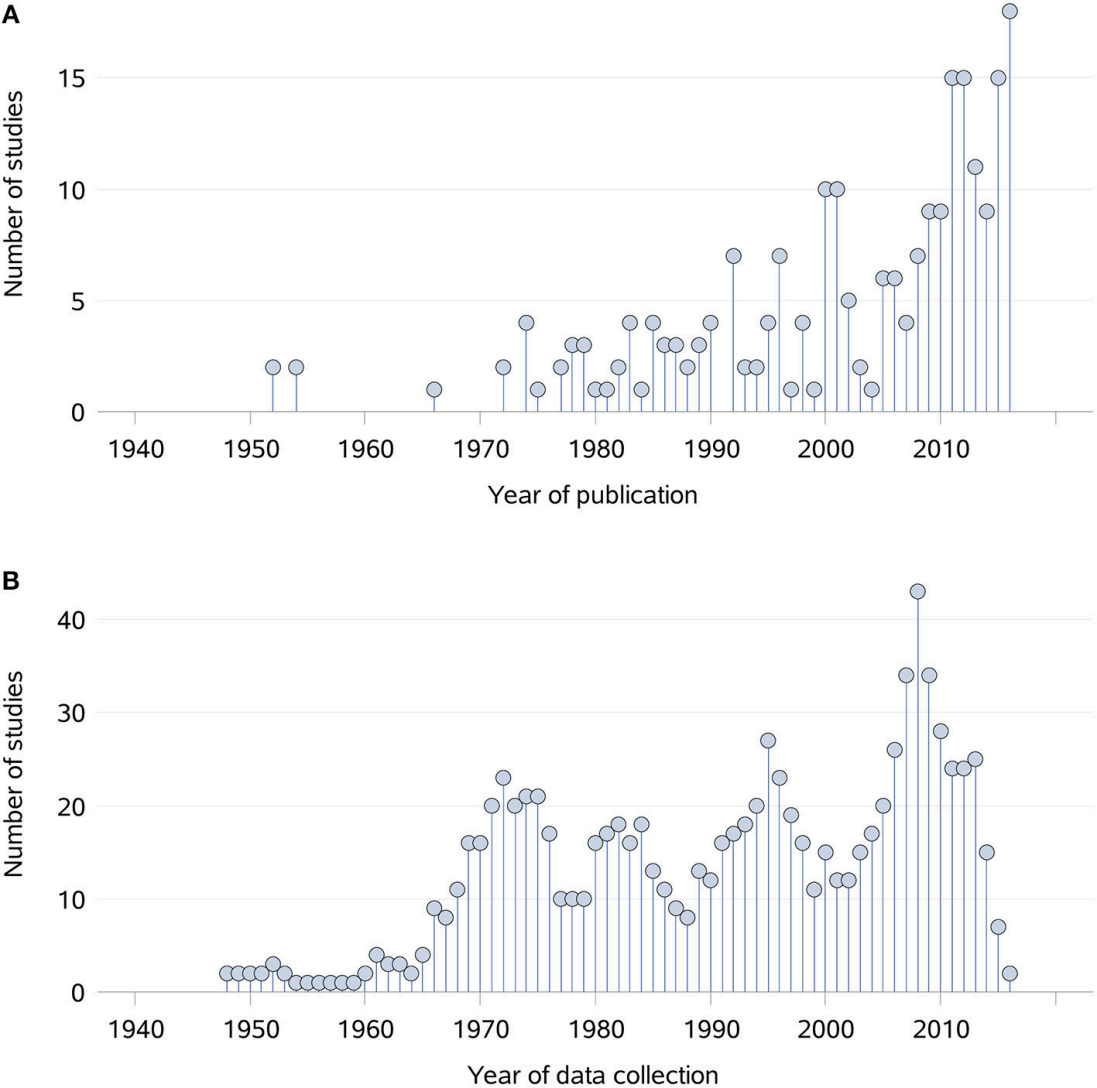
Distribution of the 229 studies captured from a scoping review on the ecology and biology of dingoes, feral domestic dogs, and dingo-dog hybrids in Australia, by year of publication **(A)** and year of data collection **(B)** between 1940 and 2016. Prior to 1940, only one study published relevant data in 1862. During the period 1852–1862 and 1883–1930, the number of studies for which data of interest was collected during each year was equal to one. All other years between 1852 and 1940 did not contribute to the collection of data of interest.

Topics of interest which were frequently reported, such as index of abundance and diet, were consistently investigated in each time period (Figure 4). For other topics there were different patterns in terms of data collection, with peaks occurring in different periods of time. For instance, the majority of data collection on density and contacts occurred prior to 2001, whereas the data of interest on home range was primarily collected in recent years between 2001 and 2016. Overall, an increase in the number of studies published and collecting data of interest is noticeable from the 1960's onwards (Figure 5). The mean difference between the year of publication and the last year of data collection was 4.1 years, ranging from 0 to 70 years, with a median of 2.5 years. Fifteen studies did not report the years in which the data of interest was collected.

**Figure 6:**
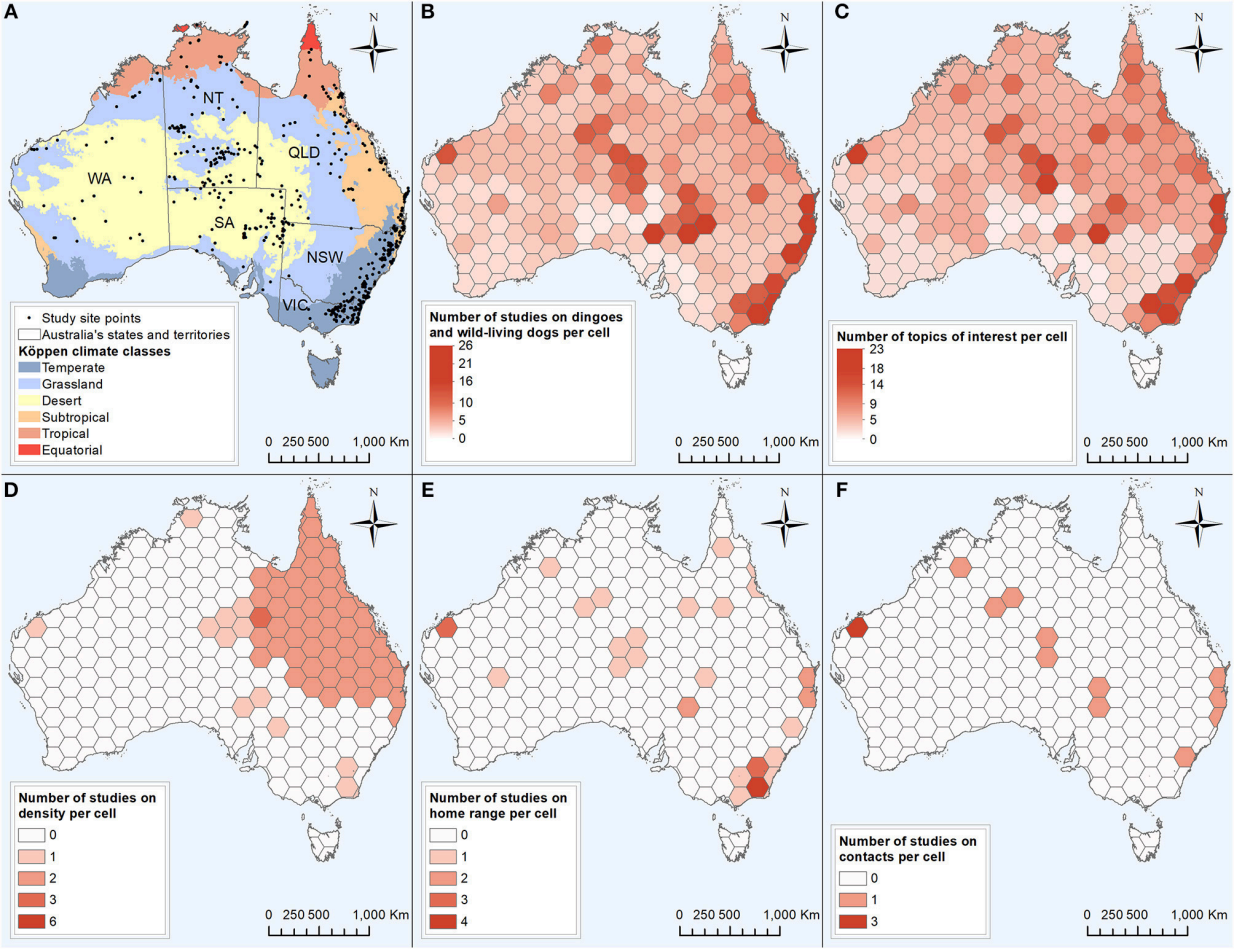
Six maps based on 229 studies captured from a scoping review on the ecology and biology of dingoes, feral domestic dogs, and dingo-dog hybrids in Australia, targeting 28 specific topics of interest selected to inform disease spread models. Colour intensity is proportional to the number of studies conducted **(B,D–F)** or the number of topics of interest reported out of the 28 selected topics of interest **(C)** within the cell. **(A)** Distribution of 768 site points in which data of interest on dingoes and wild-living dogs in Australia was collected, in relation to the Köppen climate major classes (WA, Western Australia; NT, Northern Territory; SA, South Australia; QLD, Queensland; NSW, New South Wales; VIC, Victoria). **(B)** Distribution of studies on dingoes and wild-living dogs in Australia. **(C)** Distribution of reported topics of interest **(D)** Distribution of studies investigating the topic of interest density or population size. **(E)** Distribution of studies investigating the topic of interest home range. **(F)** Distribution of studies investigating the topic of interest contacts or interactions between dingoes and/or wild-living dogs.

The 590 data of interest reported in the literature were collected from 768 site points distributed across all mainland Australian states and territories (Figure 6A). Also contributing to the 590 data of interest reported were ten studies conducted over one or multiple entire states or territories, and two studies which did not provide their study locations (Figure 2). Using the site points dataset, 31.9% of studies (n = 73) had sites categorised as desert Köppen climate class, 30.1% (*n* = 69) in the temperate class, 27.9% (*n* = 64) in the grassland class, 17.9% (*n* = 41) in the subtropical class, 10.0% (*n* = 23) in the tropical class and 0.9% (*n* = 2) in the equatorial class (Figure 6A). Site points which reported data of interest on density and home range occurred in all climate zones except the equatorial zone, whereas data of interest on contacts were reported from site points from all climate zones except the equatorial and tropical zones. In addition, two studies which reported data of interest on density were conducted in the entire state of Queensland. The distribution of dingo and wild-living dog studies and the topics of interest reported appeared to be clustered in the areas of central Australia and the east coast of Australia (Figures 6A–C). Studies on density, home range and contacts were distributed across the states and territories of mainland Australia, with the exception of the state of Victoria for density and contacts (Figures 6D–F). All data extracted can be found in Data Sheets 2, 3 from the Supplementary Material.

## Discussion

This review provides a comprehensive synthesis of literature on dingo and wild-living dog research relevant to the parameterisation of disease spread modelling, and identifies areas in which there are high levels of research activity. Despite the exponential increase in research during the past 20 years, some research gaps were identified in the literature which may present a barrier to disease spread modelling.

The paucity of studies reporting quantitative estimates on density and contacts of dingoes and wild-living dogs in Australia could present a challenge to disease spread modelling. This lack of data might be due to accurate population density estimates and contact rates relying on methods which are often time consuming, complex or expensive, especially when investigating wide-ranging species. As an example, the methods by which density was estimated ranged from a capture-mark-recapture approach (39)—considered a rigorous method for estimating wildlife animal density (40, 41)—to approaches such as counting bonuses paid for dingo scalps (bounties), individuals trapped in an area, sightings from aircraft, observations made during radio-tracking, ground surveys of tracks or reports from rangers [e.g., (42–46)]. Additionally, most of the studies on contacts reported information regarding interactions, such as qualitative descriptions of observed encounters between dingoes and wild-living dogs, which is of limited value for disease spread modelling. Combined with the scarcity of studies, one third of the data of interest related to contacts was categorised as “low” relevance, and therefore was anecdotal or peripheral to the study's objectives. Also, these data were primarily historical, highlighting the urgent need for current research on these two topics. In contrast, studies on home range were more numerous and almost all provided detailed quantitative estimates. The majority of these studies included additional information on sex, age, season, and habitat. From the perspective of disease modelling, such information is relevant since the incorporation of a range of variables associated with home range into a model could more closely reflect reality. Many studies provided home range estimates based on GPS telemetry data [e.g., (25, 27, 47–50)], which represents valuable information for parameterisation of a disease spread model. Although research on dingoes and wild-living dogs' home range could be further expanded, most of the available data is recent and almost all studies provided “high relevance” data.

The large number of studies found in this review reporting indices of abundance and exploring dietary preferences was expected. Firstly, dingoes are viewed as an essential component of the Australian ecosystem by means of their trophic regulation (51). Secondly, they are considered a pest species because they predate livestock (52). Therefore, substantial efforts have been made to generate information on the impact of these predators on livestock, animal species or habitat as well as the broader ecosystem impacts of control programs through studies on the index of abundance and dietary preferences of dingoes and wild-living dogs.

Indices of abundance and dietary preferences are topics with potential application to disease spread modelling, although limitations need to be considered. For instance, the index of abundance could be used as a proxy for dingo and wild-living dog density. However, like studies we found on density, different methods (for example sand plot transects, spotlight counts, camera-traps) used to measure the index of abundance make it difficult to compare estimates across studies. The index might also vary due to other variables (such as animal movement patterns) which in turn can be affected by habitat, availability of resources and season (53). Regarding diet, the presence of preferred prey in an area could be used to infer the potential presence of dingoes or wild-living dogs. However, dingoes and wild-living dogs are capable of exploiting a large variety of prey species and prey selection can vary according to the quality and availability of resources (54, 55), thus causing the link between the presence of preferred prey and these predators to be dubious.

We identified that a third of the studies found in this review reported data of interest which was not separated by dingoes, feral dogs and hybrids (*n* = 11 mixed sample; *n* = 74 unknown sample). Reporting data separately as such would be valuable for disease spread modelling, although practically it may not always be feasible to evaluate dingo purity. In addition, we recognise there is ongoing debate about whether dingoes are a separate species to more recently introduced dogs (56). However, considering that some ecological and biological features might vary between dingoes, feral dogs and hybrids, the acquisition of separate data would allow the different types of dogs to be considered independently in disease spread modelling. Although a large amount of data of interest in the literature was attributed specifically to dingoes, this might not be an accurate representation of the populations investigated. Several studies used the term “dingo” to refer to a sample which could also contain hybrids, and to a lesser extent feral domestic dogs. These samples would have been more appropriately categorised as “unknown sample”; however, the decision was made to categorise the type of population studied according to the terminology used by the author. Studies reporting data of interest specifically on feral domestic dogs are scarce. This might be the reflection of a low occurrence (< 1%) of purely domestic dogs within the Australian wild-living dog population (22), rather than a gap in the literature.

The majority of studies assessed collected data of interest in rural or natural environments (defined as agricultural areas, countryside, parks, and forests), which reflects the fact that grazing, nature conservation, production forestry and cropping accounts for more than 95% of land use in Australia (57). However, the proximity of dingoes or wild-living dogs to urban areas, which was not studied extensively according to this review, has implications for potential disease transmission to humans and domestic animals (58, 59), and thus should be a focus of future research. In addition, more than one-third of studies assessed did not report lethal control efforts within the study location. This information is important because lethal control could affect dingo and wild-living dog ecology and behaviour. For example, in addition to directly reducing densities, lethal control can disrupt dingo and wild-living dog population stability leading to changes in population structure, movement patterns, territorial behavior, and other variables (60). This population instability can affect disease spread patterns by increasing the risk of transmission within populations, as previously suggested for European badger (*Meles meles*) populations (61, 62). In regards to rabies, reducing population density via culling programs has generally failed to control the spread of the disease in wildlife, highlighting the complexity of the relationships between host density, rabies transmission and potential antagonistic ecological or biological processes (21, 63).

Natural landscape barriers have implications in disease spread modelling by affecting the movement of animals. Such barriers have been incorporated into models of disease spread, including rabies models in North America (64, 65). No studies captured in this review provided data of interest in relation to natural landscape barriers. However, the dingo fence (which extends ~5,500 kilometers across Australia) acts as an anthropogenic barrier to dingo and wild-living dog movement, and could impede the spatial propagation of rabies into south-east Australia. Likewise, gestation period (time period between mating and birth) was another topic not captured in this review. This parameter is difficult to measure other than with captive animals in a controlled environment. All other topics of interest related to reproduction have been reported in a few studies, some of which were conducted in a captive environment (e.g., dingoes or dogs either captured from the wild or born in captivity from wild stock).

We found that a substantial amount of research on ecology and biology of dingoes and wild-living dogs has been conducted in central Australia and the east coast. Studies within these two regions did also report data on density, home range and contacts, potentially informing parameters for disease spread modelling. The lack of studies in south-eastern inland parts of Australia can be explained by a low abundance of dingoes south of the dingo fence. The northern Australian coast is of particular interest: Cape York Peninsula in northern Queensland (characterised by an equatorial climate) and the coast of Arnhem Land in the Northern Territory (characterised by both equatorial and tropical climate) have been identified as two possible entry points for rabies (21, 66, 67). Only a very limited number of studies were conducted in the tropical (*n* = 23) and equatorial (*n* = 2) climate zones, especially on density (*n* = 1 and nil), home range (*n* = 3 and nil) and contacts (nil studies), respectively. This highlights a major research gap in the literature and a need to generate further data on dingoes and wild-living dogs within these areas as it is directly relevant to Australia's preparedness for a rabies incursion.

This review—conducted comprehensively while balancing feasibility—has captured the vast majority of relevant literature. For example, the verification of our search strategy (performed to maximise sensitivity) only identified 6 additional records. This scoping review employed systematic, rigorous and transparent methods, based on a methodological framework (32). Consistent with this framework, the methodological quality of studies was not critically appraised, other than the relevance of data of interest based on the study objectives. While this may be perceived as a potential limitation, the approach did allow a large range of topics and study designs to be included. Well-researched areas of interest identified in this review (for example index of abundance and diet) could be prioritised for follow-up synthesis via a systematic review or meta-analysis, in which potential bias of the methodology and quality assessment of the data available would be considered with respect to disease spread modelling. Of particular interest, the home range studies captured in this review might provide sufficient quantitative data for meta-analysis, which would ultimately yield a pooled home range point estimate and variance parameters to use in disease modelling.

In conclusion, the noticeable expanding interest in dingo and wild-living dog research will most likely lead to improved knowledge in this field, which is promising for further work in disease spread modelling. We found a scarcity of quantitative estimates on parameters that are important for disease spread modelling, such as density and contacts. While considerable information has been generated from studies conducted in central Australia and the east coast, dingo and wild-living dog data which could be used to estimate parameter values for disease spread in northern Australia (where a rabies incursion is most likely to occur) is limited. Based on this review, further work should focus on these identified research gaps to strengthen our ability to develop reliable parameters for disease spread modelling, which would contribute to improving Australia's preparedness against a potential rabies incursion.

## Author Contributions

VG-R developed the protocols, forms, and guidelines of the review. VG-R, JA, BW, VB, and MW conducted the screening process and data extraction of level 1–3. VG-R, JA, and MW conducted the data extraction of level 4. TN screened the reference list for any missing relevant references. VG-R analysed the data and wrote the first draft of the manuscript. All authors provided feedback and participated in the development of the protocols, forms and guidelines, contributed to manuscript revision and approved the submitted version.

### Conflict of Interest Statement

BW was employed by company Big Sky Health Analytics. The remaining authors declare that the research was conducted in the absence of any commercial or financial relationships that could be construed as a potential conflict of interest.

## Supplementary Material

Appendix A Scoping review protocol.

Appendix B Forms and definitions.

Appendix C Guidelines.

Appendix D List of studies included, main extracted data and complementary studies reporting duplicated data.

Appendix E Quantitative estimates for the 3 key topics of interest, i.e., home range, density or population size and contact or interactions.

Data Sheet 2 Data extracted from Level 3, excluding location.

Data Sheet 3 Location data extracted from Level 3.
