# Supplementary material for: A Scoping Review of Dingo and Wild-Living Dog Ecology and Biology in Australia to Inform Parameterisation for Disease Spread Modelling
Source: Front Vet Sci. 2019 Mar 5;6:47. doi: 10.3389/fvets.2019.00047 (PMC6411765; doi:10.3389/fvets.2019.00047)
Supplement: Appendix A — Scoping review protocol. [file Data_Sheet_1.docx]

**Appendix A – Scoping review protocol**

Protocol for a scoping review on wild dog ecology and biology in Australia.

**Scoping Review Protocol**

**Research question**

The review will be guided following the research question: ‘What is the current state of knowledge of the ecology and biology of wild dogs, including dingoes, feral dogs and hybrids, in Australia?’

Populations of interest: Wild dogs, including feral dogs, dingoes and hybrids

Location: Australia

Topics of interest: Ecology and biology

Inclusion criteria: Primary research from any journal article, research report or data from Australian government agencies, conference proceeding/paper, theses/dissertation or book investigating/describing a topic of interest, peer-reviewed or not, related to a population of interest within the location of interest published in English. All years are considered.

**Search strategy**

The initial search will be conducted in four relevant electronic databases:

- Web of science core collection
- BIOSIS Previews
- CAB abstracts
- Zoological record
- SCOPUS

The terms used in the search are as follows:

Population: “Wild dog*” OR Dingo* OR “Feral dog*” OR “Canis lupus familiaris” OR “Canis lupus dingo” “Free roaming dog*” OR “Free-roaming dog*” OR “Free ranging dog*” OR “Free-ranging dog*” OR “Stray dog*” OR “Canis familiaris dingo” OR “Canis familiaris familiaris” OR “Wild-living dog” OR “Commensal dog” OR “Canis dingo” OR “Canis antarticus” OR “Canis familiaris australasiae” OR “Canis australiae” OR “Canis dingoides” OR “Canis macdonnellensis” OR “Canis familiaris” OR “Wild canid”

AND

Location: Australia*

AND

Topic: Ecolog* OR Biolog* OR Densit* OR Distribution* OR Population* OR Demograph* OR Habitat* OR Landscape* OR Diet* OR Water* OR Birth* OR Gestation OR Litter* OR Mating* OR Reproduct* OR Mortalit* OR “Life span” OR “Lifespan” OR “Life-span” OR “Contact rate” OR “Home range” OR “Home-range” OR Dispersal OR Movement* OR Age OR Vegetation OR Breeding* OR Dynamic* OR Interaction* OR Behavior* OR Sex OR Predat* OR Competition OR Trophic* OR Mesopredator* OR Activit* OR Genetic* OR Hybridisation OR Den* OR Breeding

A search on ‘Google’, applying the same algorithms, will be conducted to identify any relevant papers not found in the electronic databases. The first 100 search results from Google will be screened. Conference papers index via Proquest will be used to search for conference proceedings whereas Trove will be used to search for theses and dissertations. Australian government and research organization websites (CSIRO, Invasive animals CRC, Department of agriculture for each Australian state) will also be searched for new relevant papers or grey literature (any literature that has been unpublished or published without being controlled by commercial publishers). Additionally, emails will be sent to Australian government agencies, CSIRO and Invasive animals CRC to request any reports or unpublished material that might be relevant. Journal articles, research reports from government agencies, conference proceedings, theses and dissertations and books will be the only literature considered as eligible in the search.

A verification of our search strategy will be undertaken by screening the reference list of 15 relevant and recent documents on our topic of interest. Additionally, a wild dog expert will be asked to screen our list for any missing references.

References found by the search will be exported into a citation management software (Endnote). Duplicate versions of identical references will be eliminated. The final reference list will be uploaded to the DistillerSR (<https://distillercer.com/products/distillersr-systematic-review-software/>), which is a web-based electronic review platform specifically designed for the screening and data extraction of systematic reviews.

**Levels of screening and data extraction**

Two levels of screening will be undertaken to eliminate irrelevant articles, followed by a third level on data characterisation and data extraction:

- Level 1: First level relevance screening on abstract
- Level 2: Second level relevance screening on full-text article
- Level 3: Data characterisation and data extraction on full-text article

For each level, a screening or data extraction form will be developed in DistillerSR (Tables 1 and 2). Multiple reviewers (3-4) will pretest the forms using several selected articles, stratified by type of documents (Journal article, research report from Australian government agencies, conference proceeding, theses/dissertation) to ensure reproducibility and consistency of the method and evaluate reviewer’s agreement. If needed the forms will be modified to improve the structure of the forms or clarity of the questions. A level of agreement of 0.80 must be obtained between reviewers, using Cohen’s kappa statistic, before starting each level of screening or data extraction. All levels of the review will be conducted by two reviewers. Once the 3 levels of screening and data extraction are completed, data will be exported and cleaned in Excel. Analysis (descriptive statistics, meta-analysis, etc) will then be conducted.

**Appendix B - Forms and Definitions**

The three forms and a list of definitions used for a scoping review on wild dog ecology in Australia. Each form was performed by at least 2 reviewers per reference.

**Form – Level 1**

Table 1. Level 1 screening (*To perform on abstract)*

*Answer these questions using the abstract. If the abstract is not available, answer the following questions, in a sensitive manner, using the title. Only exclude titles which are clearly irrelevant. If there is any possibility that they might be relevant, select ‘unable to determine’ which will send the citation to the next level.*

| **Question** | **Options** |
| --- | --- |
| 1. Does the work reported in this citation investigate some aspect of the broad research question, i.e. what is the ecology and biology of wild dogs (including dingoes, feral dogs and dingo-dog hybrids) in Australia?  **See appendix I for definitions* | □ Yes  □ No. Please specify and check all that apply:  □ The research is not related to either wild dogs (dingoes, feral dogs, dingo-dog hybrids). Please specify which animal population is investigated. – EXCLUDE  □ The data was not collected in Australia. Please specify which country is investigated. – EXCLUDE  □ The research does not provide information on a topic of interest (i.e. ecology or biology) – EXCLUDE  □ Unable to determine – paper will be procured |
| 2. Is the research in English? | □ Yes, in English  □ No, research in foreign language  □ Unable to determine – paper will be procured |

**Form – Level 2**

Table 2. Level 2 screening and categorisation (*To perform on full-text article)*

| **Question** | **Options** |
| --- | --- |
| 1. Is the text in English? | □ YES  □ NO – EXCLUDE. End of form level 2 |
| 2. Is the full-text available | □ YES  □ NO – EXCLUDE. End of form level 2 |
| 3. What type of document is this?  **Publication does not need to be peer reviewed* | □ Scientific journal article  □ Conference proceeding/paper  □ Thesis or dissertation  □ Governmental (Including report, journal, data or website)  □ Book or book chapter  □ Other, Please specify – EXCLUDE. End of form level 2 |
| 4. Does this work provide useful information, originating from a primary research, on the ecology or biology of wild dogs in Australia, in relation to one or more of the topics of interest (population characteristics, environmental requirements, reproduction, life cycle, group dynamics, group structure, behavior and genetics (hybridisation))?  **Please refer to list of topics of interest*  ***Useful information = Data that can be used to derive wild dog population distributions in Australia*  ****A primary research would include any original research reporting results from field samples or data collected from the investigators. For the purpose of this review, we do not consider systematic review, meta-analyses or modelling studies as primary research.* | □ Yes, definitively – Include  □ Yes, but partial information only – Include  □ No. Please specify and check all that apply:  □ The research does not provide information related to any of the animal populations of interest (i.e. dingoes, feral dogs, dingo-dog hybrids). Please specify which animal population is investigated – EXCLUDE. End of form level 2  □ The data was not collected in Australia. Please specify which country is investigated – EXCLUDE. End of form level 2  □ The research does not provide useful information on the ecology or biology of wild dogs in Australia, in relation to the topics of interest– EXCLUDE. End of form level 2  □ The document does provide some useful information on the ecology or biology of wild dogs in Australia, in relation to the topics of interest, but this information does not originate from a primary research (Not original data) – EXCLUDE. End of form level 2 |

**Form –Level 3**

Table 3. Level 3 data extraction (*To perform on full-text article)*

| **Question** | **Options** |
| --- | --- |
| 1. a) This research provides information on which topic of interest in wild dogs (feral dogs, dingoes or dingo-dog hybrids)? Please check all that apply. | □ Population characteristics  □ Density/population size estimates  □ Index of activity/density/abundance  □ Quantitative observations of live wild dogs  □ Environmental requirements  □ Habitat/climate/landscape preference (including pattern of density, movement or activity)  □ Landscape barriers  □ Diet  □ Water requirements  □ Reproduction  □ Birth rate  □ Litter size  □ Litter sex ratio  □ Reproductive age  □ Gestation time  □ Birth period  □ Mating period  □ Life cycle  □ Mortality rate  □ Life span  □ Group dynamics and behavior  □ Contact rate/interactions  □ Home range  □ Dispersal/migration  □ Regular movement pattern  □ Long distance movement outside home range  □ Age of independence  □ Daily patterns of movement or activity  □ Monthly or seasonal patterns of density, movement or activity  □ Group structure  □ Group size  □ Proportion of male/female  □ Proportion by age category  □ Genetics  □ Hybridisation (Percentage of hybridisation in a population)  □ None of the above ̶ Exclude |
| 1. b) (Sub-question, for each topic of interest chosen) | □ The information provided is directly linked to the objective(s) of the study  □ Other |
| 2. Which animal population is studied in this research, in relation to the data selected in Question 1? Please check all that apply. | □ Feral dogs _______________  □ Dingoes _______________  □ Dingo-dog hybrids ____________________  □ Mixed sample ____________________  □ Unknown wild dog ____________________ |
| 3. a) Does the document provide information on the location of the data collected and selected in Q1? | □ Yes  □ Information on location provided in other paper _____________  □ Not reported |
| 3. b) The data from Q1 was collected in which location in Australia? Please specify all information provided.  *(** Be as precise as possible as this data could potentially be used to pinpoint the locations of research on a map of Australia)* | □ State ___________  □ Region _____________  □ Park _____________  □ Nearest city, town ___________  □ Inside city, town ___________  □ GPS coordinates ______________  □ Other information, Please specify ____________________________ |
| 4. The data from Q1 was collected in which type of area, in terms of wild dog control (which includes baiting, trapping or shooting)? | □ Area(s) with wild dog control during time period of study  □ Area(s) without wild dog control during time period of study  □ Both types of areas  □ Entire state/country investigated  □ Not applicable  □ Not reported |
| 5. The data from Q1 was collected in which type of environment? | □ Peri-urban/Urban  □ Rural/agricultural/Natural environment  □ Captive environment (captive dingoes, wild dogs)  □ Mine environment (mine sites)  □ Entire state/country investigated  □ Not reported |
| 6. The data from Q1 was collected during which year(s)? | □ Year(s): ____________________________  □ Not reported |
| 7. The paper from Q1 was published in which year? | □ Year: ____________________________  □ Not reported |

**Definitions**

Biology: A branch of knowledge concerned with life, including origin, morphology, physiology, behavior, growth, evolution and distribution of living organisms

Ecology: A branch of knowledge concerned with the relationships among living organisms and their environment

Wild dog: Any pure-bred dingo (Canis lupus dingo), dingo-dog hybrid (Canis lupus dingo X *Canis lupus familiaris*), or feral domestic dog (*Canis lupus familiaris*) that lives in the wild and does not depend on humans to survive.

Feral dog (*Canis lupus familiaris*): Domestic-type dog that has escaped from domestication and lives in the wild apart from humans.

Dingo (Canis lupus dingo): Wild dog found in Australia that descended from Asia.

Stray dog (*Canis lupus familiaris*): Domestic dog that is owned by humans but is free to roam out in the wild (free-roaming)

Density: Number of individuals per unit area

Population size: Number of individuals within one population

Index of activity/abundance/density: Estimates of relative species abundance/activity (derived from sand plots, spotlights, hair traps, camera-traps or scat density)

Observations of live dingoes: Quantitative observations of live wild dogs within a defined territory and time period

Habitat/landscape preference: Environment where the animal is usually found

Landscape barriers: Landscape features or habitat types that may block movement of animals

Diet: Various types of food that the animal eats

Water requirements: The quantity of water needed for the animal to survive

Birth rate: Probability to successfully mate and produce a litter

Litter size: Number of live young animals born following one gestation

Litter sex ratio: Proportion of male vs female in each litter

Reproductive age: Age at which the animal can reproduce

Gestation time: Length of time of the gestation period in females

Birth period: Period in the year when the females give birth

Mating period: Period in the year when the animals pair for reproduction

Mortality rate: Number of deaths per population size over a specified period of time

Life span: Duration of time that an animal lives

Contact rate: Number of interactions between 2 individuals per unit time

Contacts/interaction: Any type of descriptions of intra-specific contact rates or interactions between packs or individuals, including indices of probability of contact (Sightings of groups of animals traveling together are considered as a contact/interaction only if it is mentioned as such by the authors)

Home range: Main area of activity of an individual

Dispersal/migration: Movement of juveniles or adults away from their area of origin or main home range to settle in a new location (Includes natal or adult dispersal/migration).

Regular movement patterns: Movement within the main home range of the animal, during the course of its regular activities

Long distance movement outside home range: Movement travelling outside home range before returning to home range.

Seasonal activity: Does not include behavior or interactions

Temporal activity: Does not include behavior or interactions

Age of independence: Age at which a young animal becomes independent of his mother

Group size: Number of individuals in a group or a pack

Proportion of male/female: Proportion of male vs female in a group or a pack

Proportion by age category: Age distribution within a group or a pack

Hybridisation: Percentage of hybridisation within a wild dog population

Peri-urban: Area surrounding a city

Rural/agricultural: Area on the countryside (usually low population density). May or may not include land devoted to agriculture.

Natural environment: Wild, natural area, which has not been significantly modified by human activity (wildland), which includes parks, forest, beaches, etc.

Primary research: Original research reporting results from field samples or data collected from the investigators. For the purpose of this review, we do not consider systematic review, meta-analyses or modelling studies as primary research, unless they provide original data collected from the field.

Systematic review: Literature review that collects and synthesizes data from multiple studies to answer a specific research question

Meta-analyses: Quantitative statistical study that combines and analyses data collected from multiple studies to provide a single conclusion (Can be part of the systematic review)

Modelling study: Study that uses computerised mathematical simulations to represent and explain real-world systems or phenomena.

Useful information: Data that can be used to derive wild dog population distributions in Australia

**Appendix C – Guidelines**

Guidelines used by all reviewers to answer questions from all three levels during the scoping review on wild dog ecology in Australia.

**Guidelines – Level 1**

Q1. Ecology/biology of wild dogs

1) If the abstract reports some type of information/observation/data on the ecology or biology of wild dogs 🡪 YES

For example:

- Study on the ecology, distribution, behaviour, or breeding of dingoes
- Study on another animal species but provides some information on wild dogs ecology or biology

2) If, based on the abstract, the study investigates or might investigate some aspect of wild dogs either directly or indirectly and the abstract does not report ecological or biological data on wild dog populations but it is a possibility that this might be found in the full-text article 🡪 MAYBE

For example:

- Abstract does not provide full information on the animal species studied or the data that is reported
- Study focuses on disease in wild dogs but does not mention what type of research/observations were undertaken
- Study focuses on control/management of dingo and other wild dog populations in Australia and does not provide any further information on the content of the paper
- Study investigates areal baiting in wild dogs or any other animal species and does not provide full information on the data reported
- Laboratory-based study investigates wild dogs on a topic that is not related to one of our topics of interest, but there is a possibility that the sample collection might provide information on wild dog populations

3) If, based on the abstract, the study investigates or might investigate some aspect of wild dogs either directly or indirectly and the abstract does not report ecological or biological data on wild dog populations and this is NOT a possibility in the study 🡪 NO

- Clinical cases in one or more dingoes
- Laboratory-based study investigates wild dogs on a topic that is not related to one of our topics of interest and without any relevant sample collection

4) If, based on the abstract, the study does not seem to investigate any aspects of wild dogs neither directly nor indirectly 🡪 NO

- Study is mainly focused on another animal species but the abstract mentions the word “dog” only to explain the context of the problem. There is no further information provided which could indicate that the study might investigate wild dogs.
- Study provides information on domestic dogs (dogs that have owners)

5) If the abstract is not available, only exclude titles which are clearly irrelevant. If there is any possibility that the study might be relevant 🡪 MAYBE

Additional notes

**Study that does not provide original data**: In Level 1, we are not evaluating the type of study (primary research or not). All articles that are not ‘primary research’ will be excluded in Level 2. It is therefore important, for Level 1, to only evaluate if the paper might provide information on the ecology of wild dogs in Australia and, at this point, not exclude a citation based on the type of study (for example book, review, report, meta-analyses, etc).

**Study that investigates stray dogs**: Our population of interest is Wild dogs, which we defined as “Any pure-bred dingo (*Canis dingo*), dingo-dog hybrid (*Canis dingo X Canis familiaris*), or feral domestic dog (*Canis familiaris*) that lives more or less in the wild, apart from humans, where its population can be maintained through many generations (naturalised population). The dog is not subject to any type of physical restrictions by humans. Also, humans do not intentionally provide any needs essential for the dog’s survival and well-being. The dog could, to a certain extent, rely on human resources, such as feeding in dump areas, as long as it remains unintentional provisions.”

The term ‘stray dog’ is usually used in an urban context to define a “Domestic dog that is currently owned by humans but is unrestrained or that has recently escaped, been released or is lost and can therefore roam in urban areas or the wild. This dog has a strong attachment to humans as it is or was, until recently, the domestic pet of humans.” Following this definition, stray dogs are not included in our population of interest. Therefore, we will exclude stray dogs in the review.

**Guidelines – Level 2**

Q3. Type of documents

- Scientific journal articles: These must be considered as scientific journals. Entertainment or popularization documents, such as magazines or media articles, are not included in the review.
- Governmental: This includes any document or information produced by the government, such as governmental reports, journals, data or websites. Therefore, when a journal is published by the government, the document would be classified as ‘Governmental’.

Q4. ‘Partial information’

- When answering Q4, only consider the type of data provided. Do not consider the methodology used to obtain this data.
- ‘Partial information’ represents a ‘Yes’ but not as strong as a ‘Yes, definitively’.
- Studies reporting the index of abundance in a given area would be categorised as ‘Partial information’, unless additional information is provided which could make it possible to compare population densities between seasons, habitat types or climatic conditions (in which case this would become ‘Yes, definitively’). Please add ‘Index of Abundance’ in the text box.
- Studies reporting quantitative data on observations of wild dogs, within a territory or time period which is not well defined, for a purpose other than to investigate population characteristics (for example control of dingoes) would be categorized as ‘Partial’. Please add ‘Quantitative observations’ in the text box.

Q4. Exclusions

Not wild dogs:

- This option is chosen when there is no information at all provided on wild dogs.
- Studies that do not specify if they are investigating pet dogs or wild dogs are excluded.

Not useful information of interest:

- When using this option, I would suggest writing a few words in the text box on the specific reason for exclusion
- Studies only reporting the presence/absence of wild dogs in a specific area, without any information on population characteristics (wild dog densities, sex or age distribution) and/or association between presence of wild dogs and environmental characteristics, are excluded.
- Studies only reporting a sample of dingoes, with or without sex or age distribution, captured for a purpose other than to investigate population characteristics (for example disease study, control of dingoes, etc) are excluded.
- Studies only reporting number of livestock killed by wild dogs are excluded.
- Studies reporting only the ingestion of a few preys, without any information on the relative importance of the various types of food eaten by wild dogs (such as the information provided by scat analyses), are excluded.
- Case studies on single wild dogs are excluded.
- Studies only reporting information on the role of the dingo as a top predator in the ecosystem or the effect of the dingo on other animal species (mesopredator release, trophic cascade, etc) without providing information on any of the topics of interest are excluded.
- Government reports providing an overview of ongoing projects without reporting specific data or results from these projects are excluded.
- Studies only reporting location of wild dog baiting areas are excluded.

Not primary

- A primary research would include any original research reporting results from field samples or data collected from the investigators. For the purpose of this review, we do not consider systematic review, meta-analyses or modelling studies as primary research.
- A paper that reports relevant data cited as ‘Personal communication’ would be considered as ‘Not primary’ data since it does not originate from the author. Please add ‘Personnal communication’ in the text box.

**Guidelines – Level 3**

In the form, Q1 is related to the topics of interest (parameters of wild dogs). Q2-Q6 are related to the location/environment/dog population (dingo, feral, etc)/year of data collection in relation to the parameters selected in Q1. One study might provide data on Density and on Habitat (selected in Q1). In most of the cases, the answers for the following questions on location/environment/population/year of this data (Q2-Q6) will be identical for both Density and Habitat. However, it might happen, especially in the case of a Thesis, that one parameter in a paper (for example Density) was not collected in the same context (location/environment/population/year) as another parameter from this same study (for example Habitat). Therefore, Density and Habitat might have different answers to one or many of the following questions (Q2-Q6).

• Please, chose all topics of interest which were collected (year, animal population, location, etc) together. The following questions Q2-Q6 should therefore relate to the topics of interest selected in Q1. If the document contains additional topics of interest which were collected in a different setting (year, animal population, location), please select ‘This form, New Instance – This reference’. This will submit your current form and open a new identical form, for this same reference. You can then complete this form for the additional topics of interest. If you wish to submit your form and move on to the next reference, please select ‘This form – Next reference’.

• If one study reports data on a topic of interest (for example home range) from multiple years, each year comprising a different location, please create only one instance.

• Always create a new instance for each thesis chapter containing relevant data, even if the data was collected in the same study area and same year as another chapter (In other words, consider a chapter as an individual study). For each instance, please write in the last question (Q8) the appropriate chapter. The same applies for different conference papers found within a conference proceeding.

**Q1 – Year of publication**

• Please answer this question using the year of publication provided directly in the paper, if available. Do not use the year from Distiller. (This year originates from endnote and I have noticed some mistakes).

**Q2 a) Topics of interest**

• Please select the appropriate ‘Topic of interest’ in the list of Q1 for any information reported, regardless of if this information is limited, qualitative or anecdotal.

• Index of activity/abundance/density is usually calculated using sand plot, spotlight, hair trap, camera-trap or scat density. It is an estimate of relative species abundance/activity. It is not an actual estimate of the density/population of wild dogs.

• If a study reports an index of activity across time (daily or monthly), please select both options ‘Index of abundance’ and ‘Group dynamics and behaviors – dialy pattern of activity’ (for daily activity) or ‘Group dynamics and behaviors – monthly or seasonal pattern of activity’ (for monthly or seasonal activity).

• Social stability index (such as scent marking, howling activity, etc) has a communicative function and is an indicator of territoriality. This is not part of the topics of interest.

• Scat collection rate should be categorised as index of activity (some studies consider this measure as an index of social stability, others as an index of abundance). Please indicate ‘Scat collection rate’ in the text box of the last question Q8.

• Please select quantitative observations on live wild dogs if the document reports a number of live wild dogs recorded within a defined territory and time period.

• ‘Dispersal/migration’ should be selected only when there is indication that the movement reported corresponds to dispersal (adult or natal) or migration outside the main home range (see definitions in section 3 of this document). 'Home range' should be selected when the study clearly investigates the home range of the animals.

• Proximity to water (or any associations related to water bodies) should be considered as ‘habitat preference’. ‘Water requirements’ should be selected if the paper provides data in relation to the quantity or frequency of water required for the survival of wild dogs.

• Data collected within baiting areas should not be excluded since human-induced control of dingoes plays a 'role' in their ecology.

• Data related to attacks of wild dogs on livestock, wild dog bounties, or number of wild dogs killed are not part of the topics of interest, unless an index or estimate of abundance/density is calculated.

• Sex or age distribution from a sample of wild dogs would be included if the study investigates population characteristics of wild dogs (This would not include, for example, disease study, control of dingoes, etc).

• Please select ‘None of the above’ if the paper provides information that is not exactly in relation to the topics of interest. This paper will be excluded. The following questions do not apply. Please simply answer ‘not reported’ for Q3-7 (An answer needs to be given for each question as they are mandatory in order to submit the form).

**Q2 b) Information in relation to objectives or other**

• The answer ‘The information provided is directly linked to the objective(s) of the study’ includes secondary objectives.

• The answer ‘other’ also includes limited and anecdotal information.

• The decision for this question should not be based on the quality of the methods (including sample size). Quality assessment will not be undertaken for this review.

**Q3 – Animal population studied (Dingo/Feral dog/Dingo-dog hybrid/Unknown)**

• Please select the term used in the paper. Some studies might mention sampling 'dingoes', although an effort does not seem to have been made to truly distinguish Dingoes from Dingo-dog hybrids or Feral wild dogs (through DNA testing, morphological characteristics or other tests). Given that it is difficult to distinguish hybrids from dingoes, and that the validity of the genetic purity test is debated, we should report what is written by the author.

• Please select all terms mentioned in the paper (For example, if a paper mentions dingoes and feral dogs, please select both options)

• If a paper mentions that they investigated wild dogs, without distinguishing between the different populations (Dingo, hybrid or, feral dog), please select ‘Unknown wild dog’. If a paper uses the definition of Dingo (Canis dingo or hybrids) but does not investigate hybridization in the sample, please select once again ‘Unknown wild dog’. If a paper states that the sample studied comprises a mixture of pure dingoes, hybrids and/or feral domestic dogs (which was verified in one way or another, according to the authors), but reports the data in an aggregated form (does not report the data for each population group), please select ‘Mixed sample’. Finally, if a study provides information on a parameter for each population group separately, please select the appropriate type of dog category (Dingo and/or Hybrid and/or Feral dog).

• If more than one dog category has been selected in this question, please enter, in the text box, the parameters that are linked to each dog category selected.

• Example: A study reports the % of hybridization in the sample studied (10% dingoes, 60% hybrids and 30% feral), reports the density for the entire sample, and the dispersal distance travelled for 2 individual dingoes and 2 feral dogs of this sample. This same study also reports the index of activity of another wild dog sample, from which we do not know the composition (Dingo, hybrid or feral). For this example, providing that the location/year/environment are all similar for these 4 parameters (if not, a new instance will need to be created), the answers would be the following:

□ Feral dogs _______Hybridization, Dispersal________

□ Dingoes ______ Hybridization, Dispersal_________

□ Dingo-dog hybrids __________Hybridization__________

□ Mixed sample _______Density_____________

□ Unknown wild dog ________Index____________

**Q4 – Location**

• A study might have been conducted in more than one study area. If so, please provide information from one location, and click ‘Yes’ to the question ‘Is there another location?’, and so on.

• Always enter a new location for each GPS coordinate that is provided.

• You do not need to enter multiple locations (Location 1, location 2, etc) if the study is conducted in one general study area (state, region, etc), with multiple smaller study locations inside this area (for example station). All information can be entered directly in Location 1. This applies also for a single GPS coordinate, which corresponds to the center point around multiple smaller locations. Please enter the names of these multiple smaller locations, if provided in the paper, in the appropriate textbox (station, city, other, etc).

• You can press ALT+248 to enter the degree (°) symbol for GPS coordinates.

• Only provide information that is reported in the paper (do not answer using your own personal knowledge of Australia’s geography). Please enter all information reported related to the state, region, station, park, city, GPS coordinates, etc.

• Please record information from the first location mentioned in the paper (Location 1) and then go on with the following locations using the order of appearance in the paper. Resolving conflicts will be a lot easier this way.

• To avoid conflicts, please enter the GPS coordinates using the form

Lat: -22°22’ / Long: 122°22’ (rather than S/E, degrees, spaces, etc)

• When important information on location is provided in another paper, please check the option ‘Information on location provided in other paper’. I will provide the appropriate papers for completing the information on location for these studies (I will try to provide the papers in advance).

• If a new instance (i.e. a new form) is created for a new parameter which contains the exact same location(s) as the previous parameter, you can enter ‘Same as previous instance’ in the ‘Other information’ text box of Q4b. This will help avoid re-entering the same data multiple times.

**Q5 - Control**

• If it is stated that control of wild dogs is very limited or irregular, please select the option ‘Area(s) without wild dog control or limited control in space or time’.

• Control measures include baiting, shooting, trapping. The dingo fence is not included in these measures as we can distinguish these areas geographically on a map. Please write ‘Dingo fence’ in the text box of Q8 if the study compares 2 study areas on each side of the dingo fence (it would be clearly stated in the methods).

• If the study period interchanges between control and no-control, please chose ‘with control’.

**Q6 - Natural/Rural/Agricultural and Peri-Urban/Urban**

• Please select ‘Peri-urban/Urban environment’ if the paper clearly describes the study area as being peri-urban. Please select ‘Natural/Rural/Agricultural environment’ if the study area is located away from the city. Please select ‘Captive environment’ if dogs were kept in captivity (zoo, or other). Please select ‘Mine environment’ if one of the study area was within a mine site. Please select ‘Entire state/country’ if an entire state or country is investigated (for example a survey conducted in each district from the entire state of NSW).

**Q7 – Year of collection**

• Use - for a range of years (Example ‘2012 - 2014’ for ‘2012, 2013 and 2014’). Use comma (,) for separated years (Example ‘2012, 2014’ for ‘2012 and 2014’).

**Guidelines - Level 4**

Home range

General guidelines for data extraction

- Whenever possible, always report the data from all animals combined together from each study in an aggregated form (Therefore, do not separate by regions, sites, sex, age, seasons, etc).
- If a study provides multiple estimates using different methods of calculation (Minimum Convex Polygon 95%, Minimum Convex Polygon 80%, Adaptive Kernel 95%, Fixed Kernel 50%, etc), please report the estimates for each type of method separately.

Guidelines per column

Method data collection

- Describe the methods used for data collection to evaluate home range. Chose between:
  - GPS Satellite tracking
  - Radio-tracking with ground or aircraft searches
- The following abbreviations will be used:
  - **GPS tr.** = GPS Satellite tracking
  - **Radio-tr.** = Radio-tracking with ground or aircraft searches
- Please write **NR** (Not reported) when no methods are reported.

Method data calculation

- When quantitative estimates are reported, please indicate the method used for home range calculation (For example Minimum Convex Polygon 95%, Minimum Convex Polygon 80%, Adaptive Kernel 95%, Fixed Kernel 50%, Movement-based Kernel Density Estimates 85%, etc).
- The following abbreviations will be used:
  - Minimum Convex Polygon 95% = **MCP_95**
  - Adaptive Kernel 95% = **AK_95**
  - Fixed Kernel 95% = **FK_95**
  - Movement-based Kernel Density 95% = **MBKD_95**
  - Minimum Convex Polygon 80% = **MCP_80**
  - And so on, for each level of percentage…
- If a study provides multiple estimates using different methods, please add lines for each additional method in the excel file and fill in the data accordingly (See example in Excel file, spreadsheet ‘Examples’, RefID 77).
- Please write NR (Not reported) when no methods are reported.

Specification on data

- We will only report quantitative estimates for the entire sample of the study (Not subgroups like males vs females, adults vs young, seasons, etc). One exception: In the rare case of a study which only provides estimates in an aggregated form for different groups (for example male adult vs female young, etc) but does not provide data for the entire sample (mean, median or range), or individual data for each dingo, we will report the estimates for each group. Please add lines in the excel file for each ‘group’ and fill in the data accordingly.
- If the paper reports ‘Pack territory’ or ‘Pack home range size’, please add the term ‘**Pack area’** in this column and fill in the data accordingly.

n

- Please provide the sample size for the quantitative estimates reported (Mean, median and range).
- A few studies have excluded the data from specific individuals in the final results based on a particular criterion (such as failure of the collar, not reaching an asymptote, etc). These criteria can vary from one study to another. Therefore, the sample size for home range estimates might be smaller than the number of animals trapped and collared.
- The sample size unit is number of animals. In the occasional cases of estimates on Pack territory, the unit is number of packs.

Mean estimate ± SE (km^2^)

- If a mean estimate for the entire sample is specified in the document (with or without SE), please indicate the mean estimate in this column, using the unit km^2^.
- Although most studies provide a table containing results for each individual dingo and a mean estimate could be calculated using this data, we will only indicate the mean estimate if this has been calculated and reported by the authors (in the text, abstract or tables).
- Please write NR (Not reported) when no mean estimate is specified.

Median (km^2^)

- Please indicate the median estimate in this column, using the unit km^2^.
- Since the majority of studies provide a table containing data for each individual dingo, the median of the sample can be found using this table, most of the time.
- Please write NR (Not reported) when no quantitative estimates are reported.

Range (km^2^)

- Please provide the minimum and maximum in this column, using the unit km^2^.
- Since the majority of studies provide a table containing data for each individual dingo, the minimum and maximum values of the sample can be found using this table, most of the time.
- Please write NR (Not reported) when no quantitative estimates (range) are reported.

Density

General guidelines for data extraction

- Whenever available, always report the quantitative estimates of density or population size (mean estimate and/or range) in an aggregated form (including all sex/age, regions combined, etc).
- If a study provides multiple density or population size estimates in different types of environments which are not comparable one to another in a density or population size perspective, such as baited vs unbaited sites, pre-baiting vs post-baiting, or inside dingo fence vs outside dingo fence, please report the estimates for each type of environment separately (see guidelines ‘Specification on estimate’). Baited and unbaited sites are always presented separately, even if baited sites also contain estimates from a pre-baiting time period.
- If a study provides multiple density or population size estimates from different regions (including maps) or sites (site 1, site 2, site 3, etc) which are comparable one to another in a density or population size perspective, please combine these estimates by reporting the minimum and maximum values (see guidelines ‘range’).
- If a study provides multiple density or population size estimates from different time periods (including graphs), please combine these estimates by reporting the minimum and maximum values (see guidelines ‘range’). If this time period includes periods with and without baiting which are not clearly separated, please indicate ‘Includes periods with and without baiting’ in the column ‘Specification’ (see guidelines ‘Specification on estimate’).
- If a study reports multiple density or population size estimates derived from different methods of calculation, please provide the data for the most accurate estimate according to the criteria given in the study (For example AIC). If the study does not report which estimate is the most accurate, provide estimates from all methods separately (see guidelines ‘Method data collection’ and ‘Method data calculation’).

Guidelines per column

Density or Population size

- Chose *Density* only if the paper reports a density estimate. If a paper reports a population size, with or without the area of the study site, chose *Population size* and report the estimates accordingly (we will not make calculations). If a paper reports both density and population size estimates, please chose *Density* and report the density estimates accordingly.

Method of data collection

- Provide a brief description of the methods used to collect data for density of population size estimation (For example radio-tracking, GPS tracking, interviews, aerial surveys, ground surveys for tracks, etc).
- If the study reports estimates from different methods and does not report which density or population size estimate is the most accurate, please add additional lines in the excel file and fill in accordingly (one method per line)
- Please write NR (Not reported) when no methods are reported.

Method of data calculation

- When quantitative density or population size estimates are reported, please indicate a brief description of the methods used for calculation (for example number of dogs sighted, capture-mark-recapture, etc).
- If the study reports density or population size estimates from different methods and does not report which estimate is the most accurate, please add additional lines in the excel file and fill in accordingly.
- Please write NR (Not reported) when no methods are reported.

Specification on estimate

- If applicable, provide specifications in relation to the quantitative density or population size estimates reported: Pre-baiting vs post-baiting, Baited sites vs unbaited sites, Inside dingo fence vs outside dingo fence or ‘Includes periods with and without baiting’.
- If a study provides multiple density or population size estimates in different types of environments which are not comparable one to another in a density or population size perspective, please add additional lines in the excel file and fill in accordingly.
- Please add ‘Minimum known density’ to the description when the calculation only provides a minimum known density.

Estimate (95% CI)

- If an overall density or population size estimate is provided for the study (with or without 95% CI), please indicate the estimate in this column, using the unit dogs/km^2^ for density and dogs for population size.
- Please write NR (Not reported) when no quantitative estimates are reported.
- If more than one estimate is reported, please write NR (Not reported) and refer to section ‘Range’ below.

Range

- If density or population size estimates are reported for subpopulations please provide the minimum and maximum in this column, using the unit dogs/km^2^ for density and dogs for population size.
- If a study reports density or population size estimates for multiple sites (for example site 1, site 2, site 3, etc) which are comparable or if the density or population size estimates are reported in a graph or a map, please combine these estimates in one line by indicating in this column the minimum and maximum values. Please use the ~ symbol if the estimate is an approximation (usually used in the case of maps or graphs).
- Please write NR (Not reported) when no quantitative estimates (range) are reported.

Contacts

General guidelines for data extraction

- Contacts and interactions are defined as any type of descriptions of intra-specific contact rates or interactions between packs or individuals. Indices of probability of contact (e.g home range overlap) were classified as a contact/interaction topic. When a paper only provides information on sightings of groups of animals traveling together, data was considered as a contact/interaction topic if it was mentioned as such by the authors. Therefore, information on groups of animals travelling together without further discussion about interactions was not classified as a contact/interaction topic.
- If a study reports multiple estimates for contact using different methods of calculation (for example UDOI and PHR), please provide estimates for each method. For all other cases, report the quantitative point estimates in an aggregated form (including all sex/age, regions, combined, etc) whenever available.

Guidelines per column

Method of data collection

- Provide a brief description of the methods of data collection used to evaluate contacts (for example: Observations of activities from aircraft using radio-tracking, Camera traps, GPS tracking, etc).
- Please write NR (Not reported) when no methods of data collection are reported.
- Parameter and method for contact or interaction measurement When quantitative estimates are reported, please indicate the methods for data calculation of contacts (for example Utilisation Distribution Index Overlap (UDOI), Probability home range overlap index (PHR), etc).
- If a study reports multiple estimates using different methods of calculation (for example Utilisation Distribution Index Overlap and Probability home range overlap index), please add a line in the excel file and fill in the data accordingly.
- Please write NR (Not reported) when no quantitative estimates are reported.
- Only quantitative estimates (mean or range) are reported in the following columns. Descriptive or qualitative information are not reported.

Specification on estimate

- If applicable, provide specifications in relation to the quantitative density or population size estimates reported (For example, type of environment)

Estimate ± SE or SD

- If an overall estimate is provided for the study (with or without SE or SD), please indicate the estimate in this column.
- Please write NR (Not reported) when no quantitative estimates are reported.
- If more than one estimate is reported, please write NR (Not reported) and refer to section ‘Range’ below.

Range

- If estimates are reported separately for different subpopulations, please provide the minimum and maximum in this column.
- If a study reports estimates for multiple regions or sites (for example site 1, site 2 site 3, etc) or time periods (including graphs), please combine these estimates in one line by indicating in this column the minimum and maximum values. Please use the ~ symbol if the estimate is an approximation.
- Please write NR (Not reported) when no quantitative estimates (range) are reported.

**Appendix D – List of studies, duplicates and extracted data**

List of studies included in the scoping review on wild dog ecology in Australia, data extracted and complementary studies reporting duplicated data.

| Refid | Reference | Topics of interest investigated | Relevance of data | Type of population sampled | Presence of lethal control | Type of environment | Duplicated data |
| --- | --- | --- | --- | --- | --- | --- | --- |
| 17 | (Bannister et al., 2016) | Index | High | Dingo | Areas without control | Rural/Natural |  |
| 18 | (Behrendorff and Allen, 2016) | Litter size, Birth period, Mating period, Life span, Group size | High, Low | Unknown wild dog sample | Type of control not reported | Rural/Natural |  |
| 26 | (Stephens et al., 2015) | Hybridisation | High | Dingo, Hybrid | Entire state | Entire state |  |
| 39 | (Davis et al., 2015) | Diet | High | Unknown wild dog sample | Entire state | Entire state |  |
| 40 | (Gordon et al., 2015) | Index | High | Dingo | Areas with and without control | Rural/Natural |  |
| 51 | (Leahy et al., 2015) | Index | High | Dingo | Type of control not reported | Rural/Natural |  |
| 56 | (Frank et al., 2014) | Index, Seasonal activity, Group size | High, Low | Dingo | Areas without control | Rural/Natural |  |
| 57 | (Spencer et al., 2014) | Diet | High | Dingo | Type of control not reported | Rural/Natural |  |
| 60 | (Allen and Leung, 2014) | Diet | High | Unknown wild dog sample | Areas with and without control | Rural/Natural |  |
| 63 | (Greenville et al., 2014) | Index, Temporal activity, Seasonal activity | High | Dingo | Type of control not reported | Rural/Natural |  |
| 65 | (Forsyth et al., 2014) | Index, Habitat, Temporal activity, Seasonal activity | High | Unknown wild dog sample | Areas without control | Rural/Natural |  |
| 68 | (Newsome et al., 2014a) | Diet | High | Dingo | Type of control not reported | Rural/Natural, Mine |  |
| 75 | (Allen et al., 2013b) | Habitat, Home range, Temporal activity, Proportion sex, Proportion age | High, Low | Dingo | Type of control not reported | Urban | (Allen, 2006a)  (Allen, 2006b)  (Allen, 2007) |
| 77 | (Newsome et al., 2013b) | Habitat, Contact, Home range, Movement, Long distance, Temporal activity, Group size, Proportion sex | High, Low | Dingo | Type of control not reported | Rural/Natural, Mine |  |
| 78 | (Allen et al., 2013a) | Index, Seasonal activity | High | Dingo | Areas with and without control | Rural/Natural |  |
| 80 | (Appleby et al., 2013) | Litter size, Birth period | Low | Dingo | Type of control not reported | Rural/Natural |  |
| 83 | (Letnic and Crowther, 2013) | Index | High | Dingo | Areas with and without control | Rural/Natural |  |
| 91 | (Newsome et al., 2013c) | Proportion sex, Hybridisation | High, Low | Dingo, Feral, Hybrid, Mixed sample | Type of control not reported | Urban, Mine |  |
| 98 | (Brook et al., 2012) | Index, Temporal activity | High | Dingo | Areas with and without control | Rural/Natural |  |
| 103 | (Palmer, 2012) | Diet | High | Dingo | Type of control not reported | Rural/Natural |  |
| 108 | (Kennedy et al., 2012) | Index | High | Dingo | Areas with and without control | Rural/Natural |  |
| 109 | (Allen and Leung, 2012) | Diet | High | Dingo | Areas with and without control | Rural/Natural |  |
| 112 | (Allen, 2012a) | Habitat, Water, Home range, Movement, Temporal activity, Group size, Proportion sex, Proportion age | High, Low | Dingo | Areas without control | Rural/Natural |  |
| 114 | (Radford et al., 2012) | Habitat, Hybridisation | High | Dingo, Feral, Hybrid | Type of control not reported | Environment not reported |  |
| 115 | (Allen, 2012c) | Index, Seasonal activity | High | Unknown wild dog sample | Areas with and without control | Rural/Natural |  |
| 116 | (Wang and Fisher, 2012) | Index, Birth period, Temporal activity, Seasonal activity | High, Low | Unknown wild dog sample | Areas with control | Rural/Natural |  |
| 118 | (Allen, 2012b) | Index, Seasonal activity | High | Unknown wild dog sample | Areas with and without control | Rural/Natural |  |
| 120 | (Moseby et al., 2012) | Litter size, Birth period, Movement | High, Low | Dingo | Areas without control | Animals in captivity |  |
| 123 | (Moseby et al., 2011) | Index, Seasonal activity | High | Dingo | Areas with control | Rural/Natural |  |
| 126 | (Letnic and Dworjanyn, 2011) | Index, Diet | High | Dingo | Areas with and without control | Rural/Natural |  |
| 127 | (Somaweera et al., 2011) | Index, Diet, Temporal activity, Group size | High, Low | Dingo | Areas with and without control | Rural/Natural |  |
| 129 | (Cupples et al., 2011) | Diet | High | Dingo | Areas with and without control | Rural/Natural |  |
| 132 | (Pascoe et al., 2011) | Diet | High | Unknown wild dog sample | Type of control not reported | Rural/Natural |  |
| 133 | (Brawata and Neeman, 2011) | Index, Habitat | High | Dingo | Areas with and without control | Rural/Natural |  |
| 145 | (Wallach et al., 2010) | Index, Habitat | High | Dingo | Areas with and without control | Rural/Natural | (Wallach, 2011) |
| 146 | (Claridge et al., 2010a) | Index, Seasonal activity | High | Unknown wild dog sample | Areas with and without control | Rural/Natural |  |
| 149 | (Letnic and Koch, 2010) | Index | High | Dingo | Type of control not reported | Rural/Natural |  |
| 153 | (Robley et al., 2010) | Habitat, Home range, Movement, Long distance, Temporal activity, Proportion sex, Proportion age, Hybridisation | High, Low | Dingo, Hybrid, Mixed sample | Areas without control | Rural/Natural |  |
| 155 | (Claridge et al., 2010b) | Diet | High | Unknown wild dog sample | Type of control not reported | Rural/Natural |  |
| 157 | (Allen, 2010) | Diet, Birth period, Group size | High, Low | Unknown wild dog sample | Type of control not reported | Rural/Natural |  |
| 160 | (Letnic et al., 2009b) | Index, Diet | High | Dingo | Areas with and without control | Rural/Natural |  |
| 161 | (Claridge et al., 2009) | Home range, Movement, Long distance, Proportion sex, Proportion age, Hybridisation | High, Low | Dingo, Hybrid, Mixed sample | Areas without control | Rural/Natural |  |
| 162 | (Letnic et al., 2009a) | Index | High | Dingo | Areas with and without control | Rural/Natural |  |
| 171 | (Wallach and O'Neill, 2009) | Index | High | Dingo | Areas with and without control | Rural/Natural | (Wallach, 2011) |
| 172 | (Robley et al., 2009) | Home range, Movement, Proportion sex | High, Low | Unknown wild dog sample | Areas without control | Rural/Natural |  |
| 173 | (Wallach et al., 2009a) | Index, Diet | High | Unknown wild dog sample | Areas with and without control | Rural/Natural | (Wallach and O'Neill, 2008)  (Wallach, 2011) |
| 175 | (Wallach et al., 2009b) | Index | High | Dingo | Areas with and without control | Rural/Natural | (Wallach, 2011) |
| 181 | (Pavey et al., 2008) | Index, Diet, Seasonal activity | High | Dingo | Type of control not reported | Rural/Natural |  |
| 184 | (Glen and Dickman, 2008) | Diet | High | Unknown wild dog sample | Areas without control | Rural/Natural | (Australasian Vertebrate Pest Conference, 2005) |
| 187 | (Elledge et al., 2008) | Hybridisation | High | Dingo, Feral, Hybrid | Type of control not reported | Environment not reported |  |
| 200 | (West and Saunders, 2007) | Index | High | Unknown wild dog sample | Entire state | Entire state |  |
| 217 | (Moseby et al., 2006) | Index, Seasonal activity | High | Dingo | Type of control not reported | Rural/Natural |  |
| 219 | (Mitchell and Banks, 2005) | Diet | High | Unknown wild dog sample | Areas with control | Rural/Natural |  |
| 229 | (Koertner and Watson, 2005) | Index | High | Unknown wild dog sample | Areas with control | Rural/Natural |  |
| 231 | (Marks et al., 2004) | Temporal activity, Proportion sex | Low | Mixed sample | Type of control not reported | Rural/Natural |  |
| 245 | (Banks et al., 2003) | Hybridisation | High | Dingo | Areas with control | Rural/Natural |  |
| 256 | (Paltridge, 2002) | Diet | High | Dingo | Type of control not reported | Rural/Natural |  |
| 263 | (Vernes et al., 2001) | Diet | High | Dingo, Hybrid | Type of control not reported | Rural/Natural |  |
| 266 | (Corbett, 2001) | Hybridisation | High | Dingo, Hybrid | Type of control not reported | Environment not reported |  |
| 273 | (Glen and Short, 2000) | Index | High | Dingo | Entire state | Entire state |  |
| 274 | (Vernes, 2000) | Diet | High | Dingo | Type of control not reported | Rural/Natural |  |
| 292 | (Catling et al., 1999) | Index | High | Dingo | Type of control not reported | Rural/Natural |  |
| 296 | (Meek and Triggs, 1998) | Diet | High | Unknown wild dog sample | Type of control not reported | Rural/Natural |  |
| 300 | (Mahon et al., 1998) | Habitat | High | Dingo | Type of control not reported | Rural/Natural |  |
| 306 | (Jones and Johnson, 1997) | Index | Low | Unknown wild dog sample | Type of control not reported | Rural/Natural |  |
| 313 | (Allen et al., 1996) | Index | High | Dingo | Areas with and without control | Rural/Natural |  |
| 314 | (Lunney et al., 1996) | Diet | High | Unknown wild dog sample | Areas with control | Rural/Natural |  |
| 318 | (Southgate et al., 1996) | Diet | Low | Unknown wild dog sample | Type of control not reported | Rural/Natural |  |
| 320 | (Christensen and Burrows, 1994) | Index | High | Dingo | Areas with and without control | Rural/Natural |  |
| 322 | (Corbett, 1995) | Density, Diet | High | Dingo | Type of control not reported | Rural/Natural |  |
| 353 | (Brown and Triggs, 1990) | Diet | High | Unknown wild dog sample | Areas without control | Rural/Natural |  |
| 354 | (Lunney et al., 1990) | Diet | High | Unknown wild dog sample | Type of control not reported | Rural/Natural |  |
| 355 | (Jones, 1990) | Proportion sex, Proportion age | High | Unknown wild dog sample | Type of control not reported | Rural/Natural |  |
| 356 | (Marsack and Campbell, 1990) | Diet, Group size | High | Dingo | Areas without control | Rural/Natural |  |
| 364 | (Corbett, 1989) | Diet | High | Dingo | Type of control not reported | Rural/Natural |  |
| 367 | (Jones and Stevens, 1988) | Litter size, Reproductive age, Birth period, Mating period | High | Unknown wild dog sample | Areas with control | Rural/Natural |  |
| 372 | (Corbett and Newsome, 1987) | Diet | High | Dingo | Type of control not reported | Rural/Natural |  |
| 379 | (Newsome and Corbett, 1985) | Habitat, Hybridisation | High | Dingo, Feral, Hybrid | Type of control not reported | Environment not reported |  |
| 381 | (Harden, 1985) | Home range, Movement, Temporal activity | High | Dingo | Type of control not reported | Rural/Natural |  |
| 382 | (Robertshaw and Harden, 1985b) | Diet | High | Dingo | Type of control not reported | Rural/Natural |  |
| 390 | (Triggs et al., 1984) | Diet | High | Unknown wild dog sample | Type of control not reported | Rural/Natural |  |
| 397 | (Newsome et al., 1983a) | Index, Diet, Group size | High, Low | Unknown wild dog sample | Type of control not reported | Rural/Natural |  |
| 399 | (Newsome et al., 1983b) | Diet | High | Unknown wild dog sample | Areas with control | Rural/Natural |  |
| 400 | (Woodall, 1983) | Index, Mortality | High | Dingo | Entire state | Entire state |  |
| 401 | (Newsome and Corbett, 1982) | Hybridisation | High | Dingo, Feral, Hybrid | Type of control not reported | Rural/Natural |  |
| 405 | (Shepherd, 1981) | Observations, Temporal activity, Group size | High, Low | Dingo | Type of control not reported | Rural/Natural |  |
| 406 | (Catling, 1979) | Mating period | High | Dingo, Hybrid | Not applicable | Animals in captivity |  |
| 407 | (Caughley et al., 1980) | Index | High | Dingo | Type of control not reported | Rural/Natural |  |
| 417 | (Whitehouse, 1977b) | Movement | High | Dingo | Type of control not reported | Rural/Natural |  |
| 422 | (Calaby and Keith, 1974) | Habitat, Group size | Low | Dingo | Areas without control | Rural/Natural |  |
| 441 | (Hodge, 1954) | Mating period, Age independence, Group size, Proportion sex, Proportion age | Low | Dingo | Type of control not reported | Environment not reported |  |
| 458 | (Krefft, 1862) | Litter size | Low | Unknown wild dog sample | Areas with control | Environment not reported |  |
| 504 | (Allen et al., 2015) | Density, Litter size, Mortality, Group size | High | Dingo | Areas with control | Rural/Natural |  |
| 519 | (Read et al., 2015) | Index, Habitat, Temporal activity, Seasonal activity | High | Dingo | Type of control not reported | Rural/Natural |  |
| 612 | (Allen et al., 2012) | Diet | High | Unknown wild dog sample | Areas with control | Rural/Natural, Urban |  |
| 626 | (Glen et al., 2011) | Diet | High | Unknown wild dog sample | Type of control not reported | Rural/Natural |  |
| 632 | (Brook and Kutt, 2011) | Diet | High | Unknown wild dog sample | Type of control not reported | Rural/Natural |  |
| 689 | (Southgate et al., 2007) | Index, Habitat | High | Dingo | Type of control not reported | Rural/Natural |  |
| 696 | (Banks et al., 2006) | Diet, Proportion sex, Proportion age | High, Low | Dingo | Type of control not reported | Rural/Natural |  |
| 729 | (Burrows et al., 2003) | Index, Seasonal activity | High | Dingo | Areas with and without control | Rural/Natural |  |
| 748 | (Edwards et al., 2002b) | Index, Seasonal activity | High | Dingo | Areas with and without control | Rural/Natural | (Edwards et al., 2002c) |
| 754 | (Newsome et al., 2001) | Index, Seasonal activity | High | Dingo | Type of control not reported | Rural/Natural |  |
| 767 | (Eldridge et al., 2000) | Index | High | Dingo | Areas with control | Rural/Natural |  |
| 791 | (Fleming, 1996c) | Density, Index | High | Unknown wild dog sample | Areas with and without control | Rural/Natural |  |
| 793 | (Catling and Burt, 1995b) | Index | High | Unknown wild dog sample | Areas with control | Rural/Natural |  |
| 798 | (Lundiejenkins et al., 1993) | Index, Habitat, Diet, Seasonal activity | High | Dingo | Areas with control | Rural/Natural |  |
| 805 | (Catling et al., 1992) | Litter size, Litter sex, Reproductive age, Birth period, Mating period | High | Dingo, Hybrid | Type of control not reported | Rural/Natural, Animals in captivity |  |
| 806 | (Thomson, 1992a) | Proportion sex, Proportion age | High | Dingo | Areas with and without control | Rural/Natural |  |
| 807 | (Thomson, 1992b) | Index, Birth period, Mating period, Contact, Movement, Age independence, Temporal activity, Seasonal activity | High | Dingo | Areas with and without control | Rural/Natural |  |
| 809 | (Thomson, 1992c) | Habitat, Contact, Home range, Dispersal, Movement, Seasonal activity, Group size, Proportion sex, Proportion age | High | Dingo | Areas without control | Rural/Natural |  |
| 810 | (Thomson et al., 1992a) | Density, Habitat, Birth rate, Litter size, Reproductive age, Mortality, Contact, Home range, Dispersal, Movement, Seasonal activity, Group size, Proportion sex, Proportion age | High, Low | Dingo | Areas with control | Rural/Natural |  |
| 811 | (Thomson et al., 1992b) | Mortality, Dispersal, Long distance, Seasonal activity, Group size, Proportion sex, Proportion age | High, Low | Dingo | Areas without control | Rural/Natural |  |
| 830 | (Hudson et al., 2016) | Litter size, Litter sex, Birth period | Low | Dingo | Not applicable | Animals in captivity |  |
| 837 | (Behrendorff et al., 2016) | Diet | High | Dingo | Areas with control | Rural/Natural |  |
| 839 | (Allen et al., 2016a) | Diet | High | Unknown wild dog sample | Type of control not reported | Rural/Natural, Urban |  |
| 921 | (Allen et al., 2014) | Home range, Movement, Temporal activity, Seasonal activity, Proportion sex | High, Low | Unknown wild dog sample | Areas with and without control | Rural/Natural |  |
| 925 | (Ballard et al., 2014) | Index | High | Unknown wild dog sample | Type of control not reported | Rural/Natural |  |
| 961 | (Arthur et al., 2012) | Index, Habitat, Seasonal activity | High | Unknown wild dog sample | Type of control not reported | Rural/Natural |  |
| 1104 | (Glen et al., 2006) | Diet | High | Unknown wild dog sample | Areas with control | Rural/Natural |  |
| 1153 | (Edwards et al., 2002a) | Index, Habitat | High | Dingo | Type of control not reported | Rural/Natural |  |
| 1178 | (Pople et al., 2000) | Density | High | Dingo | Areas with and without control | Rural/Natural |  |
| 1179 | (Twigg et al., 2000) | Index | High | Dingo | Areas with and without control | Rural/Natural |  |
| 1216 | (Fleming et al., 1996) | Index | High | Unknown wild dog sample | Areas with and without control | Rural/Natural |  |
| 1222 | (Catling and Burt, 1995a) | Habitat | High | Unknown wild dog sample | Type of control not reported | Rural/Natural |  |
| 1234 | (Jarman and Wright, 1993) | Observations, Habitat, Temporal activity, Seasonal activity, Group size | High, Low | Dingo | Areas without control | Rural/Natural |  |
| 1241 | (Thomson, 1992d) | Diet, Group size | High | Dingo | Areas with and without control | Rural/Natural |  |
| 1273 | (Thomson, 1986) | Observations, Mortality, Group size | High, Low | Dingo | Areas with and without control | Rural/Natural |  |
| 1275 | (McLlroy et al., 1986) | Density, Mortality, Home range, Movement, Group size, Proportion sex, Proportion age | High, Low | Unknown wild dog sample | Areas with control | Rural/Natural |  |
| 1283 | (Robertshaw and Harden, 1985a) | Diet | High | Dingo | Areas with and without control | Rural/Natural |  |
| 1306 | (Dwyer et al., 1979) | Observations | High | Unknown wild dog sample | Type of control not reported | Rural/Natural |  |
| 1310 | (Whitehouse, 1977a) | Diet | High | Dingo | Entire state | Entire state |  |
| 1318 | (Corbett and Newsome, 1975) | Water, Litter size, Contact, Age independence, Group size, Proportion sex, Proportion age | High, Low | Dingo | Type of control not reported | Rural/Natural |  |
| 1320 | (Best et al., 1974) | Index | High | Dingo | Areas with and without control | Rural/Natural |  |
| 1325 | (Newsome et al., 1972) | Density | High | Dingo | Areas with and without control | Rural/Natural |  |
| 1329 | (Calaby, 1966) | Habitat | Low | Dingo | Areas with control | Rural/Natural |  |
| 1348 | (Tomlinson and Blair, 1952) | Dispersal | Low | Unknown wild dog sample | Areas with control | Rural/Natural |  |
| 1358 | (Sparkes et al., 2016) | Index, Observations, Contact, Temporal activity, Seasonal activity, Group size | High | Mixed sample | Type of control not reported | Rural/Natural |  |
| 1373 | (Schroeder et al., 2015) | Habitat | High | Dingo | Areas without control | Animals in captivity |  |
| 1374 | (Colman et al., 2015) | Index, Habitat | High | Dingo | Areas with and without control | Rural/Natural |  |
| 1522 | (Jenkins et al., 2000) | Movement | High | Unknown wild dog sample | Type of control not reported | Rural/Natural |  |
| 1526 | (Balogh, 2000) | Diet, Movement, Proportion sex, Proportion age, Hybridisation | High, Low | Dingo, Hybrid, Mixed sample | Type of control not reported | Rural/Natural |  |
| 1574 | (Fleming and Korn, 1989) | Observations, Seasonal activity, Group size | High, Low | Unknown wild dog sample | Areas with control | Rural/Natural |  |
| 1603 | (Whitehouse, 1978) | Movement, Proportion sex, Habitat, Home range, Temporal activity, Seasonal activity, Group size, Proportion age | High, Low | Dingo | Type of control not reported | Rural/Natural |  |
| 1616 | (Coman, 1972) | Diet | High | Mixed sample | Areas with control | Rural/Natural |  |
| 1642 | (Tomlinson, 1954) | Observations, Habitat, Group size | High, Low | Unknown wild dog sample | Areas with control | Rural/Natural |  |
| 1650 | (Durie and Riek, 1952) | Density | High | Dingo | Entire state | Entire state |  |
| 1682 | (Stephens, 2011b) | Proportion sex, Hybridisation | High, Low | Dingo, Hybrid, Mixed sample | Type of control not reported | Rural/Natural, Mine |  |
| 1682 | (Stephens, 2011a) | Index | High | Unknown wild dog sample | Areas with and without control | Rural/Natural |  |
| 1683 | (Brook, 2013d) | Contact | High | Dingo | Type of control not reported | Rural/Natural |  |
| 1683 | (Brook, 2013a) | Diet | High | Dingo | Areas with and without control | Rural/Natural |  |
| 1683 | (Brook, 2013b) | Index, Habitat, Seasonal activity | High | Dingo | Areas with and without control | Rural/Natural |  |
| 1683 | (Brook, 2013c) | Home range, Temporal activity, Proportion sex | High, Low | Dingo | Type of control not reported | Rural/Natural |  |
| 1684 | (Wysong, 2016a) | Index, Habitat | High | Dingo | Areas without control | Rural/Natural |  |
| 1684 | (Wysong, 2016b) | Diet | High | Dingo | Areas without control | Rural/Natural |  |
| 1684 | (Wysong, 2016c) | Habitat, Home range, Proportion sex | High, Low | Dingo | Areas without control | Rural/Natural |  |
| 1686 | (Leo, 2016a) | Index | High | Dingo | Areas with and without control | Rural/Natural |  |
| 1686 | (Leo, 2016b) | Index | High | Dingo | Areas with and without control | Rural/Natural |  |
| 1686 | (Leo, 2016c) | Index, Temporal activity, Seasonal activity | High | Dingo | Areas with and without control | Rural/Natural |  |
| 1688 | (Morrant, 2015b) | Diet | High | Unknown wild dog sample | Areas with control | Rural/Natural, Urban |  |
| 1688 | (Morrant, 2015a) | Habitat, Home range, Movement, Temporal activity, Proportion sex, Proportion age | High, Low | Unknown wild dog sample | Type of control not reported | Rural/Natural, Urban | (Morrant et al., 2017) |
| 1690 | (Gordon, 2015c) | Index, Diet, Seasonal activity | High, Low | Dingo | Areas with and without control | Rural/Natural |  |
| 1690 | (Gordon, 2015a) | Index | High | Dingo | Areas with and without control | Rural/Natural |  |
| 1690 | (Gordon, 2015b) | Index, Diet | High, Low | Dingo | Areas with and without control | Rural/Natural |  |
| 1691 | (Purcell, 2008b) | Index, Diet, Seasonal activity | High | Unknown wild dog sample | Areas with control | Rural/Natural |  |
| 1691 | (Purcell, 2008c) | Proportion sex, Hybridisation | High, Low | Dingo, Hybrid | Areas with control | Rural/Natural |  |
| 1691 | (Purcell, 2008a) | Habitat, Birth period, Contact, Home range, Dispersal, Movement, Long distance, Temporal activity, Seasonal activity, Proportion age | High, Low | Dingo, Hybrid | Areas with control | Rural/Natural | (Purcell BV et al., 2006) |
| 1693 | (Brawata, 2012a) | Index | High | Dingo | Areas with and without control | Rural/Natural |  |
| 1693 | (Brawata, 2012b) | Habitat | High | Dingo | Areas with and without control | Rural/Natural |  |
| 1693 | (Brawata, 2012c) | Temporal activity | High | Dingo | Areas with and without control | Rural/Natural |  |
| 1694 | (Newsome and Science, 2011b) | Habitat | High | Dingo | Type of control not reported | Rural/Natural | (Newsome et al., 2013a) |
| 1694 | (Newsome and Science, 2011a) | Diet | High | Dingo | Type of control not reported | Rural/Natural | (Newsome et al., 2014b) |
| 1698 | (Allen, 2005a) | Index, Seasonal activity, Proportion age, Diet | High | Unknown wild dog sample | Areas with and without control, Areas with control | Rural/Natural | (Allen, 2015)  (Allen, 2014)  (Allen LR and Gonzalez A, 1998) |
| 1698 | (Allen, 2005b) | Index, Diet, Seasonal activity | High | Unknown wild dog sample | Areas with control | Rural/Natural |  |
| 1699 | (McBride, 2007) | Habitat, Home range, Proportion sex, Proportion age | High, Low | Unknown wild dog sample | Areas with and without control | Rural/Natural |  |
| 1703 | (Corbett, 1974b) | Hybridisation | High | Dingo, Feral, Hybrid | Areas with and without control | Rural/Natural | (Corbett, 1974a) |
| 1703 | (Corbett, 1974c) | Index, Diet, Birth rate, Litter size, Litter sex, Reproductive age, Birth period, Mating period, Proportion sex, Proportion age | High, Low | Mixed sample | Areas with and without control | Rural/Natural | (Corbett, 1974a) |
| 1709 | (Paltridge, 2005) | Index, Habitat, Seasonal activity | High, Low | Dingo | Type of control not reported | Rural/Natural |  |
| 1713 | (May, 2001b) | Index, Habitat | High | Unknown wild dog sample | Areas with and without control | Rural/Natural |  |
| 1713 | (May, 2001a) | Index, Habitat, Seasonal activity | High | Unknown wild dog sample | Areas with and without control | Rural/Natural |  |
| 1713 | (May, 2001c) | Diet | High | Unknown wild dog sample | Areas with and without control | Rural/Natural |  |
| 1713 | (May, 2001d) | Habitat | High | Unknown wild dog sample | Areas with and without control | Rural/Natural |  |
| 1717 | (Fleming, 1996a) | Index, Density | High | Unknown wild dog sample | Areas with and without control | Rural/Natural |  |
| 1717 | (Fleming, 1996b) | Index, Seasonal activity | High | Unknown wild dog sample | Areas with and without control | Rural/Natural |  |
| 1722 | (Doherty, 2015b) | Index, Habitat | High | Dingo | Type of control not reported | Rural/Natural |  |
| 1722 | (Doherty, 2015a) | Index, Habitat, Diet, Temporal activity | High | Dingo | Areas with control | Rural/Natural | (Doherty, 2015c) |
| 1731 | (Foulkes, 2001) | Index, Habitat, Diet | High | Dingo | Type of control not reported | Rural/Natural |  |
| 1749 | (Pascoe and Sciences, 2011b) | Index, Temporal activity, Seasonal activity | High | Unknown wild dog sample | Areas with and without control | Rural/Natural |  |
| 1749 | (Pascoe and Sciences, 2011a) | Index, Habitat | High | Unknown wild dog sample | Areas with and without control | Rural/Natural |  |
| 1766 | (Andrew, 2005) | Diet | High | Dingo | Type of control not reported | Rural/Natural |  |
| 2618 | (Department of Natural Resources, 2014) | Proportion sex, Proportion age, Hybridisation | High, Low | Dingo, Hybrid | Entire state | Entire state |  |
| 2619 | (Stephen R. Eldridge et al., 2002) | Home range, Movement, Index, Observations, Diet, Hybridisation | High | Dingo, Hybrid, Mixed sample | Areas with and without control, Areas without control | Rural/Natural |  |
| 2623 | (Bird, 1994) | Index, Observations, Water, Mortality, Contact, Long distance | High, Low | Dingo | Areas with and without control, Areas with control | Rural/Natural |  |
| 2624 | (Meek and Brown, 2016) | Contact, Temporal activity, Group size | Low | Dingo | Type of control not reported | Mine |  |
| 2664 | (Robertshaw and Harden, 1986) | Index, Observations, Diet, Seasonal activity | High, Low | Dingo | Areas with and without control | Rural/Natural |  |
| 2683 | (Read and Eldridge, 2010) | Index | High | Dingo | Type of control not reported | Rural/Natural |  |
| 2685 | (Edwards et al., 2000) | Index | High | Dingo | Type of control not reported | Rural/Natural |  |
| 2708 | (Hunt et al., 2007) | Index | High | Unknown wild dog sample | Areas without control | Rural/Natural |  |
| 2822 | (Eldridge S.R. et al., 2016) | Index, Diet, Seasonal activity, Water, Proportion sex | High, Low | Unknown wild dog sample | Areas with and without control | Rural/Natural |  |
| 2853 | (Angel, 2006a) | Index, Seasonal activity | High | Dingo | Areas without control | Rural/Natural |  |
| 2853 | (Angel, 2006c) | Habitat | High | Dingo | Areas without control | Rural/Natural |  |
| 2853 | (Angel, 2006b) | Diet | High | Dingo | Areas without control | Rural/Natural |  |
| 2856 |  | Mortality | High | Dingo | Areas with control | Rural/Natural |  |
| 2856 | (Corbett, 2009) | Observations, Proportion sex, Proportion age | High | Dingo | Areas without control | Rural/Natural |  |
| 2857 | (Queensland and Wildlife, 2010) | Density, Long distance, Temporal activity, Proportion sex, Proportion age | High, Low | Dingo | Areas without control | Rural/Natural |  |
| 2868 | (Queensland. Environmental Protection, 2001) | Observations | Low | Dingo | Areas with and without control | Rural/Natural |  |
| 2875 | (Appleby and Jones) | Density, Proportion sex, Proportion age | High | Dingo | Type of control not reported | Rural/Natural |  |
| 2879 | (Baxter and Davies, 2013) | Habitat, Mortality, Home range, Movement, Temporal activity, Seasonal activity, Group size, Proportion sex, Proportion age | High, Low | Dingo | Areas without control | Rural/Natural |  |
| 2883 | (Corbett, 1998) | Density, Birth rate, Litter size, Mating period, Group size, Proportion sex, Proportion age | High, Low | Unknown wild dog sample | Areas without control | Rural/Natural |  |
| 2887 | (Gonzalez et al., 2000) | Index, Diet, Age independence, Seasonal activity, Proportion sex, Proportion age, Hybridisation | High, Low | Dingo, Hybrid, Unknown wild dog sample | Areas with and without control | Rural/Natural |  |
| 2890 | (Lawrance and Higginbottom, 2002) | Observations, Birth period, Contact, Movement, Long distance, Age independence, Group size, Proportion sex, Proportion age | High, Low | Dingo | Areas without control | Rural/Natural |  |
| 2902 | (Australian Vertebrate Pest Control and Queensland. Rural Lands Protection, 1987) | Mortality | High | Dingo | Areas with control | Rural/Natural |  |
| 2903 | (Mitchell et al., 1982) | Density, Habitat, Group size, Hybridisation | High, Low | Mixed sample | Entire state | Entire state |  |
| 2909 | (Tierney and Strong, 1987) | Index | High | Dingo | Areas with and without control | Rural/Natural |  |
| 2924 | (Augusteyn et al., 2010) | Diet, Index, Seasonal activity | High | Unknown wild dog sample | Areas with control | Rural/Natural |  |
| 2927 | (McNeill et al., 2016) | Habitat, Home range, Movement, Temporal activity, Proportion sex, Proportion age | High, Low | Unknown wild dog sample | Type of control not reported | Urban |  |
| 2930 | (Allen et al., 2016b) | Home range, Movement, Temporal activity, Proportion sex, Proportion age | High, Low | Unknown wild dog sample | Areas without control | Rural/Natural |  |
| 2932 | (Allen and Byrne, 2008) | Habitat, Birth period, Home range, Dispersal, Long distance, Age independence, Seasonal activity | High, Low | Unknown wild dog sample | Areas with and without control | Environment not reported |  |
| 3166 | (Reynolds et al., 2016) | Diet | Low | Unknown wild dog sample | Type of control not reported | Environment not reported |  |
| 3431 | (Hernandez-Santin et al., 2016) | Habitat, Temporal activity | High | Dingo | Areas with and without control | Rural/Natural |  |
| 3570 | (Goldingay and Whelan, 1997) | Index, Habitat | Low | Unknown wild dog sample | Type of control not reported | Rural/Natural |  |
| 3679 | (Corbett, 1988) | Birth rate, Litter size, Litter sex, Reproductive age, Birth period, Mating period, Mortality, Contact, Group size, Proportion sex, Proportion age | High, Low | Dingo | Not applicable | Animals in captivity |  |
| 3683 | (Coman and Robinson, 1989) | Habitat, Litter size, Movement | High, Low | Feral | Type of control not reported | Urban |  |
| 5433 | (Catling et al., 2000) | Habitat, Seasonal activity | High | Unknown wild dog sample | Type of control not reported | Rural/Natural |  |
| 5437 | (Wilton, 2001) | Hybridisation | Low | Unknown wild dog sample | Type of control not reported | Rural/Natural |  |
| 5448 | (Best, 1978) | Home range, Long distance, Group size | High | Dingo | Type of control not reported | Rural/Natural |  |
| 5448 | (Catling, 1978) | Density, Habitat, Home range, Long distance, Temporal activity | High, Low | Dingo | Type of control not reported | Rural/Natural |  |
| 5464 | (Newsome and Catling, 1979) | Index, Habitat | High | Dingo | Type of control not reported | Rural/Natural |  |
| 5483 | (Corbett L, 1983) | Diet | High | Dingo | Type of control not reported | Rural/Natural |  |
| 5485 | (Eldridge DT and Bryan R, 1995) | Index, Habitat | Low | Dingo | Entire state | Entire state |  |

**List of references for all included studies in the scoping review**

Allen, B. (2006a). "Urban dingoes (*Canis lupus dingo* and hybrids) and human hydatid disease (*Echinococcus granulosus*) in Queensland, Australia", in: *Proceedings of the 22nd Vertebrate Pest Conference.*).

Allen, B. (2007). "The spatial ecology and zoonoses of urban dingoes, and the use of Traversed Area Polygons (TAPs) to calculate home range sizes", in: *Proceedings of a workshop on remote monitoring of wild canids and felids.* (eds.) P. Fleming & D. Jenkins. (Australian National University, Canberra).

Allen, B.L. (2006b). *The spatial ecology and zoonoses of urban dingoes – a preliminary investigation.* Bachelor of Applied Science (Animal Studies) (Honours), University of Queensland.

Allen, B.L. (2010). Skin and bone: observations of dingo scavenging during a chronic food shortage. *Australian Mammalogy* 32(2)**,** 207-208.

Allen, B.L. (2012a). Do desert dingoes drink daily? Visitation rates at remote waterpoints in the Strzelecki Desert. *Australian Mammalogy* 34(2)**,** 251-256.

Allen, B.L. (2012b). "The effects of lethal control on the conservation values of Canis lupus dingo," in *Wolves: biology, behavior and conservation. [Animal Science, Issues and Professions.],* eds. A.P. Maia & H.F. Crussi. Nova Science Publishers, Inc.), 79-108.

Allen, B.L. (2012c). Scat happens: spatiotemporal fluctuation in dingo scat collection rates. *Australian Journal of Zoology* 60(2)**,** 137-140.

Allen, B.L., Allen, L.R., Engeman, R.M., and Leung, L.K.P. (2013a). Intraguild relationships between sympatric predators exposed to lethal control: predator manipulation experiments. *Frontiers in Zoology* 10**,** 1-18.

Allen, B.L., Carmelito, E., Amos, M., Goullet, M.S., Allen, L.R., Speed, J., et al. (2016a). Diet of dingoes and other wild dogs in peri-urban areas of north-eastern Australia. *Scientific Reports* 6**,** 23028. doi: 10.1038/srep23028.

Allen, B.L., Engeman, R.M., and Leung, L.K.P. (2014). The short-term effects of a routine poisoning campaign on the movements and detectability of a social top-predator. *Environmental Science and Pollution Research International* 21(3)**,** 2178-2190. doi: 10.1007/s11356-013-2118-7.

Allen, B.L., Goullet, M., Allen, L.R., Lisle, A., and Leung, L.K.P. (2013b). Dingoes at the doorstep: Preliminary data on the ecology of dingoes in urban areas. *Landscape and Urban Planning* 119**,** 131-135.

Allen, B.L., Higginbottom, K., Bracks, J.H., Davies, N., and Baxter, G.S. (2015). Balancing dingo conservation with human safety on Fraser Island: the numerical and demographic effects of humane destruction of dingoes. *Australasian Journal of Environmental Management* 22(2)**,** 197-215. doi: 10.1080/14486563.2014.999134.

Allen, B.L., and Leung, L.K.P. (2012). Assessing predation risk to threatened fauna from their prevalence in predator scats: dingoes and rodents in arid Australia. *PLoS ONE* 7(5)**,** e36426, 36421-36429.

Allen, B.L., and Leung, L.K.P. (2014). The (non)effects of lethal population control on the diet of Australian dingoes. *PLoS ONE* 9(9)**,** e108251.

Allen, L., and Byrne, D. (2008). "Are we focussing wild dog control the wrong time of the year and going about it the wrong way? ", in: *Proceedings of the 14th Australasian Vertebrate Pest Conference.* (Darwin).

Allen, L., Engeman, R., and Krupa, H. (1996). Evaluation of three relative abundance indices for assessing dingo populations. *Wildlife Research* 23(2)**,** 197-206. doi: 10.1071/wr9960197.

Allen, L., Goullet, M., and Palmer, R. (2012). The diet of the dingo (*Canis lupus dingo* and hybrids) in north-eastern Australia: a supplement to the paper of Brook and Kutt (2011). *Rangeland Journal* 34(2)**,** 211-217. doi: 10.1071/rj11092.

Allen, L.R. (2005a). "Chapter 3. Results of wild dog monitoring studies," in *The impact of wild dog predation and wild dog control on beef cattle production*. (University of Queensland), 137-164.

Allen, L.R. (2005b). "Chapter 5. Calf predation assessment," in *The impact of wild dog predation and wild dog control on beef cattle production*. (University of Queensland), 213-234.

Allen, L.R. (2014). Wild dog control impacts on calf wastage in extensive beef cattle enterprises. *Animal Production Science* 54(2)**,** 214-220. doi: 10.1071/an12356.

Allen, L.R. (2015). Demographic and functional responses of wild dogs to poison baiting. *Ecological Management & Restoration* 16(1)**,** 58-66.

Allen LR, and Gonzalez A (1998). "Bating reduces dingo numbers, changes age structures yet often increases calf losses", in: *11th Australian Vertebrate Pest Conference.* (Bunbury, Western Australia).

Allen, L.R., Stewart-Moore, N., Byrne, D., and Allen, B.L. (2016b). Guardian dogs protect sheep by guarding sheep, not by establishing territories and excluding predators. *Animal Production Science***,** -. doi: http://dx.doi.org/10.1071/AN16030.

Andrew, D.L. (2005). *Ecology of the tiger quoll dasyurus maculatus maculatus in coastal New South Wales.* thesis School of Biological Sciences - Faculty of Science.

Angel, D.C. (2006a). "Chapter 3. Prey communities," in *Dingo diet and prey availability on Fraser Island*. University of the Sunshine Coast), 32-82.

Angel, D.C. (2006b). "Chapter 4. Dingo diet," in *Dingo diet and prey availability on Fraser Island*. University of the Sunshine Coast), 83-114.

Angel, D.C. (2006c). "Chapter 5. Predator-prey relationships," in *Dingo diet and prey availability on Fraser Island*. University of the Sunshine Coast), 115-136.

Appleby, R., and Jones, D. (2011). "Analysis of a preliminary dingo capture-mark-recapture experiment on Fraser Island". (Brisbane: Queensland Parks and Wildlife Service).

Appleby, R., Smith, B., and Jones, D. (2013). Observations of a free-ranging adult female dingo (*Canis dingo*) and littermates' responses to the death of a pup. *Behavioural Processes* 96**,** 42-46.

Arthur, A.D., Catling, P.C., and Reid, A. (2012). Relative influence of habitat structure, species interactions and rainfall on the post-fire population dynamics of ground-dwelling vertebrates. *Austral Ecology* 37(8)**,** 958-970. doi: 10.1111/j.1442-9993.2011.02355.x.

Augusteyn, J., Hemson, G., Bennison, K.A., Allen, L.R., Wyland, J., Nolan, B.J., et al. (2010). "Determining the effectiveness of wild dog control at Taunton National Park (Scientific) and its impact on the population of bridled nailtail wallabies", in: *Proceedings of the Queensland Pest Animal Symposium, Gladstone, Queensland.* (Rockhampton, Qld.: Department of Environment and Resource Management).

Australasian Vertebrate Pest Conference (2005). "13th Australasian Vertebrate Pest Conference, Wellington, New Zealand, 2-6 May 2005".).

Australian Vertebrate Pest Control, C., and Queensland. Rural Lands Protection, B. (Year). "Handbook of working papers : 8th Australian Vertebrate Pest Control Conference, Coolangatta, Queensland, May 1987", in: *Australian Vertebrate Pest Control Conference*, (Websites QL Liberty: The Conference Committee).

Ballard, G., Meek, P.D., Doak, S., Fleming, P.J.S., and Sparkes, J. (2014). *Camera traps, sand plots and known events: what do camera traps miss?* : Csiro Publishing, Unipark, Bldg 1, Level 1, 195 Wellington Rd, Locked Bag 10, Clayton, Vic 3168, Australia.

Balogh, S. (Year). "Practical solutions to pest management problems. Proceedings of the NSW Pest Animal Control Conference, Orange, New South Wales, Australia, 25-27 October 2000", in: *Practical solutions to pest management problems. Proceedings of the NSW Pest Animal Control Conference, Orange, New South Wales, Australia, 25-27 October 2000.*, ed. S. Balogh (CAB Abstract: NSW Agriculture), 111.

Banks, D.J.D., Copeman, D.B., and Skerratt, L.F. (2006). Echinococcus granulosus in northern Queensland. 2. Ecological determinants of infection in beef cattle. *Australian Veterinary Journal* 84(9)**,** 308-311. doi: 10.1111/j.1751-0813.2006.00021.x.

Banks, S.C., Horsup, A., Wilton, A.N., and Taylor, A.C. (2003). Genetic marker investigation of the source and impact of predation on a highly endangered species. *Molecular Ecology* 12(6)**,** 1663-1667. doi: 10.1046/j.1365-294X.2003.01823.x.

Bannister, H.L., Lynch, C.E., and Moseby, K.E. (2016). Predator swamping and supplementary feeding do not improve reintroduction success for a threatened Australian mammal, Bettongia lesueur. *Australian Mammalogy* 38(2)**,** 177-187.

Baxter, G., and Davies, N. (2013). "Tracking dingoes on Fraser Island : final report". (Brisbane, Qld.: University of Queensland).

Behrendorff, L., and Allen, B.L. (2016). From den to dust: longevity of three dingoes (*Canis lupus dingo*) on Fraser Island (K'gari). *Australian Mammalogy* 38(2)**,** 256-260.

Behrendorff, L., Leung, L.K.P., McKinnon, A., Hanger, J., Belonje, G., Tapply, J., et al. (2016). Insects for breakfast and whales for dinner: the diet and body condition of dingoes on Fraser Island (K'gari). *Scientific Reports* 6**,** 23469. doi: 10.1038/srep23469.

Best, L.W. (1978). "Dingo movements in central Australia", in: *Australian vertebrate pest control conference 1978: working papers.* (ed.) Anon. (Canberra).

Best, L.W., Corbett, L.K., Stephens, D.R., and Newsome, A.E. (1974). "Baiting trials for dingoes in central Australia with poison 1080 encapsulated strychnine and strychnine suspended in methyl cellulose", in: *Division of Wildlife Research technical paper ; no. 30.*).

Bird, P. (1994). "Improved electric fences and baiting techniques - A behavioural approach to integrated dingo control", (ed.) P.i.S.A. Animal and plant control commission.).

Brawata, R.L. (2012a). "Chapter 4: The influence of management of the dingo on the response of mesopredators and prey to rainfall in arid ecosystems," in *Is the dingo top dog? : the influence of dingo management on the behaviour of introduced carnivores in arid Australia, with implications for native fauna conservation*.), 97-118.

Brawata, R.L. (2012b). "Chapter 5: Habitat use by dingoes, foxes and feral cats and select prey under different dingo management strategies," in *Is the dingo top dog? : the influence of dingo management on the behaviour of introduced carnivores in arid Australia, with implications for native fauna conservation*.), 119-140.

Brawata, R.L. (2012c). "Chapter 7: Temporal visitations and behaviour of dingoes, foxes and feral cats under different dingo management strategies," in *Is the dingo top dog? : the influence of dingo management on the behaviour of introduced carnivores in arid Australia, with implications for native fauna conservation*.), 153-175.

Brawata, R.L., and Neeman, T. (2011). Is water the key? Dingo management, intraguild interactions and predator distribution around water points in arid Australia. *Wildlife Research* 38(5)**,** 426-436.

Brook, L.A. (2013a). "Appendix A Notes on prey consumed by dingoes and feral cats in northern Australia," in *Predator guild interactions in northern Australia: behaviour and ecology of an apex predator, the dingo Canis lupus dingo, and an introduced mesopredator, the feral cat Felis catus*. James Cook University), 179-192.

Brook, L.A. (2013b). "Chapter 3 Hiding in plain sight: Do feral cats use complex habitats in north Queensland to avoid encounters with dingoes?," in *Predator guild interactions in northern Australia: behaviour and ecology of an apex predator, the dingo Canis lupus dingo, and an introduced mesopredator, the feral cat Felis catus*. James Cook University), 43-68.

Brook, L.A. (2013c). "Chapter 4 Seasonal space use of dingoes and feral cats: differences in patterns of intensity in northern Australian savanna," in *Predator guild interactions in northern Australia: behaviour and ecology of an apex predator, the dingo Canis lupus dingo, and an introduced mesopredator, the feral cat Felis catus*. James Cook University), 69-100.

Brook, L.A. (2013d). "Chapter 5 Spatial interactions between sympatric dingoes and feral cats in the Kimberley: patterns of intraguild avoidance in a landscape of fear," in *Predator guild interactions in northern Australia: behaviour and ecology of an apex predator, the dingo Canis lupus dingo, and an introduced mesopredator, the feral cat Felis catus*. James Cook University), 101-130.

Brook, L.A., Johnson, C.N., and Ritchie, E.G. (2012). Effects of predator control on behaviour of an apex predator and indirect consequences for mesopredator suppression. *Journal of Applied Ecology* 49(6)**,** 1278-1286.

Brook, L.A., and Kutt, A.S. (2011). The diet of the dingo (*Canis lupus dingo*) in north-eastern Australia with comments on its conservation implications. *Rangeland Journal* 33(1)**,** 79-85. doi: 10.1071/rj10052.

Brown, G.W., and Triggs, B.E. (1990). Diets of wild canids and foxes in east Gippsland 1983-1987, using predator scat analysis. *Australian Mammalogy* 13(1-2)**,** 209-213.

Burrows, N.D., Algar, D., Robinson, A.D., Sinagra, J., Ward, B., and Liddelow, G. (2003). Controlling introduced predators in the Gibson Desert of Western Australia. *Journal of Arid Environments* 55(4)**,** 691-713. doi: 10.1016/s0140-1963(02)00317-8.

Calaby, J.H. (1966). *Mammals of the Upper Richmond and Clarence Rivers, New South Wales.*

Calaby, J.H., and Keith, K. (1974). "Mammals," in *Fauna survey of the Port Essington District, Cobourg Peninsula, Northern Territory of Australia* ), 179-208.

Catling, P.C. (1978). "Dingo movements in south-eastern New South Wales", in: *Australian vertebrate pest control conference 1978: working papers.* (ed.) Anon. (Canberra).

Catling, P.C. (1979). Seasonal variation in plasma testosterone and the testis in captive male dingoes, *Canis familiaris dingo*. *Australian Journal of Zoology* 27(6)**,** 939-944. doi: 10.1071/zo9790939.

Catling, P.C., and Burt, R.J. (1995a). Studies of the ground-dwelling mammals of eucalypt forests in South-eastern New South Wales: The effect of habitat variables on distribution and abundance. *Wildlife Research* 22(3)**,** 271-288. doi: 10.1071/wr9950271.

Catling, P.C., and Burt, R.J. (1995b). Why are red foxes absent from some eucalypt forests in eastern New South Wales. *Wildlife Research* 22(5)**,** 535-546. doi: 10.1071/wr9950535.

Catling, P.C., Burt, R.J., and Forrester, R.I. (2000). Models of the distribution and abundance of ground-dwelling mammals in the eucalypt forests of north-eastern New South Wales in relation to habitat variables. *Wildlife Research* 27**,** 639–654.

Catling, P.C., Corbett, L.K., and Newsome, A.E. (1992). Reproduction in captive and wild dingoes (*Canis familiaris dingo*) in temperate and arid environments of Australia. *Wildlife Research* 19(2)**,** 195-209. doi: 10.1071/wr9920195.

Catling, P.C., Hertog, A., Burt, R.J., Wombey, J.C., and Forrester, R.I. (1999). The short-term effect of cane toads (*Bufo marinus*) on native fauna in the Gulf County of the Northern Territory. *Wildlife Research* 26(2)**,** 161-185. doi: 10.1071/wr98025.

Caughley, G., Grigg, G.C., Caughley, J., and Hill, G.J.E. (1980). Does dingo predation control the densities of kangaroos and emus? *Australian Wildlife Research* 7(1)**,** 1-12.

Christensen, P., and Burrows, N. (1994). "Chapter 32. Project desert dreaming: experimental reintroduction of mammals to the Gibson Desert, Western Australia," in *Reintroduction biology of Australian and New Zealand fauna.,* ed. M. Serena. Surrey Beatty & Sons), 199-207.

Claridge, A.W., Cunningham, R.B., Catling, P.C., and Reid, A.M. (2010a). Trends in the activity levels of forest-dwelling vertebrate fauna against a background of intensive baiting for foxes. *Forest Ecology and Management* 260(5)**,** 822-832.

Claridge, A.W., Mills, D.J., and Barry, S.C. (2010b). Prevalence of threatened native species in canid scats from coastal and near-coastal landscapes in south-eastern Australia. *Australian Mammalogy* 32(2)**,** 117-126.

Claridge, A.W., Mills, D.J., Hunt, R., Jenkins, D.J., and Bean, J. (2009). Satellite tracking of wild dogs in south-eastern mainland Australian forests: implications for management of a problematic top-order carnivore. *Forest Ecology and Management* 258(5)**,** 814-822.

Colman, N.J., Crowther, M.S., and Letnic, M. (2015). Macroecological patterns in mammal abundances provide evidence that an apex predator shapes forest ecosystems by suppressing herbivore and mesopredator abundance. *Journal of Biogeography* 42(10)**,** 1975-1985. doi: 10.1111/jbi.12563.

Coman, B.J. (1972). Helminth parasites of the dingo and feral dog in Victoria with some notes on the diet of the host. *Australian Veterinary Journal* 48(8)**,** 456-461.

Coman, B.J., and Robinson, J.L. (1989). Some aspects of stray dog behaviour in an urban fringe area. *Australian veterinary journal* 66(1)**,** 30-32.

Corbett, L. (1995). Does dingo predation or buffalo competition regulate feral pig populations in the Australian wet-dry tropics? An experimental study. *Wildlife Research* 22(1)**,** 65-74. doi: 10.1071/wr9950065.

Corbett, L. (2001). "The conservation status of the dingo *Canis lupus dingo* in Australia, with particular reference to New South Wales: threats to pure dingoes and potential solutions", in: *A symposium on the dingo.* Royal Zoological Society of New South Wales).

Corbett L (1983). "Dingo diet in relation to prey availability in arid, sub-monsoonal and tropical habitats in northern Territory", in: *Australian vertebrate pest control conference.*).

Corbett, L., and Newsome, A. (1975). "Dingo society and its maintenance a preliminary analysis," in *The wild canids. Their systematics, behavioral ecology and evolution,* ed. M.W. Fox.), 369-379.

Corbett, L.K. (1974a). Dingoes feral dogs and cross breeds in Victoria. *Australian Mammalogy* 1(2/3)**,** 303-304.

Corbett, L.K. (1974b). "Part II. The identification of dingoes, feral dogs and hybrids," in *Contributions to the biology of dingoes (Carnivora: canidae) in Victoria*.), 19-44.

Corbett, L.K. (1974c). "Part III. Aspects of dingo biology," in *Contributions to the biology of dingoes (Carnivora: canidae) in Victoria*.), 45-118.

Corbett, L.K. (1988). Social dynamics of a captive dingo pack: Population regulation by dominant female infanticide. *Ethology* 78(3)**,** 177-198. doi: 10.1111/j.1439-0310.1988.tb00229.x.

Corbett, L.K. (1989). Assessing the diet of dingoes from feces: a comparison of 3 methods. *Journal of Wildlife Management* 53(2)**,** 343-346. doi: 10.2307/3801135.

Corbett, L.K. (1998). "Management of dingoes on Fraser Island". (Winnellie, NT.: ERA Environmental Services).

Corbett, L.K. (2009). "Audit of Fraser Island dingo management strategy", (ed.) P. Queensland, Wildlife, Service. (Brisbane: L.K. Corbett).

Corbett, L.K., and Newsome, A.E. (1987). The feeding ecology of the dingo. 3. Dietary relationships with widely fluctuating prey populations in arid Australia: an hypothesis of alternation of predation. *Oecologia (Berlin)* 74(2)**,** 215-227. doi: 10.1007/bf00379362.

Cupples, J.B., Crowther, M.S., Story, G., and Letnic, M. (2011). Dietary overlap and prey selectivity among sympatric carnivores: could dingoes suppress foxes through competition for prey? *Journal of Mammalogy* 92(3)**,** 590-600.

Davis, N.E., Forsyth, D.M., Triggs, B., Pascoe, C., Benshemesh, J., Robley, A., et al. (2015). Interspecific and geographic variation in the diets of sympatric carnivores: dingoes/wild dogs and red foxes in South-Eastern Australia. *PLoS ONE* 10(3)**,** e0120975.

Department of Natural Resources, E., the Arts and Sport of Northern Territory (2014). "Understanding dingoes across the NT benefits for public safety, conservation and management. Draft".).

Doherty, T.S. (2015a). "Chapter 3. Overlap in the diet and habitat use of feral cats and dingoes at a semi-arid rageland site," in *Ecology of feral cats Felis catus and their prey in relation to shrubland fire regimes*. Edith Cowan University, Research Online, Perth, Western Australia), 30-57.

Doherty, T.S. (2015b). "Chapter 5. A game of cat-and-mouse: microhabitat inflences rodent foraging in recently burnt, but not long unburnt shrublands," in *Ecology of feral cats Felis catus and their prey in relation to shrubland fire regimes*. Edith Cowan University, Research Online, Perth, Western Australia), 79-93.

Doherty, T.S. (2015c). Dietary overlap between sympatric dingoes and feral cats at a semiarid rangeland site in Western Australia. *Australian Mammalogy* 37(2)**,** 219-224.

Durie, P.H., and Riek, R.F. (1952). The role of the dingo and wallaby in the infestation ot cattle with hydatids (Echinococcus granulosus (Batsch,1786)Rudolphi, 1805) in Queensland. *Australian Veterinary Journal* 28**,** 249-254. doi: 10.1111/j.1751-0813.1952.tb13436.x.

Dwyer, P., Hockings, M., and Willmer, J. (1979). Mammals of cooloola and beerwah queensland Australia. *Proceedings of the Royal Society of Queensland* (90)**,** 65-84.

Edwards, G.P., de Preu, N., Crealy, I.V., and Shakeshaft, B.J. (2002a). Habitat selection by feral cats and dingoes in a semi-arid woodland environment in central Australia. *Austral Ecology* 27(1)**,** 26-31. doi: 10.1046/j.1442-9993.2002.01156.x.

Edwards, G.P., de Preu, N.D., Shakeshaft, B.J., and Crealy, I.V. (2000). An evaluation of two methods of assessing feral cat and dingo abundance in central Australia. *Wildlife Research* 27(2)**,** 143-149. doi: http://dx.doi.org/10.1071/WR98067.

Edwards, G.P., Dobbie, W., and Berman, D.M. (2002b). Population trends in European rabbits and other wildlife of central Australia in the wake of rabbit haemorrhagic disease. *Wildlife Research* 29(6)**,** 557-565. doi: 10.1071/wr00097.

Edwards, G.P., Dobbie, W., and Berman, D.M. (2002c). Warren ripping: Its impacts on European rabbits and other wildlife of central Australia amid the establishment of rabbit haemorrhagic disease. *Wildlife Research* 29(6)**,** 567-575. doi: 10.1071/wr00098.

Eldridge DT, and Bryan R (1995). "Dingo questionnaire survey June-November 1995", (ed.) Unpublished report to Parks and Wildlife Commission. (Northern Territory, Darwin).

Eldridge S.R., Bird P.L., Brook A., Campbell G., Miller H.A., Read J.L., et al. (2016). "The effect of wild dog control on cattle production and biodiversity in the arid zone of South Australia.". South Australian Arid Lands Natural Resources Management Board).

Eldridge, S.R., Berman, D.M., and Walsh, B. (2000). Field evaluation of four 1080 baits for dingo control. *Wildlife Research* 27(5)**,** 495-500. doi: 10.1071/wr99037.

Elledge, A.E., Allen, L.R., Carlsson, B.-L., Wilton, A.N., and Leung, L.K.P. (2008). An evaluation of genetic analyses, skull morphology and visual appearance for assessing dingo purity: implications for dingo conservation. *Wildlife Research* 35(8)**,** 812-820.

Fleming, P.J.S. (1996a). "4. The efficacy of aerial baiting with 1080-impregated baits," in *Aspects of the management of wild dogs (Canis familiaris) in north-eastern New South Wales*. University of New England), 73-84.

Fleming, P.J.S. (1996b). "5. Effectiveness of the buffer zone and repopulation after baiting," in *Aspects of the management of wild dogs (Canis familiaris) in north-eastern New South Wales*. University of New England), 85-95.

Fleming, P.J.S. (1996c). Ground-placed baits for the control of wild dogs: Evaluation of a replacement-baiting strategy in north-eastern New South Wales. *Wildlife Research* 23(6)**,** 729-740. doi: 10.1071/wr9960729.

Fleming, P.J.S., and Korn, T.J. (1989). Predation of livestock by wild dogs in eastern New South Wales. *Australian Rangeland Journal* 11(2)**,** 61-66.

Fleming, P.J.S., Thompson, J.A., and Nicol, H.I. (1996). Indices for measuring the efficacy of aerial baiting for wild dog control in north-eastern New South Wales. *Wildlife Research* 23(6)**,** 665-674. doi: 10.1071/wr9960665.

Forsyth, D.M., Woodford, L., Moloney, P.D., Hampton, J.O., Woolnough, A.P., and Tucker, M. (2014). How does a carnivore guild utilise a substantial but unpredictable anthropogenic food source? Scavenging on hunter-shot ungulate carcasses by wild dogs/dingoes, red foxes and feral cats in South-Eastern Australia revealed by camera traps. *PLoS ONE* 9(6)**,** e97937.

Foulkes, J.N. (2001). *The ecology and management of the common brushtail possum Trichosurus vulpecula in Central Australia.* thesis - PhD University of Canberra.

Frank, A.S.K., Johnson, C.N., Potts, J.M., Fisher, A., Lawes, M.J., Woinarski, J.C.Z., et al. (2014). Experimental evidence that feral cats cause local extirpation of small mammals in Australia's tropical savannas. *Journal of Applied Ecology* 51(6)**,** 1486-1493.

Glen, A.S., and Dickman, C.R. (2008). Niche overlap between marsupial and eutherian carnivores: does competition threaten the endangered spotted-tailed quoll? *Journal of Applied Ecology* 45(2)**,** 700-707. doi: 10.1111/j.1365-2664.2007.01449.x.

Glen, A.S., Fay, A.R., and Dickman, C.R. (2006). Diets of sympatric red foxes *Vulpes vulpes* and wild dogs *Canis lupus* in the northern rivers region, New South Wales. *Australian Mammalogy* 28(1)**,** 101-104.

Glen, A.S., Pennay, M., Dickman, C.R., Wintle, B.A., and Firestone, K.B. (2011). Diets of sympatric native and introduced carnivores in the Barrington Tops, eastern Australia. *Austral Ecology* 36(3)**,** 290-296. doi: 10.1111/j.1442-9993.2010.02149.x.

Glen, A.S., and Short, J. (2000). The control of dingoes in New South Wales in the period 1883-1930 and its likely impact on their distribution and abundance. *Australian Zoologist* 31(3)**,** 432-442.

Goldingay, R.L., and Whelan, R.J. (1997). Powerline easements: Do they promote edge effects in eucalypt forest for small mammals? *Wildlife Research* 24(6)**,** 737-744.

Gonzalez, T., Allen, L., and Queensland. Department of Natural Resources. Robert Wicks Research, C. (2000). "Movement of dingoes from Shoalwater Bay training area : final report". (Toowoomba, Qld.: Department of Natural Resources).

Gordon, C.E. (2015a). "Chapter 2: Shrub encroachment in an arid landscape is linked to extirpation of a top-predator," in *Dingo (Canis dingo) extirpation and associated trophic restructuring as a mechanism influencing shrub encroachment in arid Australia*.), 38-89.

Gordon, C.E. (2015b). "Chapter 3: Evidence that a top predator benefits a ground-nesting bird by suppressing the abundance of an invasive mesopredator," in *Dingo (Canis dingo) extirpation and associated trophic restructuring as a mechanism influencing shrub encroachment in arid Australia*.), 90-119.

Gordon, C.E. (2015c). "Chapter 6: Dingo (*Canis dingo*) extirpation initiates a trophic cascade ultimately leading to shrub encroachment," in *Dingo (Canis dingo) extirpation and associated trophic restructuring as a mechanism influencing shrub encroachment in arid Australia*.), 179-223.

Gordon, C.E., Feit, A., Grueber, J., and Letnic, M. (2015). Mesopredator suppression by an apex predator alleviates the risk of predation perceived by small prey. *Proceedings of the Royal Society Biological Sciences Series B* 282(1802)**,** 20142870.

Greenville, A.C., Wardle, G.M., Tamayo, B., and Dickman, C.R. (2014). Bottom-up and top-down processes interact to modify intraguild interactions in resource-pulse environments. *Oecologia (Berlin)* 175(4)**,** 1349-1358.

Harden, R.H. (1985). The ecology of the dingo in north-eastern New South Wales. 1. Movements and home range. *Australian Wildlife Research* 12(1)**,** 25-37.

Hernandez-Santin, L., Goldizen, A.W., and Fisher, D.O. (2016). Introduced predators and habitat structure influence range contraction of an endangered native predator, the northern quoll. *Biological Conservation* 203**,** 160-167. doi: 10.1016/j.biocon.2016.09.023.

Hodge, L. (1954). A word for the Dingo. *Victorian Naturalist* 70**,** 207.

Hudson, R., Rodel, H.G., Elizalde, M.T., Arteaga, L., Kennedy, G.A., and Smith, B.P. (2016). Pattern of nipple use by puppies: A comparison of the dingo (*Canis dingo*) and the domestic dog (*Canis familiaris*). *Journal of Comparative Psychology* 130(3)**,** 269-277. doi: 10.1037/com0000023.

Hunt, R.J., Dall, D.J., and Lapidge, S.J. (2007). Effect of a synthetic lure on site visitation and bait uptake by foxes (*Vulpes vulpes*) and wild dogs (*Canis lupus dingo, Canis lupus familiaris*). *Wildlife Research* 34(6)**,** 461-466. doi: http://dx.doi.org/10.1071/WR05110.

Jarman, P.J., and Wright, S.M. (1993). Macropod studies at Wallaby Creek: IX. Exposure and responses of eastern grey kangaroos to dingoes. *Wildlife Research* 20(6)**,** 833-843. doi: 10.1071/wr9930833.

Jenkins, D.J., Fleming, P.J., Cathles, H., and Wilton, A. (2000). The integrated management of wild dogs in south eastern New South Wales, the Australian Capital Territory and north eastern Victoria. *Asian-Australasian Journal of Animal Sciences* 13(Suppl.)**,** 480.

Jones, E. (1990). Physical characteristics and taxonomic status of wild canids, *Canis familiaris*, from the Eastern Highlands of Victoria. *Australian Wildlife Research* 17(1)**,** 69-81.

Jones, E., and Stevens, P.L. (1988). Reproduction in wild canids, *Canis familiaris*, from the eastern highlands of Victoria. *Australian Wildlife Research* 15(4)**,** 385-394.

Jones, N., and Johnson, G.A. (1997). The long-footed potoroo in the north-eastern Victorian alps: 1995-96 survey report. *Flora & Fauna Technical Report* 147**,** i-v, 1-24.

Kennedy, M., Phillips, B.L., Legge, S., Murphy, S.A., and Faulkner, R.A. (2012). Do dingoes suppress the activity of feral cats in northern Australia? *Austral Ecology* 37(1)**,** 134-139.

Koertner, G., and Watson, P. (2005). The immediate impact of 1080 aerial baiting to control wild dogs on a spotted-tailed quoll population. *Wildlife Research* 32(8)**,** 673-680. doi: 10.1071/wr05014.

Krefft, G. (1862). *On the vertebrata of the Lower Murray and Darling.*

Lawrance, K., and Higginbottom, K. (2002). "Behavioural responses of dingoes to tourists on Fraser Island", in: *Wildlife tourism research report series.* (Gold Coast, Qld.: Cooperative Research Centre for Sustainable Tourism).

Leahy, L., Legge, S.M., Tuft, K., McGregor, H.W., Barmuta, L.A., Jones, M.E., et al. (2015). Amplified predation after fire suppresses rodent populations in Australia's tropical savannas. *Wildlife Research* 42(8)**,** 705-716.

Leo, V., Biological, Earth & Environmental Sciences, Faculty of Science, UNSW (2016a). "2. Lethal control of an apex predator is linked to restructuring of tropical savannah ecosystems via multiple ecological cascade pathways," in *Dingo (Canis dingo) extirpation and associated trophic restructuring as a mechanism influencing direct and indirect effects on mammal assemblages in Australia*. University of New South Wales. Biological, Earth & Environmental Sciences), 14-43.

Leo, V., Biological, Earth & Environmental Sciences, Faculty of Science, UNSW (2016b). "3. Lethal control of the dingo exacerbates native rodent declines in the northern tropics of Australia," in *Dingo (Canis dingo) extirpation and associated trophic restructuring as a mechanism influencing direct and indirect effects on mammal assemblages in Australia*. University of New South Wales. Biological, Earth & Environmental Sciences), 44-62.

Leo, V., Biological, Earth & Environmental Sciences, Faculty of Science, UNSW (2016c). "4. Red kangaroos alternate vigilance behaviour in response to predation risk from red foxes and dingoes," in *Dingo (Canis dingo) extirpation and associated trophic restructuring as a mechanism influencing direct and indirect effects on mammal assemblages in Australia*. University of New South Wales. Biological, Earth & Environmental Sciences), 63-85.

Letnic, M., and Crowther, M.S. (2013). Patterns in the abundance of kangaroo populations in arid Australia are consistent with the exploitation ecosystems hypothesis. *Oikos* 122(5)**,** 761-769.

Letnic, M., Crowther, M.S., and Koch, F. (2009a). Does a top-predator provide an endangered rodent with refuge from an invasive mesopredator? *Animal Conservation* 12(4)**,** 302-312.

Letnic, M., and Dworjanyn, S.A. (2011). Does a top predator reduce the predatory impact of an invasive mesopredator on an endangered rodent? *Ecography* 34(5)**,** 827-835. doi: 10.1111/j.1600-0587.2010.06516.x.

Letnic, M., and Koch, F. (2010). Are dingoes a trophic regulator in arid Australia? A comparison of mammal communities on either side of the dingo fence. *Austral Ecology* 35(2)**,** 167-175.

Letnic, M., Koch, F., Gordon, C., Crowther, M.S., and Dickman, C.R. (2009b). Keystone effects of an alien top-predator stem extinctions of native mammals. *Proceedings of the Royal Society Biological Sciences Series B* 276(1671)**,** 3249-3256.

Lundiejenkins, G., Corbett, L.K., and Phillips, C.M. (1993). Ecology of the rufous hare-wallaby, lagorchestes-hirsutus gould (marsupialia, macropodidae), in the tanami desert, northern-territory .3. Interactions with introduced mammal species. *Wildlife Research* 20(4)**,** 495-511. doi: 10.1071/wr9930495.

Lunney, D., Law, B., and Rummery, C. (1996). Contrast between the visible abundance of the brush-tailed rock-wallaby, *Petrogale penicillata*, and its rarity in fox and dog scats in the gorges east of Armidale, New South Wales. *Wildlife Research* 23(3)**,** 373-380. doi: 10.1071/wr9960373.

Lunney, D., Triggs, B., Eby, P., and Ashby, E. (1990). Analysis of scats of dogs Canis familiaris and foxes Vulpes vulpes (Canidae: Carnivora) in coastal forests near Bega, New South Wales. *Australian Wildlife Research* 17(1)**,** 61-68.

Mahon, P.S., Banks, P.B., and Dickman, C.R. (1998). Population indices for wild carnivores: a critical study in sand-dune habitat, south-western Queensland. *Wildlife Research* 25(1)**,** 11-22. doi: 10.1071/wr97007.

Marks, C.A., Allen, L., Gigliotti, F., Busana, F., Gonzalez, T., Lindeman, M., et al. (2004). Evaluation of the tranquilliser trap device (TTD) for improving the humaneness of dingo trapping. *Animal Welfare* 13(4)**,** 393-399.

Marsack, P., and Campbell, G. (1990). Feeding behaviour and diet of dingoes in the Nullarbor region, Western Australia. *Australian Wildlife Research* 17(4)**,** 349-357.

May, S.A. (2001a). "4. Comparison of techniques to survey feral predators in the South-East forests of New South Wales," in *Aspects of the ecology of the cat, fox and dog in the south-east forests of NSW : their potential impact on native species in forests managed for the production of timber*.), 86-147.

May, S.A. (2001b). "5. The use of roads, edges and remote areas by feral predators in the south-east forests of New South Wales," in *Aspects of the ecology of the cat, fox and dog in the south-east forests of NSW : their potential impact on native species in forests managed for the production of timber*.), 148-171.

May, S.A. (2001c). "6. The diet of the fox and dog in the eucalypt forests of South-East New South Wales," in *Aspects of the ecology of the cat, fox and dog in the south-east forests of NSW : their potential impact on native species in forests managed for the production of timber*.), 172-209.

May, S.A. (2001d). "7. The relationship between the abundance of the fox and dog and habitat attributes," in *Aspects of the ecology of the cat, fox and dog in the south-east forests of NSW : their potential impact on native species in forests managed for the production of timber*.), 210-219.

McBride, G. (2007). *An exploratory analysis of landscape-level effects on wild dog home ranges and core areas : a case study at Kosciuszko National Park, and Bago and Maragle State Forest.* Thesis (M Env Sc) -- Australian National University, 2007. .

McLlroy, J.C., Copper, R.J., Giffor, E.J., Green, B.F., and Newgrain, K.W. (1986). The effect on wild dogs *canis familiaris familiaris* of 1080 poisoning campaigns in Kosciusko national park New South Wales Australia. *Australian Wildlife Research* 13(4)**,** 535-544.

McNeill, A., Leung, L., Goullet, M., Gentle, M., and Allen, B. (2016). Dingoes at the doorstep: Home range sizes and activity patterns of dingoes and other wild dogs around urban areas of north-eastern Australia  *Animals* 6(8).

Meek, P.D., and Brown, S.C. (2016). It’s a dog eat dog world: observations of dingo (*Canis familiaris*) cannibalism. *Australian Mammalogy***,** -. doi: http://dx.doi.org/10.1071/AM16018.

Meek, P.D., and Triggs, B. (1998). The food of foxes, dogs and cats on two peninsulas in Jervis Bay, New South Wales. *Proceedings of the Linnean Society of New South Wales* 120**,** 117-127.

Mitchell, B.D., and Banks, P.B. (2005). Do wild dogs exclude foxes? Evidence for competition from dietary and spatial overlaps. *Austral Ecology* 30(5)**,** 581-591. doi: 10.1111/j.1442-9993.2005.01473.x.

Mitchell, J., Merrell, P., Allen, L., Queensland. Stock, R., and Rural Lands Protection, B. (1982). "Vertebrate pests of Queensland". (Brisbane, Qld: Stock Routes and Rural Lands Protection Board).

Morrant, D.S. (2015a). "Chapter 2: Movement ecology of dingoes in a forest-cropland interface: Implications for native faune," in *Potential for spillover predation on native fauna by dingoes in peri-urban and agricultural landscapes in Australia's lowland Wet Tropics*. James Cook University), 13-50.

Morrant, D.S. (2015b). "Chapter 3: Does predation by dingoes in te lowland wet tropics threaten biodiversity conservation?," in *Potential for spillover predation on native fauna by dingoes in peri-urban and agricultural landscapes in Australia's lowland Wet Tropics*. James Cook University), 51-84.

Morrant, D.S., Johnson, C.N., Butler, J.R., and Congdon, B.C. (2017). Biodiversity friend or foe: land use by a top predator, the dingo in contested landscapes of the Australian Wet Tropics. *Austral Ecology* 42(3)**,** 252-264.

Moseby, K.E., Neilly, H., Read, J.L., and Crisp, H.A. (2012). Interactions between a top order predator and exotic mesopredators in the Australian rangelands. *International Journal of Ecology***,** 250352, 250351-250315.

Moseby, K.E., Owens, H., Brandle, R., Bice, J.K., and Gates, J. (2006). Variation in population dynamics and movement patterns between two geographically isolated populations of the dusky hopping mouse (Notomys fuscus). *Wildlife Research* 33(3)**,** 223-232. doi: 10.1071/wr05034.

Moseby, K.E., Read, J.L., Paton, D.C., Copley, P., Hill, B.M., and Crisp, H.A. (2011). Predation determines the outcome of 10 reintroduction attempts in arid South Australia. *Biological Conservation* 144(12)**,** 2863-2872.

Newsome, A., and Catling, P. (1979). "Habitat preferences of mammals inhabiting heathlands of temperate, coastal, montane and alpine regions of southeastern Australia," in *Heathlands and Related Shrublands of the World,* ed. R.L. Specht.), 301-316.

Newsome, A.E., Catling, P.C., Cooke, B.D., and Smyth, R. (2001). Two ecological universes separated by the Dingo Barrier fence in semi-arid Australia: Interactions between landscapes, herbivory and carnivory, with and without dingoes. *Rangeland Journal* 23(1)**,** 71-98. doi: 10.1071/rj01015.

Newsome, A.E., Catling, P.C., and Corbett, L.K. (1983a). The feeding ecology of the dingo. 2. Dietary and numerical relationships with fluctuating prey populations in south-eastern Australia. *Australian Journal of Ecology* 8(4)**,** 345-366. doi: 10.1111/j.1442-9993.1983.tb01332.x.

Newsome, A.E., and Corbett, L. (1982). The identity of the dingo. 2. Hybridization with domestic dogs in captivity and in the wild. *Australian Journal of Zoology* 30(2)**,** 365-374. doi: 10.1071/zo9820365.

Newsome, A.E., and Corbett, L.K. (1985). The identity of the dingo. 3. The incidence of dingoes, dogs and hybrids and their coat colours in remote and settled regions of Australia. *Australian Journal of Zoology* 33(3)**,** 363-375. doi: 10.1071/zo9850363.

Newsome, A.E., Corbett, L.K., Catling, P.C., and Burt, R.J. (1983b). The feeding ecology of the dingo 1. Stomach contents from trapping in south-eastern Australia, and the non-target wildlife also caught in dingo traps. *Australian Wildlife Research* 10(3)**,** 477-486.

Newsome, A.E., Corbett, L.K., and Stephens, D.R. (1972). "Assessment of an aerial baiting campaign against dingoes in central Australia", in: *Division of Wildlife Research technical paper ; no. 24.*).

Newsome, T.M., Ballard, G.-A., Crowther, M.S., Fleiveing, P.J.S., and Dickman, C.R. (2014a). Dietary niche overlap of free-roaming dingoes and domestic dogs: the role of human-provided food. *Journal of Mammalogy* 95(2)**,** 392-403.

Newsome, T.M., Ballard, G.-A., Dickman, C.R., Fleming, P.J.S., and Howden, C. (2013a). Anthropogenic resource subsidies determine space use by Australian arid zone dingoes: An improved resource selection modelling approach. *PLoS ONE* 8(5)**,** e63931.

Newsome, T.M., Ballard, G.-A., Dickman, C.R., Fleming, P.J.S., and van de Ven, R. (2013b). Home range, activity and sociality of a top predator, the dingo: a test of the Resource Dispersion Hypothesis. *Ecography* 36(8)**,** 914-925.

Newsome, T.M., Ballard, G.-A., Fleming, P.J.S., van de Ven, R., Story, G.L., and Dickman, C.R. (2014b). Human-resource subsidies alter the dietary preferences of a mammalian top predator. *Oecologia (Berlin)* 175(1)**,** 139-150.

Newsome, T.M., and Science, U.o.S.F.o. (2011a). "Chapter 3. Diet selectivity of the dingo and anthropogenic food subsidies in the Tanami Desert, Australia," in *Ecology of the dingo (Canis lupus dingo) in the Tanami Desert in relation to human-resouce subsidies*. University of Sydney. Faculty of Science ), 28-80.

Newsome, T.M., and Science, U.o.S.F.o. (2011b). "Chapter 5. Resource selection by dingoes in the Tanami Desert, Australia," in *Ecology of the dingo (Canis lupus dingo) in the Tanami Desert in relation to human-resouce subsidies*. University of Sydney. Faculty of Science ), 123-170.

Newsome, T.M., Stephens, D., Ballard, G.-A., Dickman, C.R., and Fleming, P.J.S. (2013c). Genetic profile of dingoes (*Canis lupus dingo*) and free-roaming domestic dogs (*C. l. familiaris*) in the Tanami Desert, Australia. *Wildlife Research* 40(3)**,** 196-206.

Palmer, R. (2012). Diet of the dingo (*Canis lupus dingo*) from the desert uplands of North Queensland. *Queensland Naturalist* 50(1-3)**,** 23-26.

Paltridge, R. (2002). The diets of cats, foxes and dingoes in relation to prey availability in the Tanami Desert, Northern Territory. *Wildlife Research* 29(4)**,** 389-403. doi: 10.1071/wr00010.

Paltridge, R.M. (2005). *Predator-prey interactions in the spinifex grasslands of central Australia.* School of Biological Sciences - Faculty of Science.

Pascoe, J.H., Mulley, R.C., Spencer, R., and Chapple, R. (2011). Diet analysis of mammals, raptors and reptiles in a complex predator assemblage in the Blue Mountains, eastern Australia. *Australian Journal of Zoology* 59(5)**,** 295-301.

Pascoe, J.H., and Sciences, U.o.W.S.S.o.N. (2011a). "Chapter 3: Habitat influences on apex predators and prey," in *Apex predators in the Greater Blue Mountains World Heritage Area*.), 52-83.

Pascoe, J.H., and Sciences, U.o.W.S.S.o.N. (2011b). "Chapter 5: Activity trends of apex predators and their prey," in *Apex predators in the Greater Blue Mountains World Heritage Area*.), 84-111.

Pavey, C.R., Eldridge, S.R., and Heywood, M. (2008). Population dynamics and prey selection of native and introduced predators during a rodent outbreak in arid Australia. *Journal of Mammalogy* 89(3)**,** 674-683. doi: 10.1644/07-mamm-a-168r.1.

Pople, A.R., Grigg, G.C., Cairns, S.C., Beard, L.A., and Alexander, P. (2000). Trends in the numbers of red kangaroos and emus on either side of the South Australian dingo fence: Evidence for predator regulation? *Wildlife Research* 27(3)**,** 269-276. doi: 10.1071/wr99030.

Purcell, B.V. (2008a). "Chapter four: Intraspecific variations in spatial organisation, movement and activity," in *Order in the pack : ecology of Canis lupus dingo in the southern Greater Blue Mountains World Heritage Area*. University of Western Sydney. School of Natural Sciences), 176-237.

Purcell, B.V. (2008b). "Chapter three: Interactions between sympatric competitors and their prey," in *Order in the pack : ecology of Canis lupus dingo in the southern Greater Blue Mountains World Heritage Area*. University of Western Sydney. School of Natural Sciences), 129-175.

Purcell, B.V. (2008c). "Chapter two: What is a “pure” dingo?," in *Order in the pack : ecology of Canis lupus dingo in the southern Greater Blue Mountains World Heritage Area*. University of Western Sydney. School of Natural Sciences), 63-128.

Purcell BV, Mulley R, Close R, and Fleming P (2006). "Use of GPS collars for tracking wild dogs", in: *Queensland Pest Animal Symposium Proceedings.* (Toowoomba).

Queensland, P., and Wildlife, S. (2010). "Fraser Island dingo population study : interim report stage 1". (Brisbane: Department of Environment and Resource Management).

Queensland. Environmental Protection, A. (2001). "Risk assessment : risk to humans posed by the dingo population on Fraser Island". (Brisbane, Qld.: Environmental Protection Agency).

Radford, C.G., Letnic, M., Fillios, M., and Crowther, M.S. (2012). An assessment of the taxonomic status of wild canids in south-eastern New South Wales: phenotypic variation in dingoes. *Australian Journal of Zoology* 60(2)**,** 73-80.

Read, J., and Eldridge, S. (2010). An optimised rapid detection technique for simultaneously monitoring activity of rabbits, cats, foxes and dingoes in the rangelands. *The Rangeland Journal* 32(4)**,** 389-394. doi: http://dx.doi.org/10.1071/RJ09018.

Read, J.L., Bengsen, A.J., Meek, P.D., and Moseby, K.E. (2015). How to snap your cat: optimum lures and their placement for attracting mammalian predators in arid Australia. *Wildlife Research* 42(1)**,** 1-12. doi: 10.1071/wr14193.

Reynolds, J.E., Dortch, J., and Balme, J. (2016). Dingo scat-bone ‘signature patterns’: an actualistic study and comparison of wild and captive scat-bone assemblages and interpretation of bone fragments from Witchcliffe Rock Shelter, south western Australia. *Australian Archaeology* 82(3)**,** 1-14. doi: 10.1080/03122417.2016.1240136.

Robertshaw, J., and Harden, R. (1986). The ecology of the dingo in northeastern New South Wales 4. Prey selection by dingoes, and its effect on the major prey species, the swamp wallaby, *Wallabia bicolor* (desmarest). *Wildlife Research* 13(2)**,** 141-163. doi: http://dx.doi.org/10.1071/WR9860141.

Robertshaw, J.D., and Harden, R.H. (1985a). The ecology of the dingo *Canis familiaris dingo* in northeastern New South Wales Australia 2. Diet. *Australian Wildlife Research* 12(1)**,** 39-50.

Robertshaw, J.D., and Harden, R.H. (1985b). The ecology of the dingo in north-eastern New South Wales. 2. Diet. *Australian Wildlife Research* 12(1)**,** 39-50.

Robley, A., Gormley, A., Forsyth, D.M., Wilton, A.N., and Stephens, D. (2010). Movements and habitat selection by wild dogs in eastern Victoria. *Australian Mammalogy* 32(1)**,** 23-32.

Robley, A., Woodford, L., Lee, P., Kingston, V., Peters, W., Klippell, D., et al. (2009). Assessing the effectiveness of ground-based baiting for the control of wild dogs. *Arthur Rylah Institute for Environmental Research Technical Report Series* 193**,** i-ii, 1-21.

Schroeder, T., Lewis, M.M., Kilpatrick, A.D., and Moseby, K.E. (2015). Dingo interactions with exotic mesopredators: spatiotemporal dynamics in an Australian arid-zone study. *Wildlife Research* 42(6)**,** 529-539. doi: 10.1071/wr15104.

Shepherd, N.C. (1981). Predation of red kangaroos, Macropus rufus, by the dingo, *Canis familiaris dingo* (Blumenbach), in north-western New South Wales. *Australian Wildlife Research* 8(2)**,** 255-262.

Somaweera, R., Webb, J.K., and Shine, R. (2011). It's a dog-eat-croc world: dingo predation on the nests of freshwater crocodiles in tropical Australia. *Ecological Research* 26(5)**,** 957-967.

Southgate, R., Palmer, C., Adams, M., Masters, P., Triggs, B., and Woinarski, J. (1996). Population and habitat characteristics of the golden bandicoot (*Isoodon auratus*) on Marchinbar Island, Northern Territory. *Wildlife Research* 23(6)**,** 647-664. doi: 10.1071/wr9960647.

Southgate, R., Paltridge, R., Masters, P., and Ostendorf, B. (2007). Modelling introduced predator and herbivore distribution in the Tanami Desert, Australia. *Journal of Arid Environments* 68(3)**,** 438-464. doi: 10.1016/j.jaridenv.2006.06.006.

Sparkes, J., Ballard, G., Fleming, P.J.S., Ven, R.v.d., and Kortner, G. (2016). Contact rates of wild-living and domestic dog populations in Australia: a new approach. *Oecologia* 182(4)**,** 1007-1018.

Spencer, E.E., Crowther, M.S., and Dickman, C.R. (2014). Diet and prey selectivity of three species of sympatric mammalian predators in central Australia. *Journal of Mammalogy* 95(6)**,** 1278-1288.

Stephen R. Eldridge, Bernie J. Shakeshaft, and Nano, T.J. (2002). "The impact of wild dog control on cattle, native and introduced herbivores and introduced predators in central Australia", in: *Parks & Wildlife Commission of the Northern Territory.* (ed.) C. Unpublished report to the Bureau of Rural Sciences.).

Stephens, D. (2011a). "Chapter 2: Comparison of non-invasive DNA sample sources for monitoring wild dogs in Australia," in *The molecular ecology of Australian wild dogs : hybridisation, gene flow and genetic structure at multiple geographic scales*. University of Western Australia. School of Animal Biology), 12-32.

Stephens, D. (2011b). "Chapter 4: Human developments impact upon the ecology of dingoes in the Tanami Desert, Australia," in *The molecular ecology of Australian wild dogs : hybridisation, gene flow and genetic structure at multiple geographic scales*. University of Western Australia. School of Animal Biology), 55-67.

Stephens, D., Wilton, A.N., Fleming, P.J.S., and Berry, O. (2015). Death by sex in an Australian icon: a continent-wide survey reveals extensive hybridization between dingoes and domestic dogs. *Molecular Ecology* 24(22)**,** 5643-5656.

Thomson, P.C. (1986). The effectiveness of aerial baiting for the control of dingoes *Canis familiaris dingo* in north-western Australia. *Australian Wildlife Research* 13(2)**,** 165-176.

Thomson, P.C. (1992a). The behavioral ecology of dingoes in north-western Australia 1. The Fortescue River study area and details of captured dingoes. *Wildlife Research* 19(5)**,** 509-518. doi: 10.1071/wr9920509.

Thomson, P.C. (1992b). The behavioral ecology of dingoes in north-western Australia 2. Activity patterns, breeding-season and pup rearing. *Wildlife Research* 19(5)**,** 519-530. doi: 10.1071/wr9920519.

Thomson, P.C. (1992c). The behavioral ecology of dingoes in north-western Australia 4. Social and spatial-organization, and movements. *Wildlife Research* 19(5)**,** 543-563. doi: 10.1071/wr9920543.

Thomson, P.C. (1992d). The behavioural ecology of dingoes in north-western Australia: III. Hunting and feeding behaviour, and diet. *Wildlife Research* 19(5)**,** 531-541. doi: 10.1071/wr9920531.

Thomson, P.C., Rose, K., and Kok, N.E. (1992a). The behavioral ecology of dingoes in north-western Australia 5. Population-dynamics and variation in the social system. *Wildlife Research* 19(5)**,** 565-584. doi: 10.1071/wr9920565.

Thomson, P.C., Rose, K., and Kok, N.E. (1992b). The behavioral ecology of dingoes in north-western Australia 6. Temporary extraterritorial movements and dispersal. *Wildlife Research* 19(5)**,** 585-595. doi: 10.1071/wr9920585.

Tierney, P.J., and Strong, K. (1987). "The use of 1080 to control dingoes on Taunton Scientific Reserve", in: *Management report (Queensland. National Parks and Wildlife Service) ; 1987.* (Brisbane, Qld.: Queensland National Parks and Wildlife Service).

Tomlinson, A.R. (1954). Aerial baiting against wild dogs and foxes in Western Australia. *Journal of the Department of Agriculture* 3**,** 37-49.

Tomlinson, A.R., and Blair, C.K. (1952). Wild dog investigations. The Warburton Ranges Area long dash July 22 to August 29, 1952. *Journal of Agriculture of Western Australia* 1((6))**,** 883-893.

Triggs, B., Brunner, H., and Cullen, J.M. (1984). The food of fox, dog and cat in Croajingalong National Park, south-eastern Victoria. *Australian Wildlife Research* 11(3)**,** 491-499.

Twigg, L.E., Eldridge, S.R., Edwards, G.P., Shakeshaft, B.J., dePreu, N.D., and Adams, N. (2000). The longevity and efficacy of 1080 meat baits used for dingo control in central Australia. *Wildlife Research* 27(5)**,** 473-481. doi: 10.1071/wr99044.

Vernes, K. (2000). Immediate effects of fire on survivorship of the northern bettong (*Bettongia tropica*): an endangered Australian marsupial. *Biological Conservation* 96(3)**,** 305-309. doi: 10.1016/s0006-3207(00)00086-0.

Vernes, K., Dennis, A., and Winter, J. (2001). Mammalian diet and broad hunting strategy of the dingo (*Canis familiaris dingo*) in the wet tropical rain forests of northeastern Australia. *Biotropica* 33(2)**,** 339-345. doi: 10.1111/j.1744-7429.2001.tb00185.x.

Wallach, A.D. (2011). *Reviving ecological functioning through dingo restoration.* Thesis (Ph.D.), University of Adelaide, School of Earth and Environmental Sciences.

Wallach, A.D., Johnson, C.N., Ritchie, E.G., and O'Neill, A.J. (2010). Predator control promotes invasive dominated ecological states. *Ecology Letters* 13(8)**,** 1008-1018.

Wallach, A.D., Murray, B.R., and O'Neill, A.J. (2009a). Can threatened species survive where the top predator is absent? *Biological Conservation* 142(1)**,** 43-52. doi: 10.1016/j.biocon.2008.09.021.

Wallach, A.D., and O'Neill, A.J. (2008). "Persistence of endangered species: is the dingo the key?". (South Australia. Department of Environment and Natural Resources).

Wallach, A.D., and O'Neill, A.J. (2009). Threatened species indicate hot-spots of top-down regulation. *Animal Biodiversity and Conservation* 32(2)**,** 127-133.

Wallach, A.D., Ritchie, E.G., Read, J., and O'Neill, A.J. (2009b). More than mere numbers: the impact of lethal control on the social stability of a top-order predator. *PLoS ONE* 4(9)**,** e6861, 6861-6868.

Wang, Y., and Fisher, D.O. (2012). Dingoes affect activity of feral cats, but do not exclude them from the habitat of an endangered macropod. *Wildlife Research* 39(7)**,** 611-620.

West, P., and Saunders, G. (2007). "Pest animal survey: 2004-06. A review of the distribution, impacts and control of invasive animals throughout NSW and the ACT", in: *Pest animal survey: 2004-06. A review of the distribution, impacts and control of invasive animals throughout NSW and the ACT.*: NSW Department of Primary Industries).

Whitehouse, S. (1978). "Dingo movements in rangeland areas of Western Australia", in: *Proceedings of the first international rangeland congress, Denver, Colorado, USA, August 14-18, 1978 [Hyder, D.N. (Editor)].*).

Whitehouse, S.J.O. (1977a). The diet of the dingo in Western Australia. *Australian Wildlife Research* 4(2)**,** 145-150.

Whitehouse, S.J.O. (1977b). Movements of dingoes in Western Australia. *Journal of Wildlife Management* 41(3)**,** 575-576,illust. doi: 10.2307/3800532.

Wilton, A.N. (2001). "DNA methods of assessing dingo purity", in: *A Symposium on the Dingo.* (eds.) C.R. Dickman & D. Lunney. (Mosman NSW).

Woodall, P.F. (1983). Distribution and population dynamics of dingoes (*Canis familiaris*) and feral pigs (*Sus scrofa*) in Queensland, 1945-1976. *Journal of Applied Ecology* 20(1)**,** 85-95. doi: 10.2307/2403377.

Wysong, M.L. (2016a). "2. On the right track: Understanding the influence of roads and lures improves camera trap surveys for predators and prey," in *Predator ecology in the arid rangelands of Western Australia: Spatial interactions and resource competition between an apex predator, the dingo Canis dingo, and an introduced mesopredator, the feral cat Felis catus*. The University of Western Australia ), 17-44.

Wysong, M.L. (2016b). "3. The truth about cats and dogs: Assessment of dingo and feral cat diets improves when observer uncertainty is reduced," in *Predator ecology in the arid rangelands of Western Australia: Spatial interactions and resource competition between an apex predator, the dingo Canis dingo, and an introduced mesopredator, the feral cat Felis catus*. The University of Western Australia ), 45-74.

Wysong, M.L. (2016c). "4. Seasonal space use and fine-scale habitat selection of sympatric predators in a semi-arid landscape," in *Predator ecology in the arid rangelands of Western Australia: Spatial interactions and resource competition between an apex predator, the dingo Canis dingo, and an introduced mesopredator, the feral cat Felis catus*. The University of Western Australia ), 75-116.

**Appendix E. Quantitative estimates for home range, density or population size and contacts or interactions**

**Table E1**. Quantitative estimates extracted from 24 studies, captured in a scoping review, investigating home range in Australian dingoes and wild-living dogs

| Study reference | Year of collection | Climate | Type of dog | Type of environment | Dog control | Method data collection | Method data calculation | Specification on estimate | n | Mean estimate  ± SE (km^2^) | Range  (km^2^) |
| --- | --- | --- | --- | --- | --- | --- | --- | --- | --- | --- | --- |
| (Allen et al., 2013b) | 2005-2006 | Subtrop. | Dingo | Urban | NR | GPS tr. | AK 95% |  | 9 | 17.72 | 0.37-100.32 |
|  |  |  |  |  |  |  | AK 50% |  | 2 | NR | 1.89 – 5.62 |
| (Newsome et al., 2013b) | 2008-2010 | Grass. | Dingo | Rural and mine | NR | GPS tr. | MCP 95% |  | 13 | NR | 2 – 2013 |
|  |  |  |  |  |  |  | FK 85% |  | 13 | NR | 0.7 – 999 |
| (Allen, 2012a) | 2008-2009 | Des. | Dingo | Rural | Without control | GPS tr. | NR | Pack area | 4 | ~24 | NR |
| (Robley et al., 2010) | 2007-2008 | Temp. | Dingo and hybrid | Rural | Without control | GPS tr. | MCP 100% |  | 9 | 100.0 | 30.2 – 218.0 |
|  |  |  |  |  |  |  | MCP 95% | Males | 4 | 124.3 ± 56.3 | 25.6 – 68.9 |
|  |  |  |  |  |  |  |  | Females | 5 | 45.2 ± 17.3 | 46.9 – 177.7 |
| (Claridge et al., 2009) | NR | Temp. | Dingo and hybrid | Rural | Without control | GPS tr. | MCP (NR%) |  | 24 | NR | 10.22 – 262.09 |
|  |  |  |  |  |  |  | FK 95% |  | 24 | 99.23 ± 77.76 (SD) | 9.25 – 276.19 |
| (Robley et al., 2009) | 2008-2009 | Temp. | UN | Rural | Without control | GPS tr. | MCP 95% |  | 10 | NR | 17 - 94 |
| (Harden, 1985) | 1970-1974 | Temp. | Dingo | Rural | NR | Radio-tr. | Join all outermost fixes |  | 8 | NR | 4.3 – 54.8 |
| (Thomson, 1992c) | 1975-1978 | Grass. | Dingo | Rural | Without control | Radio-tr. | MCP 100% |  |  |  |  |
|  |  |  |  |  |  |  |  | Male adult | 3 | 134.6 | NR |
|  |  |  |  |  |  |  |  | Male non-adult | 2 | 76.2 | NR |
|  |  |  |  |  |  |  |  | Female adult | 8 | 81.3 | NR |
|  |  |  |  |  |  |  |  | Female non-adult | 3 | 57.8 | NR |
|  |  |  |  |  |  |  |  | Lone | 3 | 253.7 | NR |
|  |  |  |  |  |  |  | MCP 95% |  |  |  |  |
|  |  |  |  |  |  |  |  | Male adult | 3 | 84.8 ± 12.1 | NR |
|  |  |  |  |  |  |  |  | Male non-adult | 2 | 37.0 | 37.0 – 37.1 |
|  |  |  |  |  |  |  |  | Female adult | 8 | 56.0 ± 8.6 | NR |
|  |  |  |  |  |  |  |  | Female non-adult | 3 | 48.8 ± 18.0 | NR |
|  |  |  |  |  |  |  |  | Lone | 3 | 159.6 ± 33.8 | NR |
|  |  |  |  |  |  |  | NR | Pack area | 5 | NR | 44.5 – 113.2 |
| (Thomson et al., 1992a) | 1975-1984 | Grass. | Dingo | Rural | With control | Radio-tr. | Convex polygon 95% | Pack area | 8 | NR | 23.7 – 195.5 |
| (Allen et al., 2014) | 2011-2012 | Des. | UN | Rural | Both control | GPS tr. | MCP 100% | Pre-baiting | 7 | 56.94 | 12.42 – 194.42 |
|  |  |  |  |  |  |  | MCP 100% | Post-baiting | 7 | 32.71 | 16.65 – 78.23 |
|  |  |  |  |  |  |  | AK 90% |  | 7 | 63.52 | 16.57 – 286.33 |
|  |  |  |  |  |  |  | AK 50% |  | 7 | 7.88 | 2.97 – 28.53 |
| (McLlroy et al., 1986) | 1982 | Temp. | UN | Rural | With control | Radio-tr. | Convex Polygon (NR%) |  | 9 | 21.93 ± 6.48 | 2.20 – 54.20 |
| (Whitehouse, 1978) | 1975-1977 | Grass. | Dingo | Rural | NR | Radio-tr. | NR | Pack area | NR | NR | 70 - 125 |
| (Brook, 2013c) | 2011-2012 | Grass. | Dingo | Rural | NR | GPS tr. | FK 95% |  | 10 | 26.98 ± 7.81 | 7.16 – 94.05 |
|  |  |  |  |  |  |  | FK 70.34%* |  | 10 | 7.15 ± 2.37 | 0.87 – 27.23 |
|  |  |  |  |  |  |  | MKDE 95% |  | 8 | 16.49 ± 15.07 | 10.83 – 22.54 |
|  |  |  |  |  |  |  | MDKE 71.84%* |  | 8 | 3.37 ± 0.56 | 1.33 – 6.05 |
|  |  |  |  |  |  |  | MCP 100% |  | 16 | NR | 20.82 – 1790.25 |
|  |  |  |  |  |  |  | MCP 95% |  | 16 | NR | 19.58 – 1496.91 |
|  |  |  |  |  |  |  | MCP 90% |  | 16 | NR | 8.85 – 1445.50 |
|  |  |  |  |  |  |  | MCP 50% |  | 16 | NR | 0.004 – 523.09 |
| (Wysong, 2016c) | 2013-2014 | Des. | Dingo | Rural | Without control | GPS tr. | FK 95% |  | 16 | 681.86 ± 137.76 | 109.71 – 1912.27 |
|  |  |  |  |  |  |  | FK 50% |  | 16 | 140.89 ± 27.20 | 12.50 – 276.97 |
| (Morrant, 2015a) | 2010-2012 | Trop. | UN | Rural and urban | NR | GPS tr. | MCP 100% |  | 9 | 53.5 ± 11.37 | 6.9 – 107.3 |
|  |  |  |  |  |  |  | MCP 95% |  | 9 | 39.7 | 3.4 – 82.5 |
|  |  |  |  |  |  |  | AK 95% |  | 9 | 32.9 | 3.1 – 104.7 |
|  |  |  |  |  |  |  | FK 85% |  | 9 | 13.2 ± 4.32 | 0.5 – 35.7 |
|  |  |  |  |  |  |  | LoCoH 95% |  | 9 | 11.2 ± 2.5 | 1.4 – 22.9 |
|  |  |  |  |  |  |  | MKDE 95% |  | 8 | 17 ± 3.64 | 2.9 – 30.5 |
| (Purcell, 2008a) | 2005-2007 | Temp. | Dingo and hybrid | Rural | With control | GPS tr. | MCP 95% |  | 12 | 37.7 ± 8.8 | 6.67 – 103.8 |
|  |  |  |  |  |  |  | MCP 50% |  | 12 | 7.9 ± 1.8 | 1.37 – 18.91 |
|  |  |  |  |  |  |  | FK 90% |  | 12 | 34.2 ± 8.2 | 4.00 – 86.93 |
|  |  |  |  |  |  |  | FK 50% |  | 12 | 5.9 ± 1.4 | 0.89 – 16.15 |
| (McBride, 2007) | 2001-2005 | Temp. | UN | Rural | Both control | Both GPS tr. and Radio-tr. | MCP 100% |  | 18 | 72.2 | 21.8 – 173.0 |
|  |  |  |  |  |  |  | FK 80% |  | 18 | 23.7 | 3.1 – 46.4 |
|  |  |  |  |  |  |  | FK 50% |  | 18 | 7.4 | 1.5 – 18.2 |
|  |  |  |  |  |  |  | FK 20% |  | 18 | 1.8 | 0.5 – 4.1 |
| (Stephen R. Eldridge et al., 2002) | 2001-2002 | Des. | Dingo | Rural | Without control | GPS tr. | MCP 95% |  | 3 | NR | 116.7 – 272.1 |
| (Baxter and Davies, 2013) | 2011-2012 | Subtrop. | Dingo | Rural | Without control | GPS tr. | MCP 95% |  | 18 | NR | 6.12 – 1067.19 |
|  |  |  |  |  |  |  | FK 95% |  | 18 | NR | 5.47 – 574.59 |
| (McNeill et al., 2016) | 2013-2016 | Subtrop, Trop. | UN | Urban | NR | GPS tr. | AK 90% |  | 35 | 17.47 | 0.53 – 66.02 |
| (Allen et al., 2016b) | 2010 | Grass. | UN | Rural | Without control | GPS tr. | AK 90% |  | 5 | NR | 62.0 – 343.4 |
| (Allen and Byrne, 2008) | 2006-2008 | Des., Grass., Trop. | UN | NR | Both control | GPS tr. | NR |  | NR | NR | NR |
| (Best, 1978) | 1969-1976 | Grass., Des. | Dingo | Rural | NR | Radio-tr. | NR |  | NR | NR | 20 – 30 |
| (Catling, 1978) | 1974-1976 | Temp. | Dingo | Rural | NR | Radio-tr. | NR |  | 9 | NR | 9 – 21.5 |

General abbreviations: NA = Not applicable; NR = Not reported

Climate zone abbreviations: Trop. = Tropical; Subtrop. = Subtropical; Temp. = Temperate; Des. = Desert; Grass. = Grassland

Type of dog abbreviations: UN = Unknown sample

Method data collection abbreviations: GPS tr. = GPS Satellite tracking; Radio-tr. = Radio-tracking with ground or aircraft searches

Abbreviations for ‘Methods of calculation’: MCP = Minimum Convex Polygon; FK = Fixed Kernel; AK = Adaptive Kernel; MKDE = Movement-based Kernel Density; LoCoH = Local Convex Hulls;

The symbol ~ is used when the data is approximated

* Isopleth % is a mean estimate of core area identified separately for each individual

**Table E2**. Quantitative estimates extracted from 14 studies, captured in a scoping review, investigating density or population size of Australian dingoes and wild-living dogs

**A. Density**

| **Study reference** | **Year collection** | **Climate** | **Type of dog** | **Type of environment** | **Dog control** | **Methods data collection** | **Methods data calculation** | **Density estimate (dog/km^2^)** | | |
| --- | --- | --- | --- | --- | --- | --- | --- | --- | --- | --- |
|  |  |  |  |  |  |  |  | **Specification on estimate** | **Estimate (95% CI)** | **Range** |
| (Corbett, 1995) | 1980-1986 | Trop. | Dingo | Rural | NR | Radio-tracking, mark-recapture and sightings, tracks and other fresh signs of dingoes along road transects | Ratio formula which takes into account results from radio-tracking and mark-recapture studies and road transect results from this study. | Adult dingoes | 0.14 | NR |
| (Allen et al., 2015) | 1995-2013 | Subtrop. | Dingo | Rural | With control | GPS tracking, remote camera monitoring data and observational information recorded. | Number of packs multiplied by group sizes, over the study area | Dingoes >10 months old | NR | 0.04 – 0.09 |
|  |  |  |  |  |  |  |  | All dingoes (including pups) | NR | 0.09 – 0.14 |
| (Thomson et al., 1992a) | 1975-1984 | Grass. | Dingo | Rural | With control | Radio-tracking and ground survey | Counts of dingoes in study area | Dingoes ≥ 6 months old Includes periods with and without baiting. | NR | ~ 0.01 – ~0.22 |
| (Pople et al., 2000) | 1978, 1983-1992 | Des. | Dingo | Rural | Both control | Aerial survey | Counts of dingoes in study area | Inside dingo fence | NR | 0 – ~ 0.20 |
|  |  |  |  |  |  |  |  | Outside dingo fence | NR | 0 – ~ 0.70 |
| (McLlroy et al., 1986) | 1982 | Temp. | UN | Rural | With control | Trapping and observations | Counts of observed or trapped dogs in the study area | Minimum known density | 0.18 | NR |
| (Durie and Riek, 1952) | NR | NA | Dingo | Entire state | NA | Data on number of bonuses paid for dingo scalps | Number of bonuses | Minimum known density per shire | NR | 0 to 0.28 – 0.39 |
| (Fleming, 1996a) | 1992-1993 | Temp. | UN | Rural | Both control | Responses to howls simulation and abundance indices before and after baiting. | Ratio using number of individual dogs that responded to howls and abundance indices | Minimum known density | NR | 0.10 – 0.30 |
| (Mitchell et al., 1982) | 1981-1982 | NA | Mixed | Entire state | NA | Interviews conducted on Shire Council and Government agencies personnel. | Approximate density based on signs of presence, animal sightings and group size |  | NR | > 0.03 – 0.10 |

**B. Population size**

| **Study reference** | **Year collection** | **Climate** | **Type of dog** | **Type of environment** | **Dog control** | **Methods data collection** | **Methods data calculation** | **Population size estimate (dogs)** | | |
| --- | --- | --- | --- | --- | --- | --- | --- | --- | --- | --- |
|  |  |  |  |  |  |  |  | **Specification on estimate** | **Estimate (95% CI)** | **Range** |
| (Fleming, 1996c) | 1993 | Temp. | UN | Rural | Both control | Abundance indices at baiting stations and data from the literature | Index-manipulation-index calculation | Minimum number of wild dogs by site, prior to baiting | NR | 18 – 22 |
|  |  |  |  |  |  |  |  | Minimum number of wild dogs by site, after baiting | NR | 4 – 6 |
|  |  |  |  |  |  |  |  | Minimum number of wild dogs, at unbaited site | 35 | NR |
| (Newsome et al., 1972) | 1968 | Grass., Des. | Dingo | Rural | Both control | Examination of tracks during ground surveys | Number of fresh tracks | Number of adults and pups per site: pre-baiting or in unbaited stations | NR | 1 – 20 |
|  |  |  |  |  |  |  |  | Number of adults and pups per site: post-baiting | NR | 0 – 99 |
|  |  |  |  |  |  |  |  | Number of adults and pups in unbaited sites | NR | 8 – 35 |
| (Queensland and Wildlife, 2010) | 2009-2010 | Subtrop. | Dingo | Rural | Without control | Ear tag register | Number of tagged individuals and number of untagged individuals which were sighted |  | Over 200 | NR |
| (Appleby and Jones, 2011) | 2009-2010 | Subtrop. | Dingo | Rural | NR | Physical traps and motion-activated camera traps | Capture-mark-recapture with Lincolm-Petersen estimator (region-structured model) | Region-structured model based upon AIC comparisons | 130  (104 – 197) | NR |
| (Corbett, 1998) | 1998 | Subtrop. | UN | Rural | Without control | Reports and records, surveys, interviews (rangers, staff, visitors, residents), observations | Number of territories multiplied by the number of dingoes in a group | All dingoes (including pups) | NR | ~ 100 – ~ 200 |
| (Catling, 1978) | 1974-1976 | Temp. | Dingo | Rural | NR | Sand plots | NR |  | NR | 8 – 12 |
|  |  |  |  |  |  | Radio telemetry | NR |  | NR | 12 – 15 |

General abbreviations: NA = Not applicable; NR = Not reported

Climate zone abbreviations: Trop. = Tropical; Subtrop. = Subtropical; Temp. = Temperate; Des. = Desert; Grass. = Grassland

Type of dog abbreviations: UN = Unknown sample

The symbol ~ is used when the data is approximated

**Table E3**. Quantitative estimates extracted from 12 studies, captured in a scoping review, investigating contact or interactions between Australian dingoes and wild-living dogs

| **Study reference** | **Year** | **Climate** | **Type of dog** | **Type of environment** | **Dog control** | **Method of data collection** | **Parameter and method for contact or interaction measurement** | **Estimate** | | |
| --- | --- | --- | --- | --- | --- | --- | --- | --- | --- | --- |
|  |  |  |  |  |  |  |  | **Specification on estimate** | **Mean estimate ± SE or SD** | **Range** |
| (Newsome et al., 2013b) | 2008-2010 | Grass. | Dingo | Rural and mines | NR | Radio-tracking with GPS data logger | Map illustrating the overlaps of minimum convex polygon (95%) of home ranges for individual dingoes |  | NR | NR |
| (Thomson, 1992b) | 1975-1984 | Grass. | Dingo | Rural | Both control | Radio-tracking and observations from aircraft | Percentage of non-repetitive records with intraspecific interactions between active adult dingoes |  | NR | ~4.1% – ~6.9% |
| (Thomson, 1992c) | 1975-1978 | Grass. | Dingo | Rural | Without control | Radio-tracking and observations from aircraft | Observations and descriptions of encounters between packs and between lone dingoes and members of packs |  | NR | NR |
|  |  |  |  |  |  |  | Map illustrating the overlaps of 1) minimum convex polygon (95%) of home ranges for individual dingoes, 2) pack territories (Minimum convex polygon 95%) and 3) pack territories and living areas of lone dingoes |  | NR | NR |
| (Thomson et al., 1992a) | 1975-1984 | Grass. | Dingo | Rural | With control | Radio-tracking and observations from aircraft | Observations and descriptions of encounters between packs or between pack members and non-members |  | NR | NR |
|  |  |  |  |  |  |  | Map illustrating the overlaps of packs ranges based on 95% convex polygons |  | NR | NR |
| (Corbett and Newsome, 1975) | 1966-1975 | Grass., Des. | Dingo | Rural | NR | Observations at watering points and telemetry | Observations and descriptions of encounters between dingoes |  | NR | NR |
| (Sparkes et al., 2016) | 2013-2015 | Subtrop. | Mixed | Rural | NR | Camera traps | Contact rates per camera trap nights |  | 0.2 ± 0.2 (sd) | 0.02 – 0.56 |
| (Brook, 2013d) | 2011-2012 | Grass. | Dingo | Rural | NR | GPS tracking | UDOI, using utilisation distributions at the MKDE 95% isopleth |  | 0.22 ± 0.12 (se) | NR |
|  |  |  |  |  |  |  | UDOI, using utilisation distributions at the MKDE core isopleth |  | 0.04 ± 0.02 (se) | NR |
|  |  |  |  |  |  |  | UDOI, using intensity distributions at the MKDE core isopleth |  | 0.01 ± 0.01 (se) | NR |
|  |  |  |  |  |  |  | UDOI, using recursion distribution at the MKDE core isopleth |  | 0.05 ± 0.03 (se) | NR |
|  |  |  |  |  |  |  | PHR using utilisation Distributions at the MKDE 95% isopleth |  | 0.19 ± 0.05 (se) | NR |
|  |  |  |  |  |  |  | PHR, using utilisation distributions at the MKDE core isopleth |  | 0.08 ± 0.03 (se) | NR |
|  |  |  |  |  |  |  | PHR, using intensity distributions at the MKDE core isopleth |  | 0.05 ± 0.02 (se) | NR |
|  |  |  |  |  |  |  | PHR, using recursion distribution at the MKDE core isopleth |  | 0.11 ± 0.04 (se) | NR |
| (Purcell, 2008a) | 2005-2007 | Temp. | Dingo, hybrid | Rural | With control | GPS tracking | Maps of individual home range overlaps based on minimum convex polygons and kernel contours |  | NR | NR |
| (Bird, 1994) | 1991 | Des. | Dingo | Rural | With control | Direct observations of behavior of wild dingoes | Observations and descriptions of attacks and interactions between dingoes |  | NR | NR |
| (Meek and Brown, 2016) | 2015-2016 | Des. | Dingo | Mine | NR | Camera trap observations at leg-hold trap sites | Observations and descriptions of encounters and aggressive interactions between trapped and other dingoes |  | NR | NR |
| (Lawrance and Higginbottom, 2002) | 2000 | Subtrop. | Dingo | Rural | Without control | Direct observations in the field | Number of interactions observed between dingoes per hour | High human use environment | 0 time per hour | NR |
|  |  |  |  |  |  |  |  | Low human use environment | 10 times per hour | NR |
| (Corbett, 1988) | 1973-1975 | NR | Dingo | Captive | NA | Direct observations of interactions between captive dingoes | Mean hourly frequency of interactions | Social interactions | NR | 0 – ~23 |
|  |  |  |  |  |  |  |  | Aggressive interactions | NR | 0 – ~12 |

General abbreviations: NA = Not applicable; NR = Not reported

Climate zone abbreviations: Trop. = Tropical; Subtrop. = Subtropical; Temp. = Temperate; Des. = Desert; Grass. = Grassland

Method of data calculation abbreviations: UDOI = Utilisation distribution overlap index; PHR = Probability home range overlap index; MKDE = Movement-based kernel density estimates

The symbol ~ is used when the data is approximated
